# A statistical model for the analysis of beta values in DNA methylation studies

**DOI:** 10.1186/s12859-016-1347-4

**Published:** 2016-11-22

**Authors:** Leonie Weinhold, Simone Wahl, Sonali Pechlivanis, Per Hoffmann, Matthias Schmid

**Affiliations:** 1 Department of Medical Biometry, Informatics and Epidemiology, University of Bonn, Sigmund-Freud-Str. 25, Bonn, D-53127 Germany; 2 Research Unit of Molecular Epidemiology, Helmholtz Zentrum München, Ingolstädter Landstr. 1, Neuherber, D-85764 Germany; 3 Department of Medical Informatics, Biometry and Epidemiology, University Hospital Essen, Hufelandstr. 55, Essen, D-45122 Germany; 4 Human Genomics Research Group, Department of Biomedicine, University Hospital Basel, Hebelstr. 20, Basel, CH-4031 Switzerland

**Keywords:** Bounded response variables, DNA methylation, Gamma Regression, Gradient Boosting, HumanMethylation450k BeadChip

## Abstract

**Background:**

The analysis of DNA methylation is a key component in the development of personalized treatment approaches. A common way to measure DNA methylation is the calculation of beta values, which are bounded variables of the form *M*/(*M*+*U*) that are generated by Illumina’s 450k BeadChip array. The statistical analysis of beta values is considered to be challenging, as traditional methods for the analysis of bounded variables, such as M-value regression and beta regression, are based on regularity assumptions that are often too strong to adequately describe the distribution of beta values.

**Results:**

We develop a statistical model for the analysis of beta values that is derived from a bivariate gamma distribution for the signal intensities *M* and *U*. By allowing for possible correlations between *M* and *U*, the proposed model explicitly takes into account the data-generating process underlying the calculation of beta values. Using simulated data and a real sample of DNA methylation data from the Heinz Nixdorf Recall cohort study, we demonstrate that the proposed model fits our data significantly better than beta regression and M-value regression.

**Conclusion:**

The proposed model contributes to an improved identification of associations between beta values and covariates such as clinical variables and lifestyle factors in epigenome-wide association studies. It is as easy to apply to a sample of beta values as beta regression and M-value regression.

**Electronic supplementary material:**

The online version of this article (doi:10.1186/s12859-016-1347-4) contains supplementary material, which is available to authorized users.

## Background

The analysis of DNA methylation has become of considerable interest in biomedical research, as epigenetic studies have shown numerous associations between methylation levels and diseases such as cancer and cardiovascular disease [1–5]. Today, most research focuses on the cytosine-guanine dinucleotide (“CpG”) sites of the DNA, which are the locations where methylation is primarily found in humans [6]. One of the most widely used techniques to measure DNA methylation is the Illumina Infinium HumanMethylation450 BeadChip array, which covers approximately 450,000 CpG sites. At each CpG site, methylation is quantified by the beta value *b*:=*M*/(*M*+*U*+*a*), where *M*>0 and *U*>0 denote the methylated and unmethylated signal intensities, respectively, measured by the Illumina 450k array. The offset *a*≥0 is usually set equal to 100 and is added to *M*+*U* to stabilize beta values when both *M* and *U* are small.

An important goal of methylation analysis is to identify DNA regions where methylation is associated with disease status, lifestyle factors and other clinical or sociodemographic variables [7–10]. This is often achieved by fitting site-wise regression models with dependent variable *b* and a vector of covariates ***X*** that may also include potential confounders. After model fitting, a common strategy is to carry out downstream hypothesis tests to identify those CpG sites that show significant associations between methylation status and the variables of interest.

Because, by definition, *b* is bounded between 0 and 1, Gaussian regression with untransformed beta values is problematic in the context of DNA methylation analysis. In particular, the variance of *b* is usually smaller near the boundaries than near the middle of the interval (0,1), implying that the homoscedasticity assumption in Gaussian regression is violated [11–13]. To address this problem, several modeling strategies have been developed, including Gaussian regression with logit-transformed beta values (“M-values”, [11]) and generalized regression models for untransformed bounded responses, e.g. beta regression [14]. Regarding the analysis of DNA methylation, both strategies may become problematic: In case of M-value regression, the assumptions of a Gaussian model are often not met despite the transformation of the data, and the interpretation of the coefficient estimates is only possible on the transformed scale but not on the original scale of *b* [14, 15]. Beta regression, on the other hand, requires the ratio *M*/(*M*+*U*+*a*) to follow a beta distribution, implicitly assuming that the variables *M* and *U* are independently gamma distributed [16]. While *M* and *U* can indeed be described by gamma distributed random variables [17, 18], the independence assumption for the two signal intensities is often not met in practice. For example, Laird [12] reported that the methylated and unmethylated signal intensities, as produced by the Illumina 450k array, are usually positively correlated. The same finding was obtained from the analysis of the Heinz Nixdorf Recall Study data in the “Results” section of this article. These issues, along with the results of two recent empirical studies [8, 18], suggest that more methodological research is needed to describe the distribution of *b* in a statistically sound way.

To address this problem, we propose a novel analysis technique for beta values that relaxes the independence assumption between the signal intensities *M* and *U*. The idea is to start with a model for the bivariate distribution of *M* and *U* and to derive the probability density function of the ratio *M*/(*M*+*U*). This function is subsequently used to construct the log-likelihood function of a generalized regression model that relates beta values to linear functions of the covariates. Because estimation of the model parameters is based on the maximum likelihood principle, asymptotic confidence intervals and normally distributed test statistics can be derived by evaluating the inverse of the observed information matrix. This strategy allows for downstream hypothesis tests on the associations between a covariate of interest and the methylation status at individual CpG sites. For the rest of this article, we will refer to the proposed model as “RCG” (*R*atio of *C*orrelated *G*ammas) model.

Using simulated data and a real sample of Illumina 450k methylation data from the Heinz Nixdorf Recall (HNR) cohort study [19], we demonstrate that the proposed RCG model fits our data significantly better than beta regression and M-value regression (“Results” section). Our numerical results further suggest that the RCG method may lead to an increased power of downstream tests on the association(s) between methylation status and the covariates under consideration.

## Methods

In section “Notation and definitions” we introduce basic notation and definitions. Section “Regression models for the analysis of beta values” briefly reviews beta regression and M-value regression and discusses the limitations of the two methods. In the section “A statistical model for the ratio of correlated gamma distributed random variables” the proposed RCG model for the analysis of beta values is derived. Section “Estimation and hypothesis tests” provides details on model fitting and on the construction of downstream hypothesis tests.

### Notation and definitions

At each CpG site, the Illumina 450k array produces a sample of methylated and unmethylated signal intensities (*M*_*i*_,*U*_*i*_)_*i*=1,…,*n*_, where *n* is the number of analyzed persons. The corresponding set of beta values is calculated by *b*_*i*_=*M*_*i*_/(*M*_*i*_+*U*_*i*_+*a*), *i*=1,…,*n*. To facilitate the derivation of distributional results, we will set *a*=0 throughout this section. The predictor variable(s) of interest and the confounding variables are collected in vectors ***X***_*i*_=(1,*X*_*i*1_,…,*X*_*ip*_)^⊤^, *i*=1,…,*n*. For each CpG site, the aim is to analyze the associations between the variables in ***X*** and the methylation status *b*.

Following [17] and [18], we assume that the stochastic behavior of the signal intensities *M* and *U* can be described by gamma distributed random variables with densities

1 $$\begin{array}{@{}rcl@{}}  f_{M} (m) &=& \frac{\lambda_{m}}{\Gamma (\alpha_{m})} \left(\lambda_{m} m \right)^{\alpha_{m} - 1} \exp (-\lambda_{m} m), \end{array} $$

2 $$\begin{array}{@{}rcl@{}} f_{U} (u) &=& \frac{\lambda_{u}}{\Gamma (\alpha_{u})} \left(\lambda_{u} u \right)^{\alpha_{u} - 1} \exp (-\lambda_{u} u), \end{array} $$

where *α*_*m*_,*α*_*u*_ and *λ*_*m*_,*λ*_*u*_ are the shape and rate parameters of *f*_*M*_ and *f*_*U*_, respectively. From (1) it follows that the means and variances of *M*, *U* are given by *α*_*m*_/*λ*_*m*_, *α*_*u*_/*λ*_*u*_ and $\alpha _{m} / {\lambda _{m}^{2}}$, $\alpha _{u} / {\lambda _{u}^{2}}$, respectively [20].

### Regression models for the analysis of beta values

Since the ratio *b*=*M*/(*M*+*U*) is bounded between 0 and 1, it has been argued that a linear regression model of the form

3 $$ b = \boldsymbol{X}^{\top} \gamma + \epsilon \,, \ \ \, \gamma \in \mathbb{R}^{p+1} \,, \ \ \, \epsilon \sim \mathcal{N}\left(0, \sigma^{2}\right) \,,  $$

is not appropriate to model DNA methylation. In particular, the variance of *b* is usually smaller near the boundaries than near the middle of the interval (0,1), implying that the homoscedasticity assumption var(*ε*)=*σ* ^2^ is violated [11].

In view of this problem, several statistical models for bounded response variables have been developed (see [13] for an overview). A simple approach is to calculate logit-transformed beta values (“M-values”, [11]) and to fit a linear regression model of the form

4 $$ \log_{2} \left(\frac{b}{1 - b} \right) = \boldsymbol{X}^{\top} \gamma + \epsilon \,, \ \ \, \epsilon \sim \mathcal{N}\left(0, \sigma^{2}\right) \,.  $$

Although this strategy has become popular in the analysis of DNA methylation, it has the drawback that the methylation status (as quantified by the value of *b*) is not analyzed on its original scale but on a transformed scale [14]. Furthermore, as shown by Wahl et al. [8], the empirical distribution of logit-transformed beta values usually deviates from normality.

An alternative approach that operates on the untransformed scale of *b* is *beta regression*, which is characterized by a beta distributed outcome variable with probability density function

5 $$  \varphi (b) =\, \frac{\Gamma (\phi) }{\Gamma (\mu \phi) \Gamma ((1-\mu)\phi)}\, b^{\mu\phi - 1}\, (1-b)^{(1-\mu)\phi - 1} \,,  $$

where *μ* and *ϕ* denote the mean and precision parameters, respectively, of the probability density function *φ*. The predictor-response relationship is usually defined by a monotone increasing link function *g*(·) and by the model equation *g*(*μ*|***X***)=***X*** ^⊤^ *γ* [14]. A common choice for *g* is the logit transformation log(*μ*/(1−*μ*)). Since the variance of a beta distributed random variable is given by *μ*(1−*μ*)/ (1+*ϕ*), beta regression accounts for heteroscedasticity and for small variances near the boundaries of the interval (0,1). On the other hand, a major shortcoming of (5) in the context of DNA methylation analysis is that the signal intensities *M* and *U* are implicitly assumed to be independent and to share a common rate parameter. Under these assumptions, the ratio *b*=*M*/(*M*+*U*) can be shown to follow a beta distribution ([16], Chapter 9). The independence assumption, however, cannot be confirmed by empirical findings, which show that the signal intensities obtained from the Illumina 450k array are often positively correlated (see [12] and “Analysis of the Heinz Nixdorf recall study data” section of this article).

### A statistical model for the ratio of correlated gamma distributed random variables

To address the issues described in the section “Regression models for the analysis of beta values”, we propose a statistical model (“Ratio of Correlated Gammas (RCG) model”) that is based on the bivariate distribution of the signal intensities *M* and *U*. In contrast to beta regression, we assume that *M* and *U* are not independent but can be described by a bivariate gamma distribution with probability density function

6 $$ \begin{aligned} f_{M,U}(m,u) &= \frac{(\lambda_{m}\lambda_{u})^{\alpha}}{(1-\rho)\, \Gamma(\alpha)}\left(\frac{mu}{\rho\,\lambda_{m}\lambda_{u}}\right)^{\frac{\alpha-1}{2}} \exp{\left(-\frac{\lambda_{m} m}{1-\rho}\right)} \, \\[.1cm] & \quad \times \, \exp{\left(-\frac{\lambda_{u} u}{1-\rho}\right)} I_{\alpha-1}\left(\frac{2\sqrt{\rho\lambda_{m}\lambda_{u} mu}}{1-\rho}\right) \,, \end{aligned}  $$

where *λ*_*m*_,*λ*_*u*_,*α*>0, 0<*ρ*<1, and *I*_*α*−1_ is the modified Bessel function of the first kind of order *α*−1. The distribution in (6) is due to Kibble [21] and is often referred to as “Wicksell-Kibble bivariate gamma distribution” [20]. As stated in various articles and monographs (e.g. [22]), the marginal densities *f*_*M*_, *f*_*U*_ of *M* and *U*, respectively, are given by

7 $$\begin{array}{@{}rcl@{}}  f_{M} (m) &=& \frac{\lambda_{m}}{\Gamma (\alpha)} \left(\lambda_{m} m \right)^{\alpha - 1} \exp (-\lambda_{m} m), \end{array} $$

8 $$\begin{array}{@{}rcl@{}} f_{U} (u) &=& \frac{\lambda_{u}}{\Gamma (\alpha)} \left(\lambda_{u} u \right)^{\alpha - 1} \exp (-\lambda_{u} u). \end{array} $$

The equations in (7) and (8) imply that *M* and *U* are gamma distributed random variables with a common shape parameter *α* and with means and variances given by *α*/*λ*_*m*_, *α*/*λ*_*u*_ and $\alpha / {\lambda _{m}^{2}}$, $\alpha / {\lambda _{u}^{2}}$, respectively. The restriction to a common shape parameter ensures that all measured signal intensities refer to probability density functions sharing the same basic form. On the other hand, the unequal rate parameters *λ*_*m*_ and *λ*_*u*_ guarantee sufficient flexibility in modeling the differences in the marginal densities of *M* and *U* (see (11) and (12)). It can further be shown that the Pearson correlation of *M* and *U* is equal to *ρ*, implying that (6) imposes a correlation structure on the two signal intensities (see [20]).

In the next step, the distribution of the ratio *b*=*M*/(*M*+*U*) is derived:

#### **Proposition 1**

Let the distribution of (*M,U*) be defined by the probability density function in (6). Then the ratio *b*=*M*/(*M*+*U*) follows a univariate distribution with probability density function

9 $$ \begin{aligned} f_{b} (b) & = \frac{\Gamma(2\alpha)}{\Gamma^{2}(\alpha)} \, (\lambda_{m}\lambda_{u})^{\alpha} \, (1-\rho)^{\alpha} \, \left(b(1-b)\right)^{\alpha-1} \\ & \quad \times\, \frac{\left(\lambda_{m} b+\lambda_{u} (1-b)\right)}{\left(\left(\lambda_{m} b+\lambda_{u} (1-b)\right)^{2}-4\rho\lambda_{m}\lambda_{u} b(1-b)\right)^{\alpha+0.5}} . \end{aligned}  $$

#### *Proof*

The proof of Proposition 1, which is related to the work of Nadarajah and Kotz [23], is given in Additional file 1. □

The result stated in Proposition 1 can be used to derive the log-likelihood function of a sample of beta values *b*_1_,…,*b*_*n*_:

#### **Proposition 2**

For independent sample values *b*_1_,…,*b*_*n*_, the log-likelihood function derived from (9) is given by

10 $$ {{}{\begin{aligned} \sum_{i=1}^{n} \log(f_{b} (b_{i}; \alpha, \rho, \theta)) &= \sum_{i=1}^{n} \left[ \log(\Gamma(2\alpha)) -2\log \left(\Gamma(\alpha)\right) \right. \\ & \quad \left. +\, \alpha\log\left(\theta\right) +\,\alpha\log\left(1-\rho\right) \right. \\ & \quad \left. + \log\left(\left(\theta-1\right)b_{i} + 1\right) \right. \\ & \quad \left. + \left(\alpha-1\right)\log\left(b_{i}\left(1-b_{i}\right)\right) \right. \\[.1cm] & \quad \left. - \left(\alpha+0.5\right) \log \left(\left(\left(\theta-1\right)b_{i} + 1\right)^{2} \right. \right. \\ & \quad \left.\left. -\, 4 \rho \theta \,b_{i} (1-b_{i}) \right){\vphantom{\log(\Gamma(2\alpha)) -2\log}}\! \right] \,, \\[.1cm] \end{aligned}}}  $$

where *θ*:=*λ*_*m*_/*λ*_*u*_.

#### *Proof*

See Additional file 1.

Proposition 2 implies that the log-likelihood function derived from (9) is a function of the mean ratio *θ*=*λ*_*m*_/*λ*_*u*_=E(*U*)/E(*M*).

To quantify the associations between the covariates ***X*** and the signal intensities *M* and *U*, we consider linear predictors ***X*** ^⊤^ *ζ*_*m*_ and ***X*** ^⊤^ *ζ*_*u*_, $\zeta _{m}, \zeta _{u} \in \mathbb {R}^{p+1}$, that relate the vector ***X***=(1,*X*_1_,…,*X*_*p*_)^⊤^ to the marginal means *α*/*λ*_*m*_ and *α*/*λ*_*u*_, respectively. A convenient link function that guarantees the positivity of *λ*_*m*_ and *λ*_*u*_ is the logarithmic transformation, resulting in the predictor-response relationships

11 $$\begin{array}{@{}rcl@{}}  \log (\mathrm{E}(M| \boldsymbol{X})) &=& \log ({\alpha}) - \boldsymbol{X}^{\top}\zeta_{m}, \end{array} $$

12 $$\begin{array}{@{}rcl@{}}  \log (\mathrm{E}(U| \boldsymbol{X})) &=& \log (\alpha) - \boldsymbol{X}^{\top}\zeta_{u}, \end{array} $$

with log(*λ*_*m*_)=***X*** ^⊤^ *ζ*_*m*_ and log(*λ*_*u*_)=***X*** ^⊤^ *ζ*_*u*_. Note that the term log(*α*) can be incorporated into the intercept terms of the coefficient vectors *ζ*_*m*_=(*ζ*_0*m*_,*ζ*_1*m*_,…,*ζ*_*pm*_)^⊤^ and *ζ*_*u*_=(*ζ*_0*u*_,*ζ*_1*u*_,…,*ζ*_*pu*_)^⊤^. The model equations in (11) and (12) are therefore in line with traditional univariate gamma regression approaches that relate the log-transformed mean of the response variable to a linear function of the predictors.

Defining *γ*=(*γ*_0_,*γ*_1_,…,*γ*_*p*_)^⊤^:=*ζ*_*m*_−*ζ*_*u*_, the mean ratio E(*U*|***X***)/E(*M*|***X***) can be written as *θ*|***X***= exp(***X*** ^⊤^ *γ*), and the log-likelihood function of a sample $(b_{1}, \boldsymbol {X}_{1}^{\top }), \ldots, (b_{n}, \boldsymbol {X}_{n}^{\top })$ becomes

13 $$ \begin{aligned} \sum_{i=1}^{n} \log(f_{b} (b_{i}, \boldsymbol{X}_{i}; \alpha, \rho, \gamma)) & = \sum_{i=1}^{n} \left[ \log(\Gamma(2\alpha))-2\log (\Gamma(\alpha)) +\, \alpha\,\boldsymbol{X}^{T}_{i}\gamma\right. \\ & \quad + \alpha\log(1 \,-\, \rho) \,+\, \log \!\left(\! \left(\! \exp \! \left(\! \boldsymbol{X}^{T}_{i}\gamma \! \right)\! -\! 1\! \right) b_{i} \,+\, 1 \! \right) \\ & \quad + (\alpha-1)\, \log(b_{i}(1-b_{i})) \\[.1cm] & \quad \left. - \left(\alpha \,+\, 0.5 \right) \log \! \left(\! \left(\! \left(\exp \! \left(\boldsymbol{X}^{T}_{i}\gamma \! \right)\! -\! 1\right)\! \, b_{i} \,+\, 1\right)^{2} \right. \right. \\ & \quad \left.\left. -\, 4 \, \rho \exp \left(\boldsymbol{X}^{T}_{i}\gamma \right)\, b_{i}(1-b_{i}) \left(\boldsymbol{X}^{T}_{i}\gamma \! \right)\right) {\vphantom{\left(\boldsymbol{X}^{T}_{i}\gamma \! \right)}}\right] \,. \end{aligned}  $$

Equations (11) to (13) define a statistical model in which the association between the methylation status *b* and the covariates ***X*** is quantified by the coefficient vector *γ*. If *γ*_*k*_=0, *k*∈{1,…,*p*}, the predictor-response relationships in (11) and (12) imply that *ζ*_*km*_=*ζ*_*ku*_ and E(*M*|***X***)=E(*U*|***X***) (provided that the values of the other covariates remain constant). Hence, if *γ*_*k*_=0, the *k*-th covariate *X*_*k*_ has the same effect on both *M* and *U*, implying that *X*_*k*_ is not associated with the methylation status at the CpG site under consideration. On the other hand, large values of |*γ*_*k*_| result from large differences in the coefficients *ζ*_*km*_ and *ζ*_*ku*_, implying that DNA methylation varies greatly with the value of *X*_*k*_. Assessing the hypotheses “ *H*_0_ : *γ*_*k*_ = 0 vs. *H*_1_:*γ*_*k*_≠0” is therefore equivalent to a statistical test on the association between *b* and *X*_*k*_. □

### Estimation and hypothesis tests

To obtain a consistent estimator of the coefficient vector *γ*, the log-likelihood function in (13) needs to be maximized over both *γ* and the hyperparameters *α* and *ρ*. To this purpose, we propose the application of a gradient boosting algorithm with linear base-learning functions, as described in [24]. For given data $(b_{i}, \boldsymbol {X}^{\top }_{i})_{i=1,\ldots, n}$, gradient boosting is a generic optimizer that minimizes a risk function $\mathcal {R} (f, (b_{i}, \boldsymbol {X}_{i}^{\top })_{i=1,\ldots, n})$ over an unknown prediction function *f*(***X***), with the only requirement being the existence of the derivative $\partial \mathcal {R} / \partial f$ [25].

Because the base-learning functions are chosen to be linear in ***X***, the space of the prediction function *f* is restricted to the subspace defined by *f*(***X***)=***X*** ^⊤^ *γ*, implying that estimation of *f* reduces to the estimation of the coefficient vector *γ* (see [26] for a detailed description of the algorithm). Furthermore, gradient boosting allows for the additional estimation of the hyperparameters *α* and *ρ* [27]. Maximum likelihood (ML) estimates of *γ*, *α* and *ρ* can therefore be obtained by setting $\mathcal {R}$ equal to the negative of the log-likelihood in (13) and by running gradient boosting until convergence.

By standard maximum likelihood arguments, the hypotheses “ *H*_0_:*γ*_*k*_=0 vs. *H*_1_:*γ*_*k*_≠0” can be investigated by plugging the ML estimates $\hat {\gamma }$, $\hat {\alpha }$ and $\hat {\rho }$ in the observed information matrix $J (\alpha, \rho, \gamma) = - \sum _{i=1}^{n} \partial ^{2} \log (f_{b} (b_{i}, \boldsymbol {X}_{i}; \alpha, \rho, \gamma)) / \partial ^{2} \gamma $ and by calculating the test statistic

14 $$  Z_{k} = \hat{\gamma_{k}}\, \left/ \sqrt{J^{-1}_{kk} (\hat{\alpha}, \hat{\rho}, \hat{\gamma})} \,, \ \ \, k \in \{ 1, \ldots, p\}\right. \,,  $$

where $J^{-1}_{kk}$ denotes the *k*-th diagonal element of *J* ^−1^. Under the null hypothesis, *Z*_*k*_ is asymptotically standard normally distributed as *n*→*∞*. Details on the calculation of *J* are given in Additional file 1.

## Results

### Description and pre-processing of the HNR study data

To investigate the properties of the RCG model derived in the section “A statistical model for the ratio of correlated gamma distributed random variables”, we analyzed both simulated data and a real sample of Illumina 450k methylation data from the Heinz Nixdorf Recall Study [19]. The HNR Study is an ongoing cohort study in the German cities of Mülheim, Essen and Bochum that enrolled a total of 4,814 participants aged 45-75 years between 2000 and 2003. Data collection included health, lifestyle and environmental variables; the 10-year follow-up of the study was completed in 2014.

For the present analysis, we considered a random sample of *n*= 1,144 study participants whose DNA samples were sodium-bisulfite converted and processed using Illumina Infinium HumanMethylation450 BeadChips v1.1. Processing was done according to the manufacturer’s manual on a fully automated iScan system between April 2013 and January 2015. Technical quality control was performed using GenomeStudio V2011.1.

Pre-processing of the methylation data was based on the R add-on package minfi [28]. Briefly, persons that contained >20 *%* low-confidence beta values (detection *P*-values >0.01) and CpG sites with more than 5 *%* low-confidence beta values were dropped. CpG sites that contained either a SNP at the CpG interrogation or at the single nucleotide extension were also excluded from statistical analysis. In addition, CpG sites referring to cross-reactive probes were removed, followed by the exclusion of X and Y chromosomal sites. Normalization of the beta values was carried out using the functional normalization algorithm [29], which was applied separately to type I and type II probes. The *k*-nearest-neighbor method with *k*=10 was used to impute missing beta values.

**Fig. 1:**
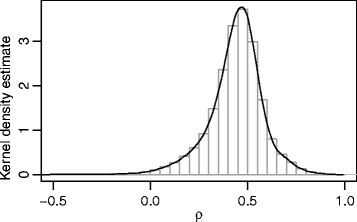
Analysis of the HNR Study data. The figure shows a kernel density plot of the Pearson correlations between the signal intensities *M* and *U* across the full set of 429,750 CpG sites

After pre-processing, a total of *n*= 1,118 persons and 429,750 CpG sites remained in the analysis set. The distribution of the 429,750 Pearson correlation coefficients between the signal intensities *M* and *U* is shown in Fig. 1. The majority of the coefficients was substantially larger than zero, indicating that the independence assumption for *M* and *U* was not justified. More than 99.2 *%* of the correlation coefficients were positive (mean = 0.452, sd = 0.140).

In addition to the beta values, we considered the covariates “gender” (47.9 *%* women), “age” (mean = 58.3 years, sd = 7.3 years), “body mass index” (mean = 27.4 kg/m^2^, sd = 7.3 kg/m^2^), “CES-D depression score” ([30], median = 6, interquartile range 3–10) and “smoking status” (18.9 *%* smokers). We selected these covariates because they are generally considered to be relevant for the analysis of DNA methylation (e.g. [10, 31, 32]).

### Simulation study

One of the main goals of a DNA methylation study is to identify CpG sites that are “significantly” associated with one or more covariates of interest. We therefore conducted a simulation study on the power of downstream hypothesis tests, as obtained from M-value regression, beta regression and the proposed RCG model.

#### Power analysis at a single CpG site

In the first part of the simulation study, we considered a random CpG site (“cg00786084”) and based the study on the effect sizes obtained from the HNR Study data (RCG model with five covariates). The maximum likelihood estimates were

$$\begin{array}{@{}rcl@{}} \hat{\gamma} &=& (\hat{\gamma}_{0}, \hat{\gamma}_{\text{gender}}, \hat{\gamma}_{\text{age}}, \hat{\gamma}_{\text{bmi}}, \hat{\gamma}_{\text{smoke}}, \hat{\gamma}_{\text{depression}})^{\top} \\ &=& (-1.099, 0.096, -0.007, -0.004, 0.003, 0.001)^{\top} \,, \end{array} $$

$\hat {\alpha }=5.84$ and $\hat {\rho }=0.93$. Setting the values of *α*,*γ*_0_,*γ*_age_,*γ*_bmi_,*γ*_smoke_ and *γ*_depression_ equal to the maximum likelihood estimates and using the covariate values of the HNR Study data (*n*= 1,118), we calculated the linear predictors ***X*** ^⊤^ *γ* for varying values of *γ*_gender_. Three values of *ρ* were considered (0.2, 0.5 and 0.93, the latter value being the original sample estimate). For each combination of *γ*_gender_ and *ρ*, we generated 10,000 beta values from the distribution of the ratio in (9). Based on the simulated beta values and the real covariate values of the HNR Study, we analyzed the power of the test on the hypotheses “ *H*_0_:*γ*_gender_=0 vs. *H*_1_:*γ*_gender_≠0”. For the RCG model we used the asymptotic test described in the section “Estimation and hypothesis tests”. Beta regression and M-value regression models were also analyzed using test statistics of the form (14).

**Fig. 2:**
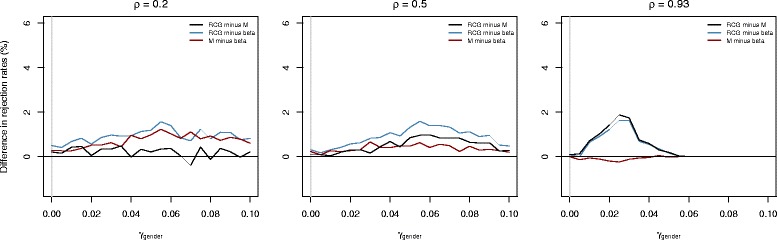
Results obtained from the first part of the simulation study. The plots show the differences in the estimated rejection rates of the null hypothesis “ *H*_0_:*γ*_gender_=0”, as obtained from the RCG model, beta regression, and M-value regression (10,000 simulation runs). The covariate values of the HNR Study (*n*= 1,118) were used to generate the linear predictors ***X*** ^⊤^ *γ*. Beta values were generated from the distribution of the ratio in (9) using the sample estimates at CpG site cg00786084. High levels of the black and blue lines correspond to a high power of the RCG-based tests. The vertical gray line refers to the null hypothesis *H*_0_

Figure 2 shows the differences in the fractions of tests that rejected the null hypothesis “ *H*_0_:*γ*_gender_=0” at the 5 *%* level for varying values of *γ*_gender_ and *ρ*. It is seen that the RCG model performed better than beta and M-value regression, especially in situations where the effect size *γ*_gender_ took moderately high values. For large effect sizes, the power of the three models was similar. This result is explained by the fact that large effect sizes resulted in high rejection rates of the null hypothesis “ *H*_0_:*γ*_gender_=0” regardless of whether the correlation between signal intensities was taken into account or not. As expected, the differences between the RCG model and competing approaches increased with the value of *ρ*. At the same time, RCG-based type I error rates were close to the nominal level of significance (0.054,0.049,0.050 for *ρ*=0.2,0.5,0.93, respectively).

#### Sensitivity analysis

In the second part of the simulation study, we carried out a sensitivity analysis and investigated the power of downstream hypothesis tests in situations where the bivariate distribution of the signal intensities *M* and *U* deviated from the Wicksell-Kibble distribution. To this purpose, we repeated the analysis of CpG site cg00786084 and generated correlated gamma distributed signal intensities using a copula-based approach. More specifically, we generated standard uniformly distributed values $(\tilde {M}_{i},\tilde {U}_{i})$, *i*=1,…,1118, from a Gumbel copula of the form

15 $$ {}C (\tilde{M}, \tilde{U}) = \exp \left(- \left((- \log (\tilde{M}))^{\tilde{\rho }}+ (- \log (\tilde{U}))^{\tilde{\rho }} \right)^{1/{\tilde{\rho }}} \right) \,.  $$

**Fig. 3:**
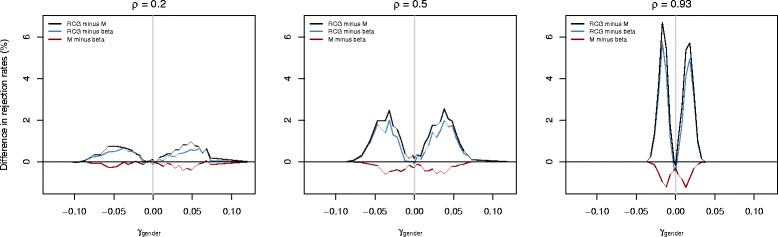
Results obtained from the second part of the simulation study. The plots show the differences in the estimated rejection rates of the null hypothesis “ *H*_0_:*γ*_gender_=0”, as obtained from the RCG model, beta regression, and M-value regression (10,000 simulation runs). The covariate values of the HNR Study (*n*= 1,118) were used to generate the linear predictors ***X*** ^⊤^ *γ*. Beta values were generated from a Gumbel copula using the sample estimates at CpG site cg00786084. High values in the black and blue lines correspond to a high power of the RCG-based tests. The vertical gray line refers to the null hypothesis *H*_0_

The parameter $\tilde {\rho }$ was adjusted such that $\tilde {M}$ and $\tilde {U}$ had the desired correlation (*ρ*∈{0.2,0.5,0.93}). Setting *ζ*_*m*_ and *ζ*_*u*_ equal to the sample estimates, i.e.

$$\begin{aligned} {\zeta}_{m} &= ({\zeta}_{0,m}, {\zeta}_{\text{gender},m}, {\zeta}_{\text{age},m}, {\zeta}_{\text{bmi},m}, {\zeta}_{\text{smoke},m}, {\zeta}_{\text{depression},m})^{\top} \\ & = (-6.2777, {\zeta}_{\text{gender},m}, 0.0003, - 0.0001, -0.0199, 0.0010)^{\top}, \\ {\zeta}_{u} &= ({\zeta}_{0,u}, {\zeta}_{\text{gender},u}, {\zeta}_{\text{age},u}, {\zeta}_{\text{bmi},u}, \zeta_{\text{smoke},u}, {\zeta}_{\text{depression},u})^{\top} \\ &= (-5.6424, -0.0731, 0.0088, 0.0016, -0.0073, - 0.0014)^{\top}, \end{aligned} $$

the values of $(\tilde {M},\tilde {U})$ were transformed to (0,*∞*) by applying the quantile functions of two gamma distributions with shape parameters *α*_*m*_=20.2, *α*_*u*_=12.760 and rate parameters *λ*_*m*_= exp(***X*** ^⊤^ *ζ*_*m*_), *λ*_*u*_= exp(***X*** ^⊤^ *ζ*_*m*_). The coefficient *ζ*_gender,*m*_ was varied such that it resulted in the values of *γ*_gender_ in Fig. 3. The transformed values of $(\tilde {M},\tilde {U})$ (representing the gamma distributed signal intensities *M* and *U*) were used to calculate 10,000 beta values for each combination of *ρ* and *ζ*_gender,*m*_.

Figure 3 shows the differences in the fractions of tests that rejected the null hypothesis “ *H*_0_:*γ*_gender_=0” at the 5 *%* level. Similar to the results presented in Fig. 2, the RCG model performed better than beta and M-value regression with regard to the power of downstream hypothesis tests. The differences between the models were even stronger than in the first part of the simulation study, despite the fact that the distribution of the beta values deviated from the Wicksell-Kibble distribution. Again, RCG-based type I error rates were close to the nominal level of significance (0.051,0.050,0.049 for *ρ*=0.2,0.5,0.93, respectively).

#### Extension to 1,000 CpG sites

In the third part of the simulation study, we extended the power analysis of part one and investigated the behavior of downstream hypothesis tests using 1,000 “real-life” combinations of the parameters *α*, *ρ* and *γ*. To this purpose, we randomly selected 1,000 CpG sites and fitted the respective RCG models to the HNR Study data. At each site, we used the RCG estimates to generate 1,000 beta values from the distribution of the ratio in (9). Using the 1,000 ×1,000 beta values, we estimated the power of the test on the hypotheses “ *H*_0_:*γ*_gender_=0 vs. *H*_1_ : *γ*_gender_ ≠ 0” at each CpG site.

**Fig. 4:**
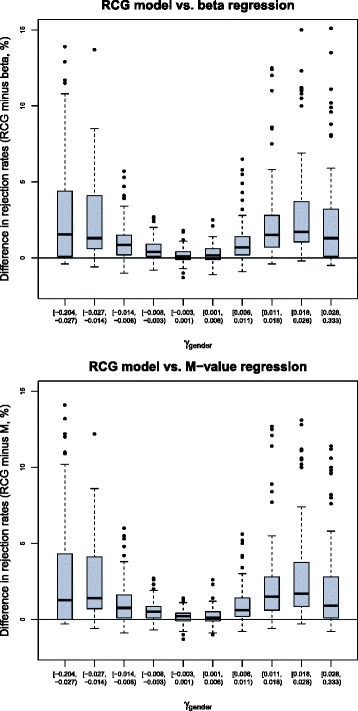
Results obtained from the third part of the simulation study. The boxplots contain the differences in the estimated rejection rates of the null hypothesis “ *H*_0_:*γ*_gender_=0”, as obtained from the RCG model, beta regression, and M-value regression. The grouping on the x-axes refers to the deciles of the effect *γ*_gender_ at 1,000 randomly selected CpG sites. The covariate values of the HNR Study (*n*= 1,118) were used to generate the linear predictors ***X*** ^⊤^ *γ*. Beta values were generated from the distribution of the ratio in (9). The estimated power of the RCG model was 0.053 in the interval [−0.003,0.001], indicating that the RCG-based type I error rates were close to the nominal level of significance

Figure 4 visualizes the fractions of tests that rejected the null hypothesis “ *H*_0_:*γ*_gender_=0”. The upper panel shows the differences in the rejection rates obtained from the RCG model and from beta regression, whereas the lower panel depicts the respective differences between the RCG model and M-value regression. The RCG model performed better than beta and M-value regression at the majority of the 1,000 CpG sites. Again, the differences between the RCG model and competing approaches were largest for moderate sizes of *γ*_gender_.

### Analysis of the Heinz Nixdorf recall study data

#### Analysis of model fit

In the first part of the analysis, we analyzed and compared the fits obtained from the RCG, beta regression and M-value regression models. To this purpose, the data were randomly subdivided into ten pairs of training and test data sets, each of sizes 750 and 368, respectively. Using all five covariates, RCG, beta regression and M-value regression models were fitted to the ten learning data sets at each of the 429,750 CpG sites. To evaluate the model fits, we calculated the predictive log-likelihood values (“log-scores”) obtained from the respective test data sets. Being a “proper” performance measure, the log-score is maximized by the log-likelihood of the true data-generating model [33]. In addition to the full models, we also evaluated the intercept models (“null models”) that contained no covariates at all.

**Fig. 5:**
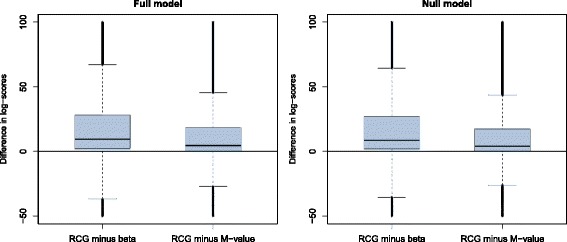
First part of the analysis of the HNR Study data. The boxplots show the average log-score differences obtained from beta regression, M-value regression and the RCG model. The left panel refers to the full models with five covariates, whereas the right panel refers to the covariate-free null models. At each of the 429,750 CpG sites, models were fitted to ten randomly sampled learning data sets of size *n*=750 each. Log-scores were calculated by evaluating the model fits on the respective independent test data sets (*n*=368). The boxplots refer to the 429,750 averages of the ten log-score differences

The average log-score differences obtained from beta regression, M-value regression and the RCG model are shown Fig. 5. The RCG model fitted the HNR Study data systematically better than beta and M-value regression (*P*-values of Wilcoxon signed rank tests <0.001). This result was obtained for both the full model containing all five covariates (left panel of Fig. 5) and the covariate-free null model (right panel of Fig. 5).

#### Rejection rates of downstream hypothesis tests

**Fig. 6:**
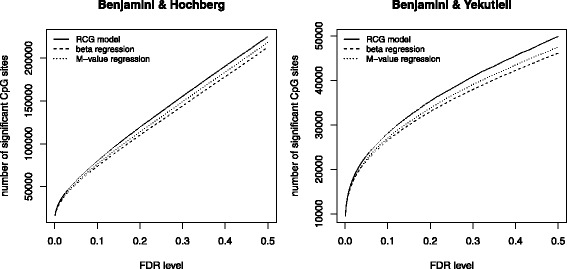
Second part of the analysis of the HNR Study data. The figure contains the number of “significant” associations between methylation status and gender, as obtained from beta regression, M-value regression and the RCG model at various levels of FDR control. *P*-values refer to the test of the hypotheses “ *H*_0_:*γ*_gender_=0 vs. *H*_1_:*γ*_gender_≠0” in the full model with all five covariates. The procedures by Benjamini & Hochberg and Benjamini & Yekutieli were used to correct the *P*-values for multiple comparisons. Note the different ranges of the y-axes

In the second part of the analysis, we reconsidered the 429,750 CpG sites analyzed in part one and calculated the *P*-values of downstream tests on the hypotheses “ *H*_0_:*γ*_gender_=0 vs. *H*_1_:*γ*_gender_≠0”. To correct the *P*-values for multiple comparisons, we applied the procedures by Benjamini & Hochberg and Benjamini & Yekutieli using various levels of false discovery rate (FDR) control. As demonstrated in Fig. 6, the number of “significant” associations was largest for the RCG model at the majority of FDR levels. For example, the numbers of “significant” CpG sites were 22,997, 22,199 and 21,779 for the RCG, M-value and beta regression models, respectively, at FDR = 0.05. These numbers are in line with earlier results by Singman et al. [10] who, after a Bonferroni correction of 391,885 *P*-values, identified 11,010 autosomal sex-methylation associations in the population-based KORA F4 study. Of note, our result implies that the application of the RCG model would have led to the discovery of (22,997 – 22,199) ≈800 additional significant CpG sites at the 5 % FDR level. We point out that the true number of non-zero associations among the 429,750 CpG sites is unknown, so that the aforementioned higher rejection rates obtained from the RCG model do not necessarily imply a higher true positive rate. Still, Fig. 6 is in line with the high(er) power obtained from the RCG-based tests in “Simulation study” section.

#### Analysis of CpG sites that are known to be associated with age and/or smoking behavior

In the third part of the analysis, we validated the RCG methodology by focusing on subsets of CpG sites that were previously reported to be associated with age or smoking behavior. The idea of this analysis was to investigate whether the RCG rejection rates at these “true positive” CpG sites were comparable to the respective rejection rates obtained from beta and M-value regression. Four subsets of CpG sites were considered: The first subset comprised the 187 smoking-associated CpG sites that were identified by Zeilinger et al. [34] in a replication sample of *n*=468 persons (*P*<5·10^−5^). Of these CpG sites, a total of 182 CpG sites were used in our analysis, as they passed the quality checks applied the HNR Study data. The second subset comprised the 215 smoking-associated CpG sites reported by Harlid et al. [35], of which 209 sites passed the quality checks applied to the HNR Study data. The third subset comprised the 162 age-associated CpG sites that were identified by Florath et al. [36] in a test sample of *n*=498 persons (*P*<2.5·10^−4^). Of these CpG sites, a total of 156 CpG sites passed the quality checks applied the HNR Study data. The fourth subset comprised the 589 age-associated CpG sites identified by Teschendorff et al. [37] (*n*=261, FDR <0.05), of which 536 sites passed the quality checks applied to the HNR study data. The full list of these CpG sites, which include numerous PCGT CpGs as well as CpGs mapping to AHRR, is contained in Additional file 2.

At each of the aforementioned CpG sites we analyzed the *P*-values obtained from the RCG method, M-value regression and beta regression. In a subsequent step we computed the rejection rates of the three methods in each subset. To ensure the comparability of the results across the four subsets, we used a global significance level of *P*<10^−7^, which was approximately equal to the Bonferroni-corrected 5 *%* alpha level [34]. The agreement between the rejection rates of the three modeling techniques was measured by the percentage of CpG sites with identical test results for the three techniques, and also by Cohen’s kappa.

The results, which are presented in full detail in Additional file 2, demonstrate a very high agreement between the RCG, M-value regression and beta regression models in each of the four subsets. The rejection rates obtained from the three methods were almost identical in each of the four subsets (∼55 *%* in the Zeilinger et al. subset, ∼49 *%* in the Harlid et al. subset, ∼96 *%* in the Florath et al. subset and ∼62 *%* in the Teschendorff et al. subset). The percentage of agreement ranged between 95.6 *%* and 98.7 *%*; Cohen’s kappa values ranged between 0.72 and 0.79 in the Florath et al. subset and were throughout larger than 0.91 in the Zeilinger et al., Harlid et al. and Teschendorff et al. subsets. These findings demonstrate that the RCG methodology resulted in a valid number of “true hits” at CpG sites with confirmed associations between methylation status and age / smoking behavior.

## Discussion and conclusions

The development of statistical models to analyze DNA methylation is the subject of intense and ongoing research [9, 38–40]. In this article, we proposed a likelihood-based approach to analyze and infer the associations between covariates and methylation levels in Illumina 450k data. In contrast to beta regression, the proposed RCG model accounts for possible correlations between methylated and unmethylated signal intensities, thereby increasing the flexibility of the model in describing the distribution of methylation levels at individual CpG sites. The analysis of the Heinz Nixdorf Recall Study data suggests that the RCG model fitted the data systematically better than traditional approaches like beta and M-value regression. This result is in line with our previous findings, which suggest that “at the majority of CpG sites, methylation follows neither a beta distribution, nor a normal distribution after any of the investigated transformations” [8].

In our simulation study, the RCG model resulted in higher true positive rates for the associations between DNA methylation and the covariates than beta and M-value regression. As expected, the differences between the RCG model and competing methods were largest at CpG sites with high correlations between methylated and unmethylated signal intensities. At the same time, the simulation study showed that RCG-based type I error rates were close to the nominal level of significance. By using combinations of site-wise *P*-values, it is straightforward to extend the RCG methodology to wider regions on the DNA such as CpG islands or island shores.

A crucial issue in DNA methylation analysis is the selection of an appropriate procedure for normalization and quality control [7, 41]. To this purpose, numerous pre-processing techniques have been developed, with the minfi pipeline used in this paper being a popular example. When deriving the RCG model, we implicitly assumed that all observed beta values were properly normalized and were observed without measurement error. While proper normalization and quality control should be taken for granted in any high-quality DNA methylation study, it might be worth investigating the effect of various pre-processing techniques on the behavior of the RCG model (and also on beta regression and M-value regression).

The use of a gradient boosting algorithm to optimize the parameters of the RCG model lays the ground for a variety of additional modeling options. For example, it is straightforward to account for nonlinear covariate effects and to extend the linear predictor in (13) by a set of spline functions. Furthermore, it is possible to embed the RCG model in the GAMLSS framework [42] and to increase its flexibility by relating the parameters *α* and *ρ* to separate linear or additive predictors. For details, see [43] and [44].

While the proposed RCG model was tested using methylation data from Illumina’s 450k array, we expect the analysis of beta values to gain even more importance with the recent launch of the Infinium MethylationEPIC BeadChip array covering more than 850,000 CpG sites [45].

## Additional files

Additional file 1 Contains distributional results and proofs of propositions. (PDF 54.1 kb)

Additional file 2 Contains the rejection rates at CpG sites that were previously reported to be associated with age and/or smoking. (XLSX 64.8 kb)

Additional file 3 R package mboostDevel. (GZ 2038 kb)

## Abbreviations

CpG: Cytosine-Guanine dinucleotide sites of the DNA
DNA: Deoxyribonucleic acid
FDR: False discovery rate
HNR: Heinz Nixdorf RECALL (risk factors, evaluation of coronary calcium and lifestyle)
RCG: Ratio of correlated Gammas
SNP: Single-nucleotide polymorphism
