# Supplementary material for: A statistical model for the analysis of beta values in DNA methylation studies
Source: BMC Bioinformatics. 2016 Nov 22;17:480. doi: 10.1186/s12859-016-1347-4 (PMC5120494; doi:10.1186/s12859-016-1347-4)
Supplement: Additional file 1 — Contains distributional results and proofs of propositions. (PDF 54.1 kb) [file 12859_2016_1347_MOESM1_ESM.pdf]

A Statistical Model for the Analysis of Beta Values in DNA Methylation Studies

L. Weinhold, S. Wahl, S. Pechlivanis, P. Hoffmann, M. Schmid

Additional File 1

1 Proof of Proposition 1

We start with a lemma on the properties of the modified Bessel function of the first kind of order $\nu := \alpha - 1$.

Lemma 1: For $\tilde{\alpha} + \nu > 0$ and $p > c$ it holds that

$$\int_0^\infty x^{\tilde{\alpha}-1} \exp(-px)\, I_\nu(cx)\, dx \;=\; p^{-(\tilde{\alpha}+\nu)} \left(\frac{c}{2}\right)^\nu \frac{\Gamma(\nu+\tilde{\alpha})}{\Gamma(\nu+1)}\; {}_2F_1\left(\frac{\nu+\tilde{\alpha}}{2}, \frac{\nu+\tilde{\alpha}+1}{2}, \nu+1, \frac{c^2}{p^2}\right),$$

where ${}_2F_1(\cdot)$ is the Gauss hypergeometric function (see [1], p. 350). For a formal proof of Lemma 1, see [2]. □

The proof of Proposition 1 is obtained by deriving the joint density function $f_{R,b}$ of the random variables $R := M + U$ and $b = M/(M+U) = M/R$. Transforming $(M,U) = (Rb, R(1-b))$ into (R,b) yields the Jacobian matrix

$$\tilde{J} = \begin{pmatrix} \frac{\partial Rb}{\partial R} & \frac{\partial Rb}{\partial b} \\ \frac{\partial R(1-b)}{\partial R} & \frac{\partial R(1-b)}{\partial b} \end{pmatrix} = \begin{pmatrix} b & R \\ (1-b) & -R \end{pmatrix} \tag{1}$$

with $|\det(\tilde{J})| = R$. It follows that, under the assumptions of Proposition 1,

$$\begin{aligned} f_{R,b}(r,b) \;&=\; \frac{(\lambda_m\lambda_u)^{\frac{\alpha+1}{2}}}{(1-\rho)\,\rho^{\frac{\alpha-1}{2}}\Gamma(\alpha)}\; r^\alpha \,(b\,(1-b))^{\frac{\alpha-1}{2}} \exp\left(-\frac{\lambda_m rb + \lambda_u r(1-b)}{1-\rho}\right) \\ &\quad \times I_{\alpha-1}\left(\frac{2\sqrt{\rho\,\lambda_m\lambda_u\, r^2\, b\,(1-b)}}{1-\rho}\right). \end{aligned} \tag{2}$$

Defining

$$Z(b) := \int r^\alpha \exp\left(-\frac{\lambda_m rb + \lambda_u r(1-b)}{1-\rho}\right) I_{\alpha-1}\left(\frac{2\sqrt{\rho\,\lambda_m\lambda_u\, r^2\, b\,(1-b)}}{1-\rho}\right) dr\,, \tag{3}$$

the marginal density function $f_b(b)$ is derived by integrating $f_{R,b}$ over R:

$$f_b(b) = \int f_{R,b}(r,b)\, dr = \frac{(\lambda_m\lambda_u)^{\frac{\alpha+1}{2}}}{(1-\rho)\,\rho^{\frac{\alpha-1}{2}}\,\Gamma(\alpha)}\,(b\,(1-b))^{\frac{\alpha-1}{2}}\, Z(b)\,. \tag{4}$$

Setting

$$\tilde{\alpha} = \alpha+1\,,\quad \nu = \alpha-1\,,\quad p = \frac{\lambda_m b + \lambda_u(1-b)}{1-\rho}\,,\quad c = \frac{2\sqrt{\rho\,\lambda_m\lambda_u\, b\,(1-b)}}{1-\rho} \tag{5}$$

and making use of the fact that

$${}_2F_1\,(\alpha,\delta,\alpha,x) = (1-x)^{-\delta}\,, \tag{6}$$

one obtains by application of Lemma 1 that

$$Z(b) = \frac{\Gamma(2\alpha)}{\Gamma(\alpha)}\,(1-\rho)^{\alpha+1} \frac{\left(\sqrt{\rho\,\lambda_m\lambda_u\, b\,(1-b)}\right)^{\alpha-1}}{(\lambda_m b + \lambda_u(1-b))^{2\alpha}} \left(1 - \frac{4\rho\,\lambda_m\lambda_u\, b\,(1-b)}{(\lambda_m b + \lambda_u(1-b))^2}\right)^{-\frac{2\alpha+1}{2}}\,. \tag{7}$$

Combining (4) and (7) yields the probability density function stated in Proposition 1. □

2 Proof of Proposition 2

Defining $\theta := \lambda_m/\lambda_u$, the log-likelihood function derived from of Equation (9) of the manuscript becomes

$$\begin{aligned}
\sum_{i=1}^{n} \log(f_b(b_i;\alpha,\rho,\theta)) &= \sum_{i=1}^{n} \Big[\log(\Gamma(2\alpha)) - 2\log(\Gamma(\alpha)) + \alpha\log(\lambda_m\lambda_u) + \alpha\log(1-\rho) \\
&\quad + (\alpha-1)\log(b_i(1-b_i)) + \log(\lambda_m b_i + \lambda_u(1-b_i)) \\
&\quad - (\alpha+0.5)\log\left((\lambda_m b_i + \lambda_u(1-b_i))^2 - 4\,\rho\,\lambda_m\lambda_u\, b_i(1-b_i)\right) \Big] \\
&= \sum_{i=1}^{n} \Big[\log(\Gamma(2\alpha)) - 2\log(\Gamma(\alpha)) + \alpha\log(\theta\lambda_u^2) + \alpha\log(1-\rho) \\
&\quad + (\alpha-1)\log(b_i(1-b_i)) + \log\left(\lambda_u\left((\theta-1)\,b_i+1\right)\right) \\
&\quad - (\alpha+0.5)\log\left((\lambda_u((\theta-1)\,b_i+1))^2 - 4\,\rho\,\theta\lambda_u^2\, b_i(1-b_i)\right) \Big] \\
&= \sum_{i=1}^{n} \Big[\log(\Gamma(2\alpha)) - 2\log(\Gamma(\alpha)) + \alpha\log(\theta) + \alpha\log(1-\rho) \\
&\quad + (\alpha-1)\log(b_i(1-b_i)) + \log\left((\theta-1)\,b_i+1\right) \\
&\quad - (\alpha+0.5)\log\left(((\theta-1)\,b_i+1)^2 - 4\,\rho\,\theta\, b_i(1-b_i)\right) \Big]. \qquad (8)
\end{aligned}$$

□

3 Derivation of the Observed Information Matrix

Defining $D_1 := (\exp(\boldsymbol{X}_i^T\gamma)-1)\,b_i+1$ and $D_2 := D_1^2 - 4\,\rho\exp(\boldsymbol{X}_i^T\gamma)\,b_i\,(1-b_i)$, the first derivative of Equation (13) of the manuscript w.r.t. γ is given by

$$\begin{aligned}
&\frac{\partial}{\partial\gamma}\sum_{i=1}^{n}\log(f_b(b_i,\boldsymbol{X}_i;\alpha,\rho,\gamma)) \\
&= \sum_{i=1}^{n}\Big[\alpha\boldsymbol{X}_i^T + \frac{\boldsymbol{X}_i^T b_i\exp(\boldsymbol{X}_i^T\gamma)}{D_1} - (\alpha+0.5)\,\frac{2\,(D_1 - 2\,\rho\,(1-b_i))\,\boldsymbol{X}_i^T b_i\exp(\boldsymbol{X}_i^T\gamma)}{D_2}\Big]. \qquad (9)
\end{aligned}$$

It follows that the observed information matrix is given by

$$\begin{aligned}
J(\alpha,\rho,\gamma) &= -\frac{\partial^2}{\partial^2\gamma}\sum_{i=1}^{n}\log(f_b(b_i,\boldsymbol{X}_i;\alpha,\rho,\gamma)) \qquad (10) \\
&= \sum_{i=1}^{n}\Big[\frac{\left(D_1 - b_i\exp(\boldsymbol{X}_i^T\gamma)\right)b_i\exp(\boldsymbol{X}_i^T\gamma)\boldsymbol{X}_i\boldsymbol{X}_i^T}{D_1^2} \\
&\quad - (\alpha+0.5)\,\frac{2\,b_i\exp(\boldsymbol{X}_i^T\gamma)\left(D_1 + b_i\exp(\boldsymbol{X}_i^T\gamma) - 2\,\rho\,(1-b_i)\right)\boldsymbol{X}_i\boldsymbol{X}_i^T}{D_2} \\
&\quad + (\alpha+0.5)\,\frac{4\,b_i^2\exp(2\boldsymbol{X}_i^\top\gamma)\,(D_1 - 2\rho(1-b_i))^2\,\boldsymbol{X}_i\boldsymbol{X}_i^T}{D_2^2}\Big]. \qquad (11)
\end{aligned}$$

References

[1] Nadarajah, S., Kotz, S.: Jensen's bivariate gamma distribution: Ratios of components. Journal of Statistical Computation and Simulation **77**, 349–358 (2007)

[2] Prudnikov, A.P., Brychkov, Y.A., Marichev, O.I.: Integrals and Series. Gordon and Breach Science Publishers, Amsterdam (1986)
