# Supplementary material for: A statistical model for the analysis of beta values in DNA methylation studies
Source: BMC Bioinformatics. 2016 Nov 22;17:480. doi: 10.1186/s12859-016-1347-4 (PMC5120494; doi:10.1186/s12859-016-1347-4)
Supplement: Additional file 3 — R package mboostDevel. (GZ 2038 kb) [file 12859_2016_1347_MOESM3_ESM.gz › mboostDevel/inst/doc/SurvivalEnsembles.pdf]

Survival Ensembles

Torsten Hothorn[1,⋆], Peter Bühlmann[2], Sandrine Dudoit[3],
Annette Molinaro[4] and Mark J. van der Laan[3]

[1]Institut für Statistik
Ludwig-Maximilians-Universität München
Ludwigstraße 33, D-80539 München, Germany
Tel: ++49–9131–8522707
Fax: ++49–9131–8525740
`Torsten.Hothorn@R-project.org`

[2]Seminar für Statistik, ETH Zürich, CH-8032 Zürich, Switzerland
`buhlmann@stat.math.ethz.ch`

[3]Division of Biostatistics, University of California, Berkeley
140 Earl Warren Hall, #7360, Berkeley, CA 94720-7360, USA
`sandrine@stat.Berkeley.EDU`
`laan@stat.Berkeley.EDU`

[4]Division of Biostatistics, Epidemiology and Public Health
Yale University School of Medicine, 206 LEPH
60 College Street PO Box 208034, New Haven CT 06520-8034
`annette.molinaro@yale.edu`

1 Illustrations and Applications

This document reproduces the data analyses presented in Hothorn et al. (2006). For a description of the theory behind applications shown here we refer to the original manuscript. The results differ slightly due to technical changes or bug-fixes in **mboost** that have been implemented after the paper was printed.

1.1 Acute myeloid leukemia

Data preprocessing Compute IPC weights, define risk score and set up learning sample:

```
R> ### compute IPC weights
R> AMLw <- IPCweights(Surv(clinical$time, clinical$event))
```

```
R> ### risk score
R> risk <- rep(0, nrow(clinical))
R> rlev <- levels(clinical[, "Cytogenetic.group"])
R> risk[clinical[, "Cytogenetic.group"] %in% rlev[c(7,8,4)]] <- "low"
R> risk[clinical[, "Cytogenetic.group"] %in% rlev[c(5, 9)]] <- "intermediate"
R> risk[clinical[, "Cytogenetic.group"] %in% rlev[-c(4,5, 7,8,9)]] <- "high"
R> risk <- as.factor(risk)
R> ### set-up learning sample
R> AMLlearn <- cbind(clinical[, c("time", "Sex", "Age", "LDH", "WBC",
                              "FLT3.aberration.", "MLL.PTD", "Tx.Group.")],
                 risk = risk,
                 iexpressions[, colnames(iexpressions) %in% selgenes[["Clone.ID"]]])
R> cc <- complete.cases(AMLlearn)
R> AMLlearn <- AMLlearn[AMLw > 0 & cc,]
R> AMLw <- AMLw[AMLw > 0 & cc]
```

Model fitting Fit random forest for censored data

```
R> ### controls for tree growing
R> ctrl <- cforest_control(mincriterion = 0.1, mtry = 5, minsplit = 5, ntree = 250)
R> ### fit random forest for censored data (warnings are OK here)
R> AMLrf <- cforest(I(log(time)) ~ ., data = AMLlearn, control = ctrl,
                  weights = AMLw)
```

and L_2Boosting for censored data

```
R> AMLl2b <- glmboost(I(log(time)) ~ ., data = AMLlearn, weights = AMLw,
                     control = boost_control(mstop = 5000))
```

Compute fitted values

```
R> ### restrict number of boosting iterations and inspect selected variables
R> AMLl2b <- AMLl2b[mstop(aic)]
R> cAML <- coef(AMLl2b)
R> cAML[abs(cAML) > 0]
```

```
    (Intercept)              Age              WBC
      0.5642932        0.0059785       -0.0056200
     MLL.PTDyes    Tx.Group.AUTO     Tx.Group.Ind
     -0.3153912        0.4542954       -2.1216104
  `IMAGE:145643`   `IMAGE:345601`   `IMAGE:377560`
      0.1062577        0.0043043        0.0275653
`IMAGE:2043415`  `IMAGE:1584563`   `IMAGE:347035`
      0.0550938       -0.0025929       -0.0084766
  `IMAGE:262695`    `IMAGE:26418`   `IMAGE:950479`
      0.0269555        0.0080214        0.0371741
`IMAGE:1534700`  `IMAGE:1472689`  `IMAGE:1526826`
      0.0283645        0.0225640       -0.0278373
  `IMAGE:786302`   `IMAGE:243614`   `IMAGE:417884`
      0.0449326       -0.0566722       -0.0248869
`IMAGE:1592006`   `IMAGE:884333`   `IMAGE:133273`
```

```
R> ### AIC criterion
R> plot(aic <- AIC(AMLl2b))
```

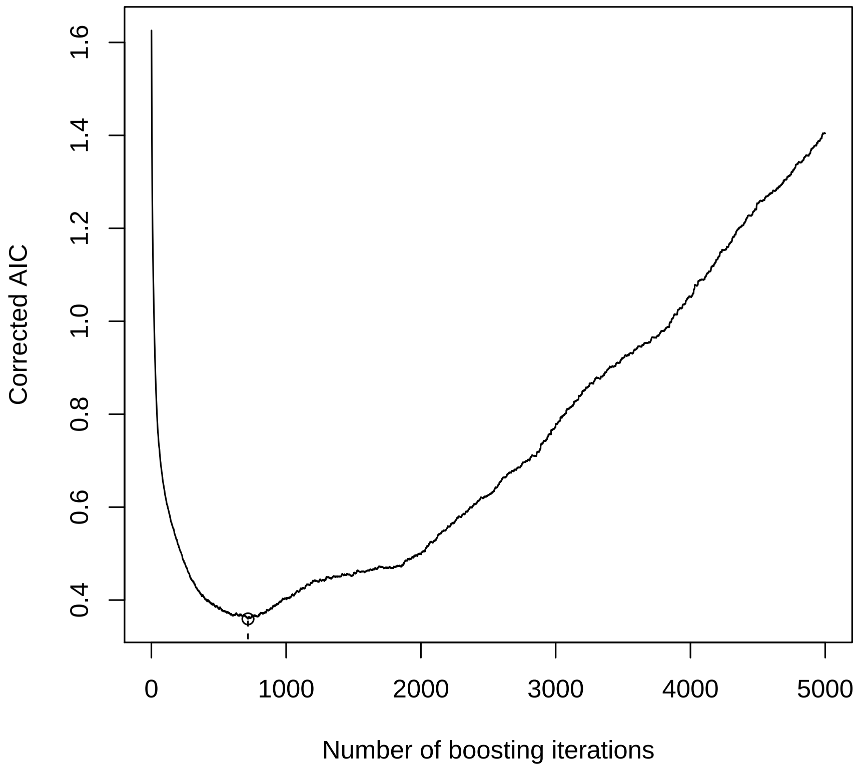

Figure 1: AIC criterion for AML data.

```
     -0.0355121       0.0128054       0.0257924
 `IMAGE:950888`  `IMAGE:809533`   `IMAGE:49389`
      0.0348510      -0.0583489       0.1210483
 `IMAGE:856174`  `IMAGE:435036`  `IMAGE:491751`
      0.0205370       0.0620215       0.1155506
 `IMAGE:782835`   `IMAGE:52930` `IMAGE:2545705`
     -0.1108508      -0.0245246      -0.0788422
 `IMAGE:756405`  `IMAGE:129032` `IMAGE:1610168`
      0.0085293      -0.1158217       0.0137998
  `IMAGE:69002` `IMAGE:2019101` `IMAGE:1456160`
     -0.2793326      -0.0966590      -0.1041466
`IMAGE:2566064`  `IMAGE:565083`  `IMAGE:843028`
      0.0154665       0.1875592       0.0698328
  `IMAGE:68794`  `IMAGE:488505`  `IMAGE:291756`
      0.0761390       0.2784632       0.0994879
 `IMAGE:810801` `IMAGE:1702742`  `IMAGE:380462`
      0.0465851      -0.0104549      -0.0957299
 `IMAGE:154472`  `IMAGE:302540`  `IMAGE:135221`
     -0.1454724       0.0188789      -0.0366827
`IMAGE:1567220`
      0.0485058
```

```
R> ### fitted values
R> AMLprf <- predict(AMLrf, newdata = AMLlearn)
R> AMLpb <- predict(AMLl2b, newdata = AMLlearn)
```

1.2 Node-positive breast cancer

Data preprocessing Compute IPC weights and set up learning sample:

```
R> ### attach data
R> data("GBSG2", package = "TH.data")
R> ### IPC weights
R> GBSG2w <- IPCweights(Surv(GBSG2$time, GBSG2$cens))
R> ### set-up learning sample
R> GBSG2learn <- cbind(GBSG2[,-which(names(GBSG2) %in% c("time", "cens"))],
                   ltime = log(GBSG2$time))
R> n <- nrow(GBSG2learn)
```

Model fitting

```
R> ### linear model
R> LMmod <- lm(ltime ~ . , data = GBSG2learn, weights = GBSG2w)
R> LMerisk <- sum((GBSG2learn$ltime - predict(LMmod))^2*GBSG2w) / n
R> ### regression tree
R> pos <- GBSG2w > 0
R> TRmod <- rpart(ltime ~ . , data = GBSG2learn, weights = GBSG2w,
                 subset = pos)
R> TRerisk <- sum((GBSG2learn$ltime[pos] - predict(TRmod))^2*GBSG2w[pos]) / n
R> ### tree controls
```

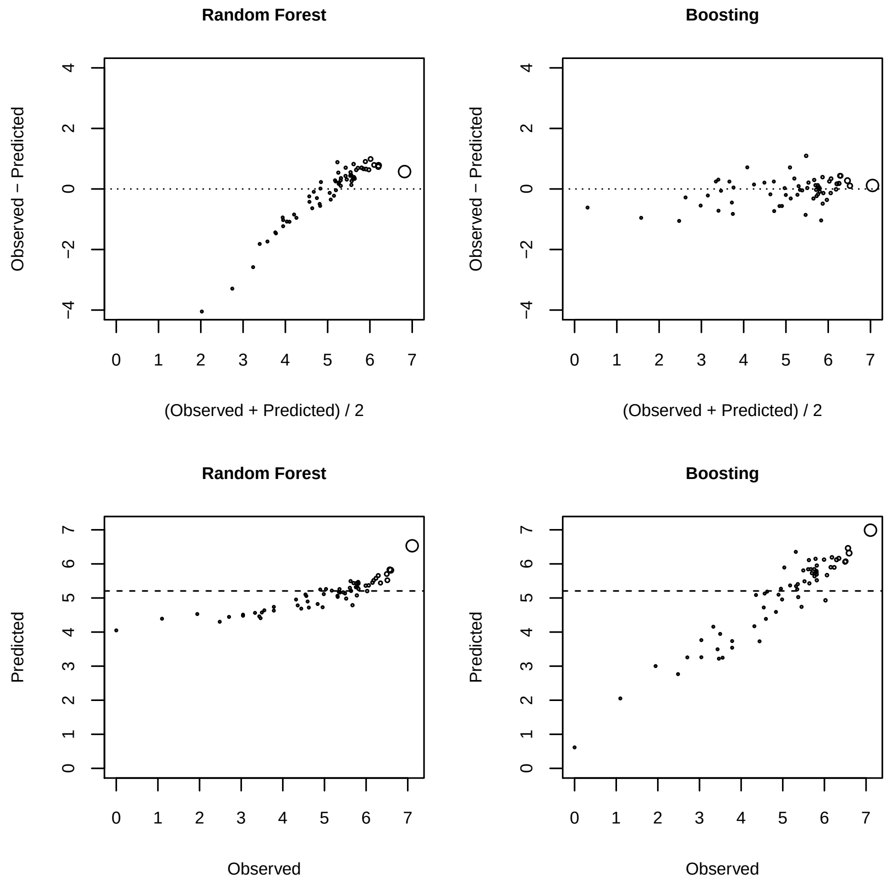

Figure 2: AML data: Reproduction of Figure 1.

```
R> ctrl <- cforest_control(mincriterion = qnorm(0.95), mtry = 5,
                          minsplit = 5, ntree = 100)
R> ### fit random forest for censored data (warnings are OK here)
R> RFmod <- cforest(ltime ~ . , data = GBSG2learn, weights = GBSG2w,
                     control = ctrl)
R> ### fit L2 boosting for censored data
R> L2Bmod <- glmboost(ltime ~ ., data = GBSG2learn, weights = GBSG2w,
                       control = boost_control(mstop = 250))
R> ### with Huber loss function
R> L2BHubermod <- glmboost(ltime ~ ., data = GBSG2learn, weights = GBSG2w,
                            family = Huber(d = log(2)))
```

Compute fitted values:

```
R> GBSG2Hp <- predict(L2BHubermod, newdata = GBSG2learn)
R> L2Berisk <- sum((GBSG2learn$ltime - predict(L2Bmod, newdata = GBSG2learn))^2*GBSG2w) / n
R> RFerisk <- sum((GBSG2learn$ltime - predict(RFmod, newdata = GBSG2learn))^2*GBSG2w) / n
```

```
R> plot(aic <- AIC(L2Bmod))
```

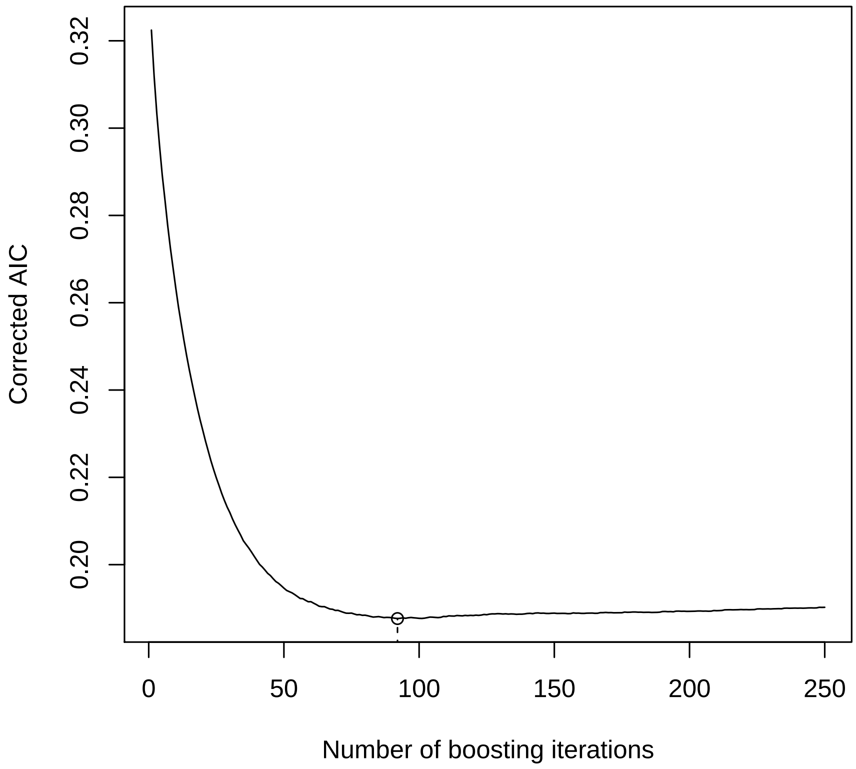

Figure 3: AIC criterion for GBSG2 data.

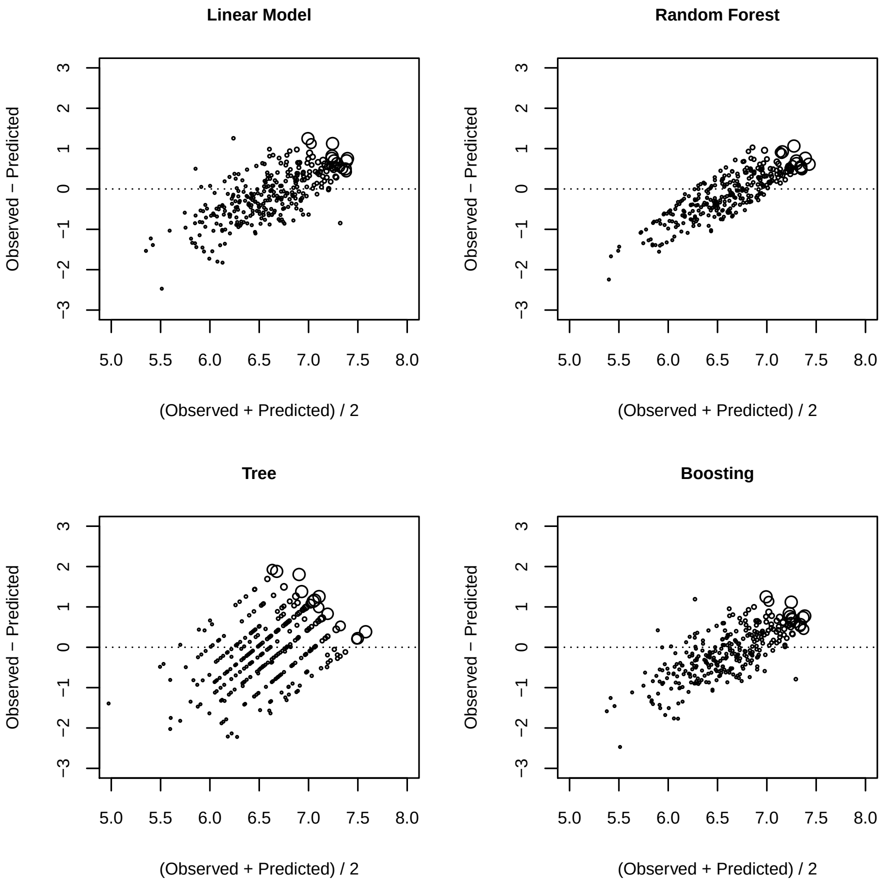

Figure 4: GBSG-2 data: Reproduction of Figure 3.

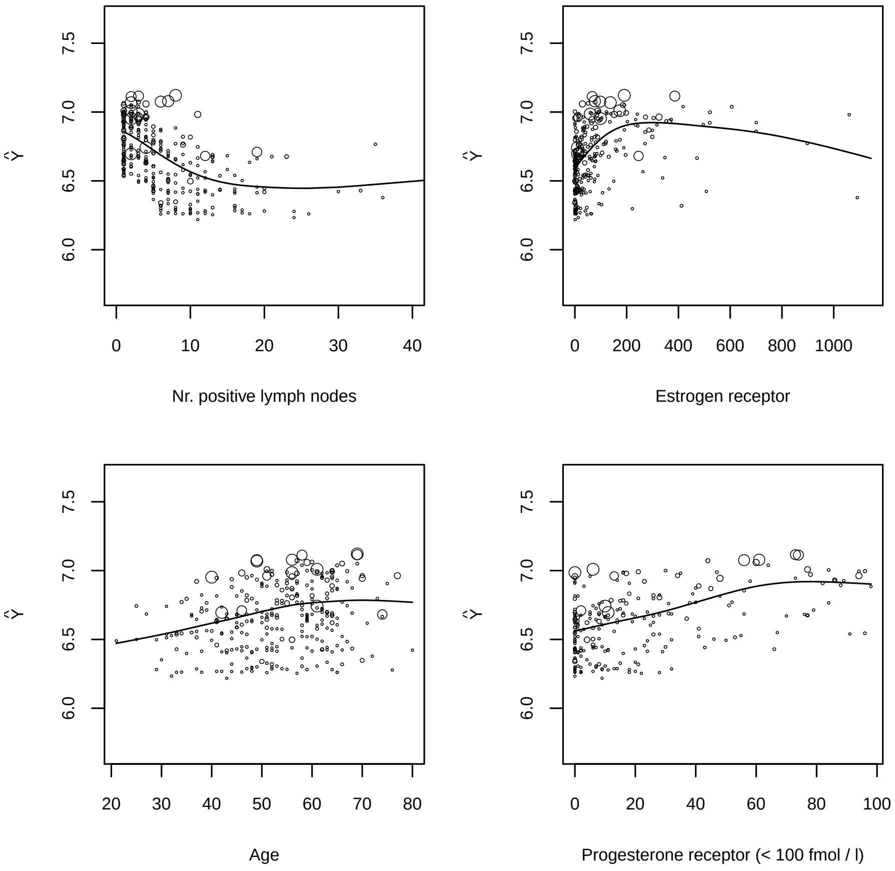

Figure 5: GBSG-2 data: Reproduction of Figure 5.

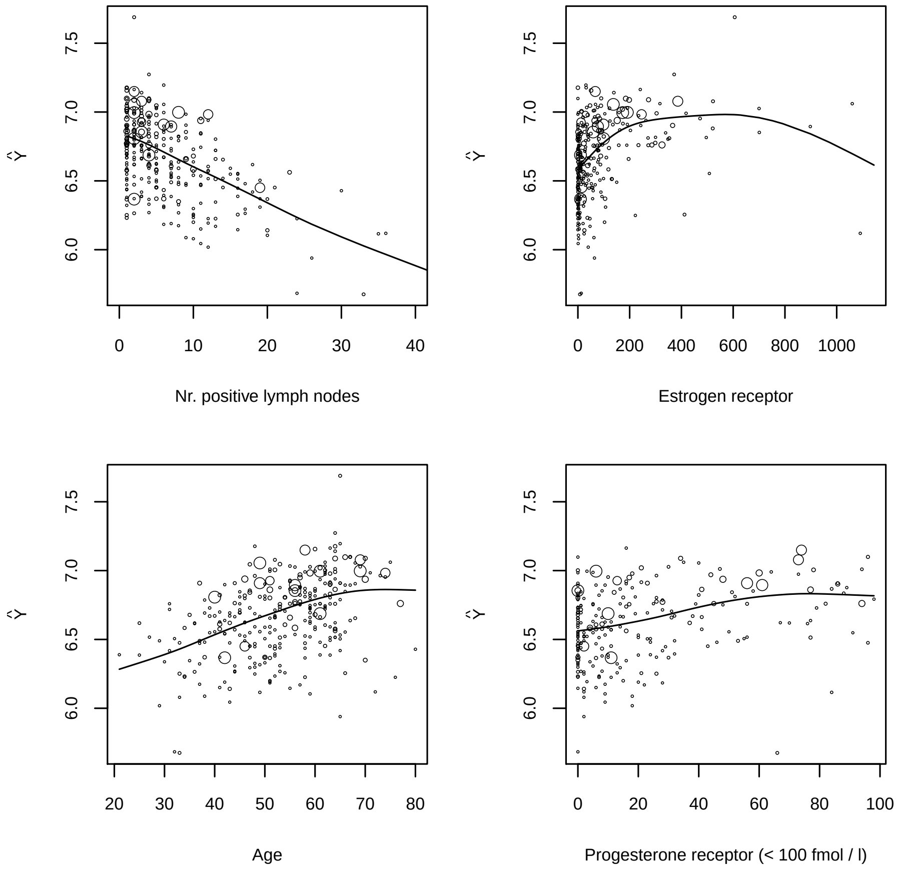

Figure 6: GBSG-2 data: Reproduction of Figure 6.

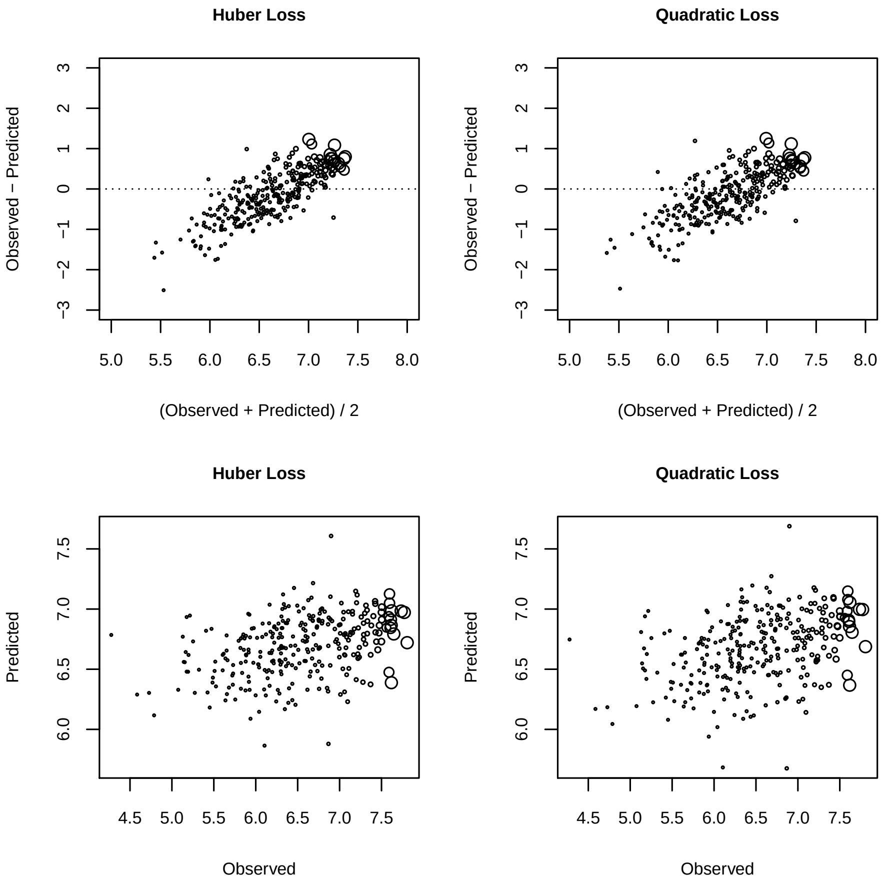

Figure 7: GBSG-2 data: Reproduction of Figure 7.

References

T. Hothorn, P. Bühlmann, S. Dudoit, A. Molinaro, and M. van der Laan. Survival ensembles. *Biostatistics*, 7:355–373, 2006.
