# Supplementary material for: A statistical model for the analysis of beta values in DNA methylation studies
Source: BMC Bioinformatics. 2016 Nov 22;17:480. doi: 10.1186/s12859-016-1347-4 (PMC5120494; doi:10.1186/s12859-016-1347-4)
Supplement: Additional file 3 — R package mboostDevel. (GZ 2038 kb) [file 12859_2016_1347_MOESM3_ESM.gz › mboostDevel/inst/doc/mboost.pdf]

# Model-based Boosting 2.0

**Torsten Hothorn** *LMU München* Torsten.Hothorn@R-project.org
**Peter Bühlmann** *ETH Zürich* buhlmann@stat.math.ethz.ch
**Thomas Kneib** *Universität Oldenburg* thomas.kneib@uni-oldenburg.de
**Matthias Schmid** *FAU Erlangen-Nürnberg* Matthias.Schmid@imbe.med.uni-erlangen.de
**Benjamin Hofner** *FAU Erlangen-Nürnberg* Benjamin.Hofner@imbe.med.uni-erlangen.de

**Editor:** Mikio Braun

**This is an extended version of the manuscript**
Torsten Hothorn, Peter Bühlmann, Thomas Kneib, Mattthias Schmid and Benjamin Hofner (2010), Model-based Boosting 2.0. *Journal of Machine Learning Research*, **11**, 2109 – 2113; URL http://jmlr.csail.mit.edu/papers/v11/hothorn10a.html.

## Abstract

This is an extended version of the manuscript Torsten Hothorn, Peter Bühlmann, Thomas Kneib, Mattthias Schmid and Benjamin Hofner (2010), Model-based Boosting 2.0. *Journal of Machine Learning Research*, **11**, 2109 – 2113; http://jmlr.csail.mit.edu/papers/v11/hothorn10a.html.

We describe version 2.0 of the R add-on package *mboost*. The package implements boosting for optimizing general risk functions utilizing component-wise (penalized) least squares estimates or regression trees as base-learners for fitting generalized linear, additive and interaction models to potentially high-dimensional data.

**Keywords:** component-wise functional gradient descent, splines, decision trees.

## 1. Overview

The R add-on package *mboost* (Hothorn et al., 2010) implements tools for fitting and evaluating a variety of regression and classification models that have been suggested in machine learning and statistics. Optimization within the empirical risk minimization framework is performed via a component-wise functional gradient descent algorithm. The algorithm originates from the statistical view on boosting algorithms (Friedman et al., 2000; Bühlmann and Yu, 2003). The theory and its implementation in *mboost* allow for fitting complex prediction models, taking potentially many interactions of features into account, as well as for fitting additive and linear models. The model class the package deals with is best described by so-called structured additive regression (STAR) models, where some characteristic $\xi$ of the conditional distribution of a response variable $Y$ given features $X$ is modeled through a regression function $f$ of the features $\xi(Y|X = x) = f(x)$. In order to facilitate parsimonious and interpretable models, the regression function $f$ is structured, i.e., restricted to additive functions $f(x) = \sum_{j=1}^{p} f_j(x)$. Each model component $f_j(x)$ might take only a subset of the features into account. Special cases are linear models $f(x) = x^\top\beta$, additive models $f(x) = \sum_{j=1}^{p} f_j(x^{(j)})$, where $f_j$ is a function of the $j$th feature $x^{(j)}$ only (smooth functions or stumps, for example) or a more complex function where $f(x)$ is implicitly defined as the sum of multiple decision trees including higher-order interactions. The latter case corresponds to boosting with trees. Combinations of these structures are also possible. The most important advantage of such a decomposition of the regression function is that each component of a fitted model can be looked at and interpreted separately for gaining a better understanding of the model at hand.

The characteristic $\xi$ of the distribution depends on the measurement scale of the response $Y$ and the scientific question to be answered. For binary or numeric variables, some function of the expectation may be appropriate, but also quantiles or expectiles may be interesting. The definition of $\xi$ is determined by defining a loss function $\rho$ whose empirical risk is to be minimized under some algorithmic constraints (i.e., limited number of boosting iterations). The model is then fitted using

$$(\hat{f}_1, \dots, \hat{f}_p) = \underset{(f_1,\dots,f_p)}{\operatorname{argmin}} \sum_{i=1}^{n} w_i \rho \left( y_i, \sum_{j=1}^{p} f_j(x) \right).$$

Here $(y_i, x_i), i = 1, \dots, n$, are $n$ training samples with responses $y_i$ and potentially high-dimensional feature vectors $x_i$, and $w_i$ are some weights. The component-wise boosting algorithm starts with some offset for $f$ and iteratively fits residuals defined by the negative gradient of the loss function evaluated at the current fit by updating only the best model component in each iteration. The details have been described by Bühlmann and Yu (2003). Early stopping via resampling approaches or AIC leads to sparse models in the sense that only a subset of important model components $f_j$ defines the final model. A more thorough introduction to boosting with applications in statistics based on version 1.0 of *mboost* is given by Bühlmann and Hothorn (2007).

As of version 2.0, the package allows for fitting models to binary, numeric, ordered and censored responses, i.e., regression of the mean, robust regression, classification (logistic and exponential loss), ordinal regression[1], quantile[1] and expectile[1] regression, censored regression (including Cox, Weibull[1], log-logistic[1] or lognormal[1] models) as well as Poisson and negative binomial regression[1] for count data can be performed. Because the structure of the regression function $f(x)$ can be chosen independently from the loss function $\rho$, interesting new models can be fitted (e.g., in geoadditive regression, Kneib et al., 2009).

## 2. Design and Implementation

The package incorporates an infrastructure for representing loss functions (so-called 'families'), base-learners defining the structure of the regression function and thus the model components $f_j$, and a generic implementation of component-wise functional gradient descent. The main progress in version 2.0 is that only one implementation of the boosting algorithm is applied to all possible models (linear, additive, tree-based) and all families. Earlier versions were based on three implementations, one for linear models, one for additive models, and one for tree-based boosting. In comparison to the 1.0 series, the reduced code basis is easier to maintain, more robust and regression tests have been set-up in a more unified way. Specifically, the new code basis results in an enhanced and more user-friendly formula interface. In addition, convenience functions for hyper-parameter selection, faster computation of predictions and improved visual model diagnostics are available.

Currently implemented base-learners include component-wise linear models (where only one variable is updated in each iteration of the algorithm), additive models with quadratic penalties (e.g., for fitting smooth functions via penalized splines, varying coefficients or bi- and trivariate tensor product splines, Schmid and Hothorn, 2008), and trees.

As a major improvement over the 1.0 series, computations on larger data sets (both with respect to the number of observations and the number of variables) are now facilitated by memory efficient implementations of the base-learners, mostly by applying sparse matrix techniques (package *Matrix*, Bates and Mächler, 2009) and parallelization for a cross-validation-based choice of the number of boosting iterations per default via package *parallel*. A more elaborate description of *mboost* 2.0 features is available from the `mboost` vignette[2].

1. Model family is new in version 2.0 or was added after the release of *mboost* 1.0.
2. Accessible via `vignette("mboost", package = "mboostDevel")`.

## 3. User Interface by Example

We illustrate the main components of the user-interface by a small example on human body fat composition: Garcia et al. (2005) used a linear model for predicting body fat content by means of common anthropometric measurements that were obtained for $n = 71$ healthy German women. In addition, the women's body composition was measured by Dual Energy X-Ray Absorptiometry (DXA). The aim is to describe the DXA measurements as a function of the anthropometric features. Here, we extend the linear model by i) an intrinsic variable selection via early stopping, ii) additional terms allowing for smooth deviations from linearity where necessary (by means of penalized splines orthogonalized to the linear effect, Kneib et al., 2009), iii) a possible interaction between two variables with known impact on body fat composition (hip and waist circumference) and iv) utilizing a robust median regression approach instead of $L_2$ risk. For the data (available as data frame `bodyfat`), the model structure is specified via a formula involving the base-learners corresponding to the different model components (linear terms: `bols()`; smooth terms: `bbs()`; interactions: `btree()`). The loss function (here, the check function for the 0.5 quantile) along with its negative gradient function are defined by the `QuantReg(0.5)` family (Fenske et al., 2009). The model structure (specified using the formula `fm`), the data and the family are then passed to function `mboost()` for model fitting:

```
R> library("mboostDevel")                ### attach package 'mboost'
R> print(fm)                        ### model structure

DEXfat ~ bols(age) + bols(waistcirc) + bols(hipcirc) + bols(elbowbreadth) +
    bols(kneebreadth) + bols(anthro3a) + bols(anthro3b) + bols(anthro3c) +
    bols(anthro4) + bbs(age, center = TRUE, df = 1) + bbs(waistcirc,
    center = TRUE, df = 1) + bbs(hipcirc, center = TRUE, df = 1) +
    bbs(elbowbreadth, center = TRUE, df = 1) + bbs(kneebreadth,
    center = TRUE, df = 1) + bbs(anthro3a, center = TRUE, df = 1) +
    bbs(anthro3b, center = TRUE, df = 1) + bbs(anthro3c, center = TRUE,
    df = 1) + bbs(anthro4, center = TRUE, df = 1) + btree(hipcirc,
    waistcirc, tree_controls = ctree_control(maxdepth = 2, mincriterion = 0))

R> ### fit model for conditional median of DEXfat
R> model <- mboost(fm,                          ### model structure
+                 data = bodyfat,               ### 71 observations
+                 family = QuantReg(tau = 0.5)) ### median regression
```

Once the model has been fitted it is important to assess the appropriate number of boosting iterations via the out-of-sample empirical risk. By default, 25 bootstrap samples from the training data are drawn and the out-of-bag empirical risk is computed (parallel computation if possible):

```
R> ### bootstrap for assessing the 'optimal' number of boosting iterations
R> cvm <- cvrisk(model, grid = 1:100 * 10)
R> model[mstop(cvm)]                ### restrict model to optimal mstop(cvm) iterations
```

Now, the final model is ready for a visual inspection:

```
R> plot(cvm); plot(model)           ### depict out-of bag risk & selected components
```

The resulting plots are given in Figure 1. They indicate that a model based on three components, including a smooth function of `anthro3b` and a bivariate function of hip and waist circumference, provides the best characterization of the median body fat composition (given the model specification offered to the boosting algorithm). A hip circumference larger than 110 cm leads to increased body fat but only if the waist circumference is larger than 90 cm.

The sources of the *mboost* package are distributed at the Comprehensive R Archive Network under GPL-2, along with binaries for all major platforms as well as documentation and regression tests. Development versions are available from `http://R-forge.R-project.org`.

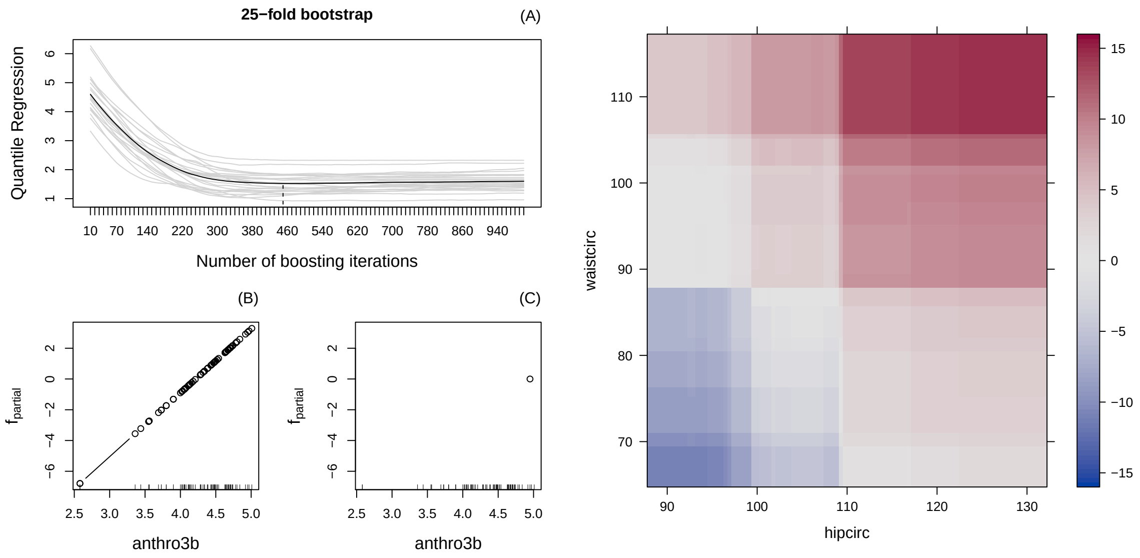


Figure 1: Out-of-bag empirical risk (A) indicating that 450 iterations are appropriate. Fitted model components for variable `anthro3b`, consisting of a linear (B) and smooth term (C). The right panel shows the interaction model component between hip and waist circumferences.

## 4. Overview on 2.0 Series Features

This additional section gives some further details on new features in the *mboost* 2.0 series. First of all, new families were included to permit quantile and expectile regression (`QuantReg()`, `ExpectReg()`), ordinal regression (`PropOdds()`), censored regression (`Weibull()`, `Loglog()`, `Lognormal()`) and count data regression (`NBinomial()`). All new families, except the quantile and expectile family, have an additional scale parameter, which is included in the family and is then subsequently estimated in the boosting algorithm without further need of modifications thereof.

For increased usability of *mboost*, some changes in the user interface of the base-learners were necessary. Most prominently, the `center` argument in `bols()` was renamed to `intercept` to reflect that it is used to specify whether the base-learner should contain an intercept (`intercept = TRUE`) or not. The argument `by` (formerly denoted by `z` in *mboost* 1.0), which can be used to specify varying coefficients, is now able to handle factors with more than two levels in addition to binary and continuous covariates. Furthermore, the base-learners `bss()` (smoothing spline) and `bns()` (penalized natural splines) are deprecated and replaced by `bbs()` (penalized splines), which results in qualitatively the same models but is computationally much more attractive.

The speed of model estimation and prediction has been notably improved in version 2.0. Regarding large data sets, a newly added search for duplicated observations of covariates in each base-learner leads to enormous speed-ups and far less memory consumption in the base-learner fitting in each boosting iteration. The effective number of observations in a weighted penalized least-squares problem is the number of unique covariate observations which is usually considerably smaller than the number of observations in the learning sample, especially for factors or smooth effects for millions of observations. The price to pay is an increased pre-processing time, however, pre-processing is just done once at the beginning of the algorithm.

In addition, version 2.0 of *mboost* offers an improved infrastructure: Firstly, the subset method `model[i]` can now be used to *enhance* or restrict a given boosting model to the specified boosting

iteration `i`. Note that in both cases the *original* `model` will be changed to reduce the memory footprint. If the boosting model is enhanced by specifying an index that is larger than the initial `mstop`, only the missing `i - mstop` steps are fitted. If the model is restricted, the spare steps are not dropped, but only hidden from the user, i.e., if we increase `i` again, these boosting steps are immediately available. The newly introduced function `selected()` allows to inspect the path of selected base-learners and is the basis for stability selection (in function `stabsel()`, cf. Meinshausen and Bühlmann, 2010). Secondly, an interface for parallel computing (based on *parallel*) is automatically used (if available) for cross-validation-based stopping of the algorithm in `cvrisk()`. With some extra work, other parallelization packages such as *snow* (Tierney et al., 2008) can immediately be integrated. Moreover, `cvrisk()` allows for more flexible definitions of resampling schemes ($k$-fold, cross-validation, bootstrap, subsampling). Thirdly, the `predict()`, `plot()` and `coef()` functions were enhanced. For linear models, the intercept is now adjusted for centered covariates and non-zero offsets are added to the intercept when using `coef(..., off2int = TRUE)`. Predictions are computed much faster now and for all three functions an argument `which` was included to allow to specify which base-learner(s) should be used for prediction and plotting or which coefficients should be extracted. Per default all *selected* base-learners are used. Users can specify `which` as numeric value(s) or as a (vector of) character string(s):

```
R> ### new data on a grid on range(anthro3b)
R> nd <- with(bodyfat, data.frame(anthro3b = seq(min(anthro3b), max(anthro3b),
+                                                 length = 100)))
R> ### predictions for all base-learners of 'anthro3b'
R> pr <- predict(model, which = "anthro3b", newdata = nd)
R> pr <- rowSums(pr)    ### aggregate linear and smooth effect
```

The combined effect can now be used for plotting (see Figure 2).

## References


Douglas Bates and Martin Mächler. *Matrix: Sparse and Dense Matrix Classes and Methods*, 2009. URL http://CRAN.R-project.org/package=Matrix. R package version 0.999375-38.

Peter Bühlmann and Torsten Hothorn. Boosting algorithms: Regularization, prediction and model fitting. *Statistical Science*, 22(4):477–505, 2007. doi: 10.1214/07-STS242.

Peter Bühlmann and Bin Yu. Boosting with the $L_2$ loss: Regression and classification. *Journal of the American Statistical Association*, 98:324–339, 2003.

Nora Fenske, Thomas Kneib, and Torsten Hothorn. Identifying risk factors for severe childhood malnutrition by boosting additive quantile regression. Technical report, Institut für Statistik, Ludwig-Maximilians-Universität München, 2009. URL http://epub.ub.uni-muenchen.de/10510/.

Jerome H. Friedman, Trevor Hastie, and Robert Tibshirani. Additive logistic regression: a statistical view of boosting (with discussion). *The Annals of Statistics*, 28:337–407, 2000.

A. L. Garcia, K. Wagner, T. Hothorn, C. Koebnick, H. J. Zunft, and U. Trippo. Improved prediction of body fat by measuring skinfold thickness, circumferences, and bone breadths. *Obesity Research*, 13(3):626–634, 2005.

Torsten Hothorn, Peter Bühlmann, Thomas Kneib, Matthias Schmid, and Benjamin Hofner. *mboost: Model-Based Boosting*, 2010. URL http://CRAN.R-project.org/package=mboost. R package version 2.0-4.

Thomas Kneib, Torsten Hothorn, and Gerhard Tutz. Variable selection and model choice in geoadditive regression models. *Biometrics*, 65(2):626–634, 2009.

```
R> plot(nd$anthro3b, pr, type = "l", xlab = "anthro3b",
+      ylab = "f(anthro3b)")
R> lines(nd$anthro3b, predict(model, which = "bols(anthro3b", newdata = nd),
+        type = "l", lty = "dashed")
```

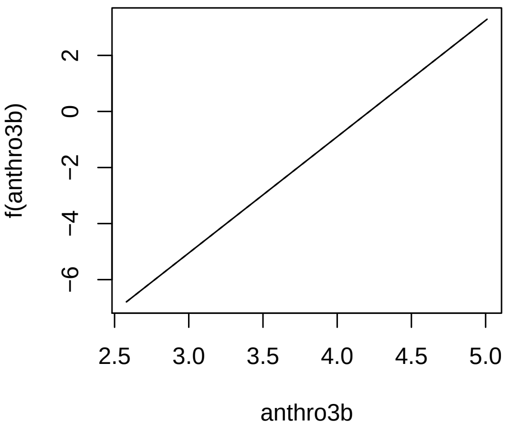


Figure 2: Effect of `anthro3b`, i.e., the combination of the linear and smooth effect (solid line) and for comparison solely the linear effect (dashed line).

Nicolai Meinshausen and Peter Bühlmann. Stability selection (with discussion). *Journal of the Royal Statistical Society, Series B*, 2010. In press.

Matthias Schmid and Torsten Hothorn. Boosting additive models using component-wise P-splines as base-learners. *Computational Statistics & Data Analysis*, 53(2):298–311, 2008.

Luke Tierney, Anthony J. Rossini, Na Li, and Hana Sevcikova. *snow: Simple Network of Workstations*, 2008. URL http://CRAN.R-project.org/package=snow. R package version 0.3-3.
