# Supplementary material for: A statistical model for the analysis of beta values in DNA methylation studies
Source: BMC Bioinformatics. 2016 Nov 22;17:480. doi: 10.1186/s12859-016-1347-4 (PMC5120494; doi:10.1186/s12859-016-1347-4)
Supplement: Additional file 3 — R package mboostDevel. (GZ 2038 kb) [file 12859_2016_1347_MOESM3_ESM.gz › mboostDevel/inst/doc/mboost_illustrations.pdf]

mboost Illustrations

Torsten Hothorn[1] and Peter Bühlmann[2]

[1] Institut für Statistik
Ludwig-Maximilians-Universität München
Ludwigstraße 33, D-80539 München, Germany
`Torsten.Hothorn@R-project.org`

[2] Seminar für Statistik
ETH Zürich, CH-8092 Zürich, Switzerland
`buhlmann@stat.math.ethz.ch`

1 Illustrations

This document reproduces the data analyses presented in Bühlmann and Hothorn (2007). For a description of the theory behind applications shown here we refer to the original manuscript. The results differ slightly due to technical changes or bugfixes in **mboost** that have been implemented after the paper was printed. Most important, `gamboost` uses penalized B-splines instead of smoothing splines as baselearners. The computations are much faster and the results differ only slightly (Schmid and Hothorn, 2008).

Illustration: Prediction of total body fat Garcia et al. (2005) report on the development of predictive regression equations for body fat content by means of $p = 9$ common anthropometric measurements which were obtained for $n = 71$ healthy German women. In addition, the women's body composition was measured by Dual Energy X-Ray Absorptiometry (DXA). This reference method is very accurate in measuring body fat but finds little applicability in practical environments, mainly because of high costs and the methodological efforts needed. Therefore, a simple regression equation for predicting DXA measurements of body fat is of special interest for the practitioner. Backward-elimination was applied to select important variables from the available anthropometrical measurements and Garcia et al. (2005) report a final linear model utilizing hip circumference, knee breadth and a compound covariate which is defined as the sum of log chin skinfold, log triceps skinfold and log subscapular skinfold:

```
R> bf_lm <- lm(DEXfat ~ hipcirc + kneebreadth + anthro3a, data = bodyfat)
R> coef(bf_lm)
```

```
(Intercept)     hipcirc kneebreadth    anthro3a 
  -75.23478     0.51153     1.90199     8.90964 
```

A simple regression formula which is easy to communicate, such as a linear combination of only a few covariates, is of special interest in this application: we employ the `glmboost` function from package **mboost** to fit a linear regression model by means of L_2Boosting with componentwise linear least squares. By default, the function `glmboost` fits a linear model (with initial $m_{\text{stop}} = 100$ and shrinkage parameter $\nu = 0.1$) by minimizing squared error (argument `family = Gaussian()` is the default):

```
R> bf_glm <- glmboost(DEXfat ~ ., data = bodyfat, center = TRUE)
```

Note that, by default, the mean of the response variable is used as an offset in the first step of the boosting algorithm. We center the covariates prior to model fitting in addition. As mentioned above, the special form of the base learner, i.e., componentwise linear least squares, allows for a reformulation of the boosting fit in terms of a linear combination of the covariates which can be assessed via

```
R> coef(bf_glm)
```

```
 (Intercept)          age    waistcirc      hipcirc 
  -98.816608     0.013602     0.189716     0.351626 
elbowbreadth  kneebreadth     anthro3a     anthro3b 
   -0.384140     1.736589     3.326860     3.656524 
    anthro3c 
    0.595363 
attr(,"offset")
[1] 30.783
```

We notice that most covariates have been used for fitting and thus no extensive variable selection was performed in the above model. Thus, we need to investigate how many boosting iterations are appropriate. Resampling methods such as cross-validation or the bootstrap can be used to estimate the out-of-sample error for a varying number of boosting iterations. The out-of-bootstrap mean squared error for 100 bootstrap samples is depicted in the upper part of Figure 1. The plot leads to the impression that approximately $m_{\text{stop}} = 44$ would be a sufficient number of boosting iterations. In Section **??**, a corrected version of the Akaike information criterion (AIC) is proposed for determining the optimal number of boosting iterations. This criterion attains its minimum for

```
R> mstop(aic <- AIC(bf_glm))
```

```
[1] 45
```

boosting iterations, see the bottom part of Figure 1 in addition. The coefficients of the linear model with $m_{\text{stop}} = 45$ boosting iterations are

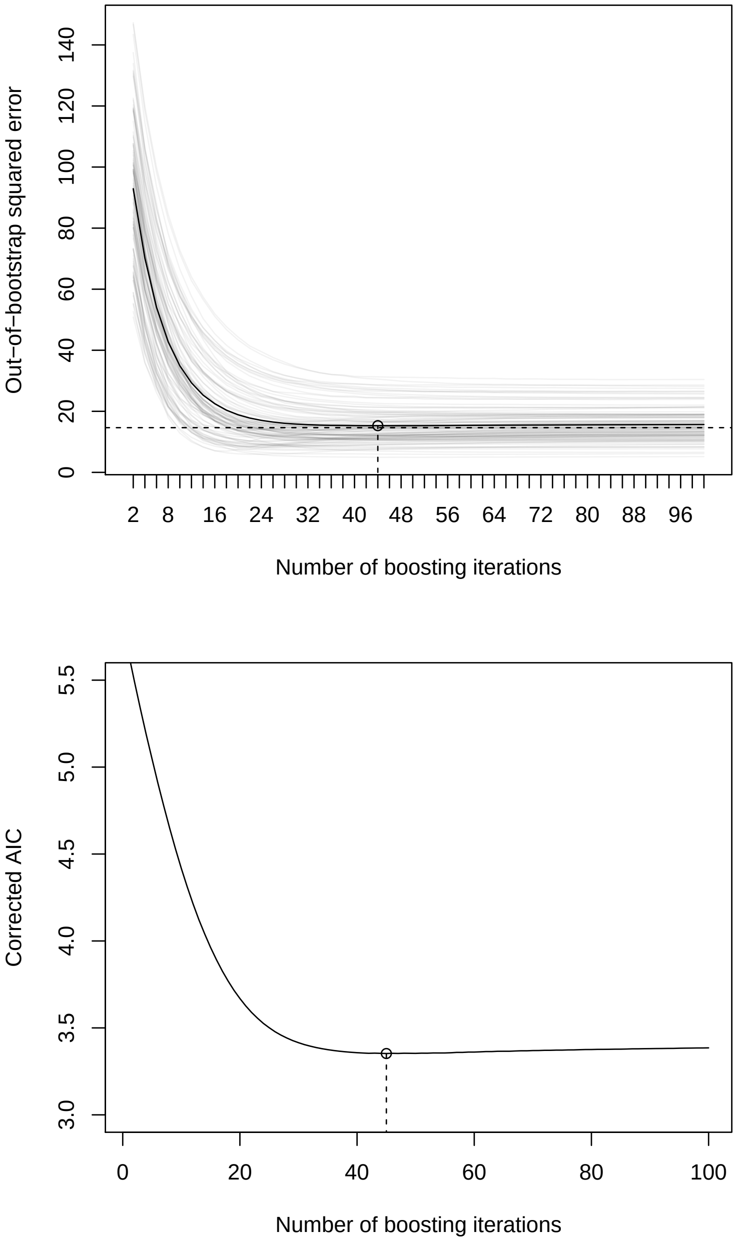

Figure 1: `bodyfat` data: Out-of-bootstrap squared error for varying number of boosting iterations m_{stop} (top). The dashed horizontal line depicts the average out-of-bootstrap error of the linear model for the pre-selected variables `hipcirc`, `kneebreadth` and `anthro3a` fitted via ordinary least squares. The lower part shows the corrected AIC criterion.

```
R> coef(bf_glm[mstop(aic)])
```

```
(Intercept)          age   waistcirc     hipcirc kneebreadth
-97.8458288   0.0023271   0.1893046   0.3488781   1.5217686
   anthro3a    anthro3b    anthro3c
  3.3268603   3.6051548   0.5043133
attr(,"offset")
[1] 30.783
```

and thus 8 covariates have been selected for the final model (intercept equal to zero occurs here for mean centered response and predictors and hence, $n^{-1}\sum_{i=1}^{n} Y_i = 30.783$ is the intercept in the uncentered model). Note that the variables `hipcirc`, `kneebreadth` and `anthro3a`, which we have used for fitting a linear model at the beginning of this paragraph, have been selected by the boosting algorithm as well.

Illustration: Prediction of total body fat (cont.) Being more flexible than the linear model which we fitted to the `bodyfat` data in Section **??**, we estimate an additive model using the `gamboost` function from **mboost** (first with pre-specified $m_{\text{stop}} = 100$ boosting iterations, $\nu = 0.1$ and squared error loss):

```
R> bf_gam <- gamboost(DEXfat ~ ., data = bodyfat, baselearner = "bss")
```

The degrees of freedom in the componentwise smoothing spline base procedure can be defined by the `dfbase` argument, defaulting to 4.

We can estimate the number of boosting iterations m_{stop} using the corrected AIC criterion described in Section **??** via

```
R> mstop(aic <- AIC(bf_gam))
```

```
[1] 51
```

Similar to the linear regression model, the partial contributions of the covariates can be extracted from the boosting fit. For the most important variables, the partial fits are given in Figure 2 showing some slight non-linearity, mainly for `kneebreadth`.

Illustration: Prediction of total body fat (cont.) Such transformations and estimation of a corresponding linear model can be done with the `glmboost` function, where the model formula performs the computations of all transformations by means of the `bs` (B-spline basis) function from the package **splines**. First, we set up a formula transforming each covariate

```
R> bsfm
```

```
DEXfat ~ bs(age) + bs(waistcirc) + bs(hipcirc) + bs(elbowbreadth) +
    bs(kneebreadth) + bs(anthro3a) + bs(anthro3b) + bs(anthro3c) +
    bs(anthro4)
```

and then fit the complex linear model by using the `glmboost` function with initial $m_{\text{stop}} = 5000$ boosting iterations:

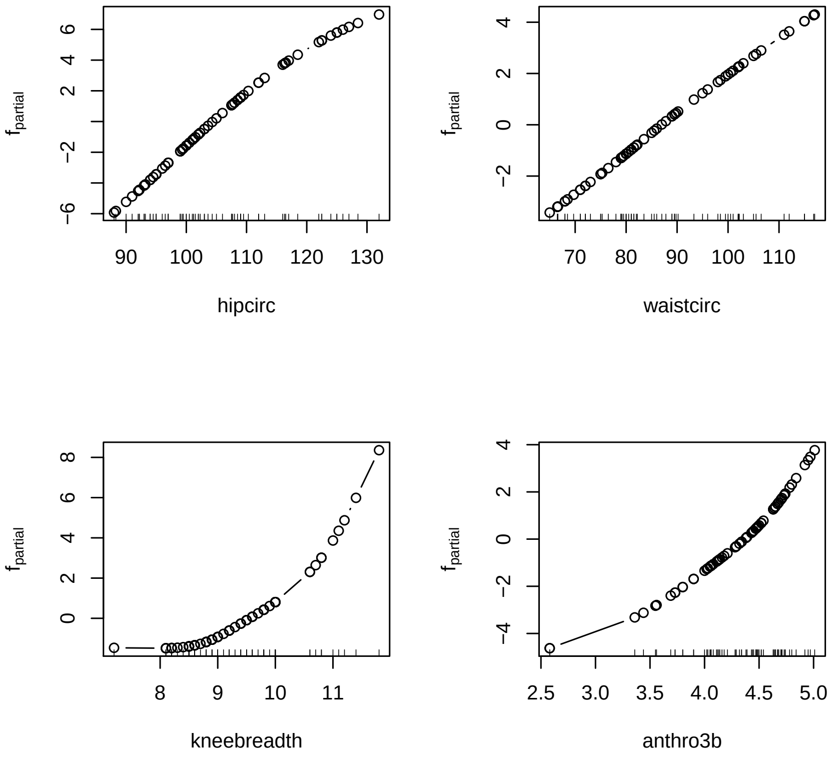

Figure 2: `bodyfat` data: Partial contributions of four covariates in an additive model (without centering of estimated functions to mean zero).

```
R> ctrl <- boost_control(mstop = 5000)
R> bf_bs <- glmboost(bsfm, data = bodyfat, control = ctrl)
R> mstop(aic <- AIC(bf_bs))
```

```
[1] 136
```

The corrected AIC criterion (see Section **??**) suggests to stop after $m_{\mathrm{stop}} =$ 136 boosting iterations and the final model selects 17 (transformed) predictor variables. Again, the partial contributions of each of the 9 original covariates can be computed easily and are shown in Figure 3 (for the same variables as in Figure 2). Note that the depicted functional relationship derived from the model fitted above (Figure 3) is qualitatively the same as the one derived from the additive model (Figure 2).

Illustration: Breast cancer subtypes Variable selection is especially important in high-dimensional situations. As an example, we study a binary classification problem involving $p = 7129$ gene expression levels in $n = 49$ breast cancer tumor samples (data taken from West et al., 2001). For each sample, a binary response variable describes the lymph node status (25 negative and 24 positive).

The data are stored in form of an *exprSet* object `westbc` (see Gentleman et al., 2004) and we first extract the matrix of expression levels and the response variable:

```
R> ### extract matrix of expression levels and binary response
R> x <- t(exprs(westbc))
R> y <- pData(westbc)$nodal.y
```

We aim at using L_2Boosting for classification, see Section **??**, with classical AIC based on the binomial log-likelihood for stopping the boosting iterations. Thus, we first transform the factor `y` to a numeric variable with 0/1 coding:

```
R> ### numeric 0/1 response variable
R> yfit <- as.numeric(y) - 1
```

The general framework implemented in **mboost** allows us to specify the negative gradient (the `ngradient` argument) corresponding to the surrogate loss function, here the squared error loss implemented as a function `rho`, and a different evaluating loss function (the `loss` argument), here the negative binomial log-likelihood, with the `Family` function as follows:

```
R> ### L2 boosting for classification with response in 0/1
R> ### and binomial log-likelihood as loss function
R> ### ATTENTION: use offset = 1/2 instead of 0!!!
R> rho <- function(y, f, w = 1) {
         p <- pmax(pmin(1 - 1e-5, f), 1e-5)
         -y * log(p) - (1 - y) * log(1 - p)
     }
R> ngradient <- function(y, f, w = 1) y - f
R> offset <- function(y, w) weighted.mean(y, w)
R> L2fm <- Family(ngradient = ngradient,
                  loss = rho, offset = offset)
```

The resulting object (called `L2fm`), bundling the negative gradient, the loss function and a function for computing an offset term (`offset`), can now be passed to the `glmboost` function for boosting with componentwise linear least squares (here initial $m_{\text{stop}} = 200$ iterations are used):

```
R> ### fit a linear model with initial mstop = 200 boosting iterations
R> ctrl <- boost_control(mstop = 200)
R> west_glm <- glmboost(x, yfit, family = L2fm, center = TRUE,
                        control = ctrl)
```

Fitting such a linear model to $p = 7129$ covariates for $n = 49$ observations takes about 0.5 seconds on a medium scale desktop computer (Intel Pentium 4, 2.8GHz). Thus, this form of estimation and variable selection is computationally very efficient.

The question how to choose m_{stop} can be addressed by the classical AIC criterion as follows

```
R> ### evaluate AIC based on binomial log-likelihood for _all_ boosting
R> ### iterations m = 1, ..., mstop = 200
R> aic <- AIC(west_glm, method = "classical")
R> ### where should one stop? mstop = 108 or 107
R> mstop(aic)
```

```
[1] 100
```

where the AIC is computed as -2(log-likelihood) + 2(degrees of freedom) = 2 (evaluating loss) + 2(degrees of freedom), see Formula (**??**). The notion of degrees of freedom is discussed in Section **??**.

Figure 4 shows the AIC curve depending on the number of boosting iterations. When we stop after $m_{\text{stop}} = 100$ boosting iterations, we obtain 33 genes with non-zero regression coefficients whose standardized values $\hat{\beta}^{(j)}\sqrt{\widehat{\text{Var}}(X^{(j)})}$ are depicted in the left panel of Figure 4.

Of course, we could also use BinomialBoosting for analyzing the data: the computational CPU time would be of the same order of magnitude, i.e., only a few seconds.

Illustration: Wisconsin prognostic breast cancer Prediction models for recurrence events in breast cancer patients based on covariates which have been computed from a digitized image of a fine needle aspirate of breast tissue (those measurements describe characteristics of the cell nuclei present in the image) have been studied by Street et al. (1995) (the data is part of the UCI repository Blake and Merz, 1998).

We first analyze this data as a binary prediction problem (recurrence vs. non-recurrence) and later in Section **??** by means of survival models. We are faced with many covariates ($p = 32$) for a limited number of observations without missing values ($n = 194$), and variable selection is an important issue. We can choose a classical logistic regression model via AIC in a stepwise algorithm as follows

```
R> ### remove missing values and time variable
R> cc <- complete.cases(wpbc)
R> wpbc2 <- wpbc[cc, colnames(wpbc) != "time"]
R> ### fit logistic regression model
R> wpbc_step <- step(glm(status ~ ., data = wpbc2, family = binomial()), trace = 0)
```

The final model consists of 16 parameters with

```
R> logLik(wpbc_step)
```

```
'log Lik.' -80.13 (df=16)
```

```
R> AIC(wpbc_step)
```

```
[1] 192.26
```

and we want to compare this model to a logistic regression model fitted via gradient boosting. We simply select the `Binomial` family (with default offset of $1/2 \log(\hat{p}/(1-\hat{p}))$, where $\hat{p}$ is the empirical proportion of recurrences) and we initially use $m_{\text{stop}} = 500$ boosting iterations

```
R> ### fit logistic regression model via gradient boosting
R> ctrl <- boost_control(mstop = 500)
R> wpbc_glm <- glmboost(status ~ ., data = wpbc2, family = Binomial(),
                        center = TRUE, control = ctrl)
```

The classical AIC criterion (-2 log-likelihood + 2 df) suggests to stop after

```
R> aic <- AIC(wpbc_glm, "classical")
R> aic
```

```
[1] 198.44
Optimal number of boosting iterations: 260
Degrees of freedom (for mstop = 260): 7.0319
```

boosting iterations. We now restrict the number of boosting iterations to $m_{\text{stop}} = 260$ and then obtain the estimated coefficients via

```
R> ### fit with new mstop
R> wpbc_glm <- wpbc_glm[mstop(aic)]
R> coef(wpbc_glm)[abs(coef(wpbc_glm)) > 0]
```

```
    (Intercept)      mean_texture    mean_symmetry
    2.3013e-01       -2.4215e-02      -3.3878e+00
 mean_fractaldim        SE_texture     SE_perimeter
    -2.0321e+01       -2.6603e-02       4.0908e-02
  SE_compactness      SE_concavity SE_concavepoints
    7.0280e+00        -4.6303e+00      -1.5737e+01
    SE_symmetry      worst_radius  worst_perimeter
    2.8601e+00         1.7777e-02       1.2639e-03
     worst_area worst_smoothness            tsize
    1.5854e-04         8.8372e+00       3.1014e-02
         pnodes
    2.5981e-02
```

(because of using the offset-value $\hat{f}^{[0]}$, we have to add the value $\hat{f}^{[0]}$ to the reported intercept estimate above for the logistic regression model).

A generalized additive model adds more flexibility to the regression function but is still interpretable. We fit a logistic additive model to the `wpbc` data as follows:

```
R> wpbc_gam <- gamboost(status ~ ., data = wpbc2, family = Binomial(), baselearner = "bss")
R> mopt <- mstop(aic <- AIC(wpbc_gam, "classical"))
R> aic
```

```
[1] 196.16
Optimal number of boosting iterations: 86
Degrees of freedom (for mstop = 86): 11.348
```

This model selected 15 out of 32 covariates. The partial contributions of the four most important variables are depicted in Figure 5 indicating a remarkable degree of non-linearity.

Illustration: Wisconsin prognostic breast cancer (cont.) Instead of the binary response variable describing the recurrence status, we make use of the additionally available time information for modeling the time to recurrence, i.e., all observations with non-recurrence are censored. First, we calculate IPC weights

```
R> library("survival")
R> ### calculate IPC weights
R> censored <- wpbc$status == "R"
R> iw <- IPCweights(Surv(wpbc$time, censored))
R> wpbc3 <- wpbc[,names(wpbc) != "status"]
```

and fit a weighted linear model by boosting with componentwise linear weighted least squares as base procedure:

```
R> ctrl <- boost_control(mstop = 500)
R> wpbc_surv <- glmboost(log(time) ~ ., data = wpbc3,
                        weights = iw, center = TRUE, control = ctrl)
R> mstop(aic <- AIC(wpbc_surv))
```

```
[1] 111
```

```
R> wpbc_surv <- wpbc_surv[mstop(aic)]
```

The following variables have been selected for fitting

```
R> names(coef(wpbc_surv)[abs(coef(wpbc_surv)) > 0])
```

```
 [1] "(Intercept)"         "mean_radius"
 [3] "mean_texture"        "mean_smoothness"
 [5] "mean_symmetry"       "SE_texture"
 [7] "SE_smoothness"       "SE_concavepoints"
 [9] "SE_symmetry"         "worst_concavepoints"
[11] "pnodes"
```

and the fitted values are depicted in Figure 6, showing a reasonable model fit.

Alternatively, a Cox model with linear predictor can be fitted using L_2Boosting by implementing the negative gradient of the partial likelihood (see Ridgeway (1999)) via

```
R> glmboost(Surv(wpbc$time, wpbc$status == "N") ~ .,
         data = wpbc, family = CoxPH(), center = TRUE)
```

For more examples, such as fitting an additive Cox model using **mboost**, see (Hothorn and Bühlmann, 2006).

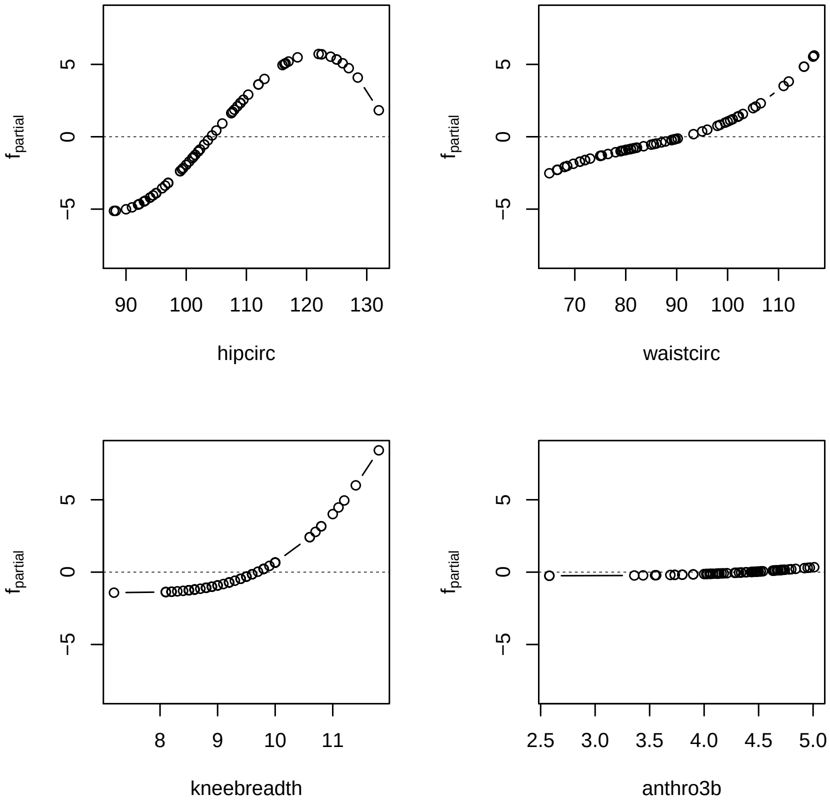

Figure 3: `bodyfat` data: Partial fits for a linear model fitted to transformed covariates using B-splines (without centering of estimated functions to mean zero).

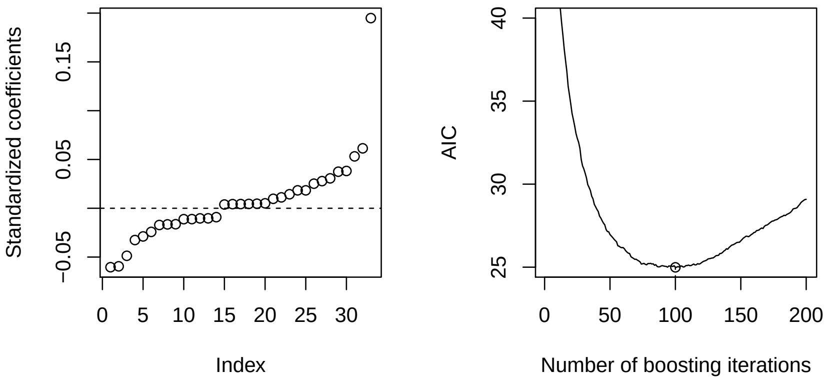

Figure 4: `westbc` data: Standardized regression coefficients $\hat{\beta}^{(j)}\sqrt{\widehat{\mathrm{Var}}(X^{(j)})}$ (left panel) for $m_{\mathrm{stop}} = 100$ determined from the classical AIC criterion shown in the right panel.

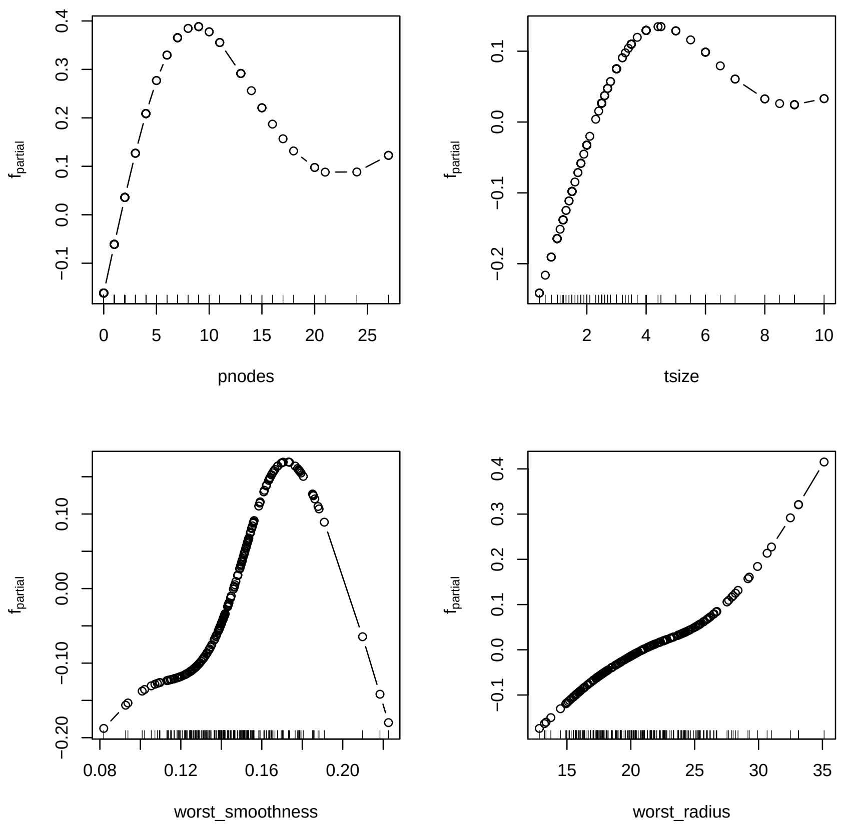

Figure 5: `wpbc` data: Partial contributions of four selected covariates in an additive logistic model (without centering of estimated functions to mean zero).

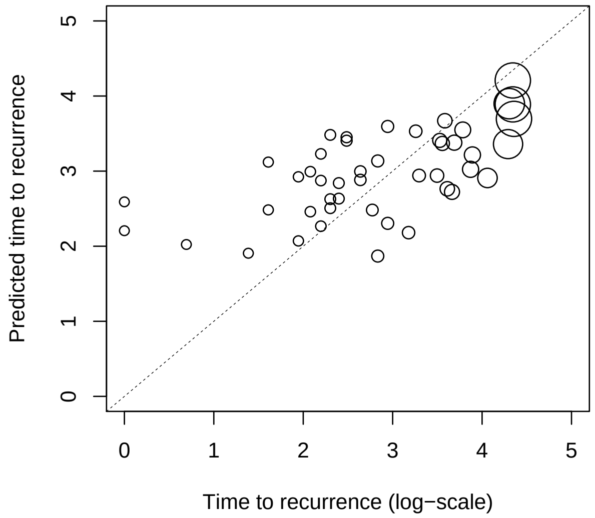

Figure 6: `wpbc` data: Fitted values of an IPC-weighted linear model, taking both time to recurrence and censoring information into account. The radius of the circles is proportional to the IPC weight of the corresponding observation, censored observations with IPC weight zero are not plotted.

References

C. L. Blake and C. J. Merz. UCI repository of machine learning databases, 1998. URL http://www.ics.uci.edu/~mlearn/MLRepository.html.

Peter Bühlmann and Torsten Hothorn. Boosting algorithms: Regularization, prediction and model fitting. *Statistical Science*, 22(4):477–505, 2007. doi: 10.1214/07-STS242.

A. L. Garcia, K. Wagner, T. Hothorn, C. Koebnick, H. J. Zunft, and U. Trippo. Improved prediction of body fat by measuring skinfold thickness, circumferences, and bone breadths. *Obesity Research*, 13(3):626–634, 2005.

R. C. Gentleman, V. J. Carey, D. M. Bates, B. Bolstad, M. Dettling, S. Dudoit, B. Ellis, L. Gautier, Y. Ge, J. Gentry, K. Hornik, T. Hothorn, M. Huber, S. Iacus, R. Irizarry, F. Leisch, C. Li, M. Mächler, A. J. Rossini, G. Sawitzki, C. Smith, G. Smyth, L. Tierney, J. Y. Yang, and J. Zhang. Bioconductor: open software development for computational biology and bioinformatics. *Genome Biology*, 5(10):R80, 2004.

T Hothorn and P Bühlmann. Model-based boosting in high dimensions. *Bioinformatics*, 22(22):2828–2829, Nov 2006. doi: doi:10.1093/bioinformatics/btl462.

G. Ridgeway. The state of boosting. *Computing Science and Statistics*, 31: 172–181, 1999.

Matthias Schmid and Torsten Hothorn. Boosting additive models using component-wise P-splines as base-learners. *Computational Statistics & Data Analysis*, 53(2):298–311, 2008.

W. N. Street, O. L. Mangasarian, , and W. H. Wolberg. An inductive learning approach to prognostic prediction. In *Proceedings of the Twelfth International Conference on Machine Learning*, pages 522–530, San Francisco, CA, 1995. Morgan Kaufmann Publishers Inc.

M. West, C. Blanchette, H. Dressman, E. Huang, S. Ishida, R. Spang, H. Zuzan, J. Olson, J. Marks, and J. Nevins. Predicting the clinical status of human breast cancer by using gene expression profiles. *Proceedings of the National Academy of Sciences (USA)*, 98:11462–11467, 2001.
