# Supplementary material for: A statistical model for the analysis of beta values in DNA methylation studies
Source: BMC Bioinformatics. 2016 Nov 22;17:480. doi: 10.1186/s12859-016-1347-4 (PMC5120494; doi:10.1186/s12859-016-1347-4)
Supplement: Additional file 3 — R package mboostDevel. (GZ 2038 kb) [file 12859_2016_1347_MOESM3_ESM.gz › mboostDevel/inst/doc/mboost_tutorial.pdf]

Model-based Boosting in R

A Hands-on Tutorial Using the R Package mboost

Benjamin Hofner*† Andreas Mayr† Nikolay Robinzonov‡
Matthias Schmid†

This is an extended and (slightly) modified version of the manuscript
Benjamin Hofner, Andreas Mayr, Nikolay Robinzonov and Mattthias Schmid (2014), Model-based Boosting in R - A Hands-on Tutorial Using the R Package mboost. *Computational Statistics*, 29:3-35.
DOI 10.1007/s00180-012-0382-5.
The final publication is available at `http://link.springer.com`.

Changes to the results in the original manuscript are due to changes in some defaults of **mboost** (most notably the definition of degrees of freedom has changed in **mboost** 2.2-0). See NEWS for details.

We provide a detailed hands-on tutorial for the R add-on package **mboost**. The package implements boosting for optimizing general risk functions utilizing component-wise (penalized) least squares estimates as base-learners for fitting various kinds of generalized linear and generalized additive models to potentially high-dimensional data. We give a theoretical background and demonstrate how **mboost** can be used to fit interpretable models of different complexity. As an example we use **mboost** to predict the body fat based on anthropometric measurements throughout the tutorial.

1 Introduction

A key issue in statistical research is the development of algorithms for model building and variable selection (Hastie et al. 2009). Due to the recent advances in computational research and biotechnology, this issue has become even more relevant (Fan and Lv 2010). For example, microarray and DNA sequencing experiments typically result in high-dimensional data sets with large numbers of predictor variables but relatively small sample sizes. In these experiments, it is usually of interest to build a good prognostic model using a small subset of marker genes. Hence, there is a need for statistical techniques to select the most informative features out of a large set of predictor variables. A related problem is to select the appropriate modeling alternative for each of the covariates ("model choice", Kneib et al. 2009). This problem even arises when data sets are not high-dimensional. For example, a continuous covariate could be included into a statistical model using linear, non-linear or interaction effects with other predictor variables.

In order to address these issues, a variety of regression techniques has been developed during the past years (see, e.g., Hastie et al. 2009). This progress in statistical methodology is primarily due to the fact that classical techniques for model building and variable selection (such as generalized linear modeling with stepwise selection) are known to be unreliable or might even be biased. In this tutorial, we consider *component-wise gradient boosting* (Breiman 1998, 1999, Friedman et al. 2000, Friedman 2001), which is a machine learning method for optimizing prediction accuracy and for obtaining statistical model estimates via gradient descent techniques. A key feature of the method is that it carries out variable selection during the fitting process (Bühlmann and Yu 2003, Bühlmann 2006) without relying on heuristic techniques such as stepwise variable selection. Moreover, gradient boosting algorithms result in prediction rules that have the same interpretation

*E-mail: benjamin.hofner@imbe.med.uni-erlangen.de
†Department of Medical Informatics, Biometry and Epidemiology, Friedrich-Alexander-Universität Erlangen-Nürnberg
‡Department of Statistics, Ludwig-Maximilians-Universität München

as common statistical model fits. This is a major advantage over machine learning methods such as random forests (Breiman 2001) that result in non-interpretable "black-box" predictions.

In this tutorial, we describe the R (R Development Core Team 2012) add-on package **mboost** (Hothorn et al. 2010, 2012), which implements methods to fit generalized linear models (GLMs), generalized additive models (GAMs, Hastie and Tibshirani 1990), and generalizations thereof using component-wise gradient boosting techniques. The **mboost** package can thus be used for regression, classification, time-to-event analysis, and a variety of other statistical modeling problems based on potentially high-dimensional data. Because of its user-friendly formula interface, **mboost** can be used in a similar way as classical functions for statistical modeling in R. In addition, the package implements several convenience functions for hyper-parameter selection, parallelization of computations, and visualization of results.

The rest of the paper is organized as follows: In Section 2, we provide a brief theoretical overview of component-wise gradient boosting and its properties. In Section 3, the **mboost** package is presented in detail. Here we present the infrastructure of the package and show how to use **mboost** to obtain interpretable statistical model fits. All steps are illustrated using a clinical data set that was collected by Garcia et al. (2005). The authors conducted a study to predict the body fat content of study participants by means of common anthropometric measurements. The response variable was obtained by Dual X-Ray Absorptiometry (DXA). DXA is an accurate but expensive method for measuring the body fat content. In order to reduce costs, it is therefore desirable to develop a regression equation for predicting DXA measurements by means of anthropometric measurements which are easy to obtain. Using the **mboost** package, we provide a step-by-step illustration on how to use gradient boosting to fit a prediction model for body fat. A summary of the paper is given in Section 4.

2 A Brief Theoretical Overview of Component-Wise Gradient Boosting

Throughout the paper, we consider data sets containing the values of an outcome variable y and some predictor variables $x_1, \ldots, x_p$. For example, in case of the body fat data, y is the body fat content of the study participants (measured using DXA) and $x_1, \ldots, x_p$ represent the following anthropometric measurements: age in years, waist circumference, hip circumference, breadth of the elbow, breadth of the knee, and four aggregated predictor variables that were obtained from other anthropometric measurements.

The aim is to model the relationship between y and $\mathbf{x} := (x_1, \ldots, x_p)^\top$, and to obtain an "optimal" prediction of y given $\mathbf{x}$. This is accomplished by minimizing a loss function $\rho(y, f) \in \mathbb{R}$ over a prediction function f (depending on $\mathbf{x}$). Usually, for GLMs and GAMs, the loss function is simply the negative log-likelihood function of the outcome distribution. Linear regression with a continuous outcome variable $y \in \mathbb{R}$ is a well-known example of this approach: Here, ρ corresponds to the least squares objective function (which is equivalent to the negative log-likelihood of a Gaussian model), and f is a parametric (linear) function of $\mathbf{x}$.

In the gradient boosting framework, the aim is to estimate the optimal prediction function f^* that is defined by

$$f^* := \operatorname{argmin}_f \mathbb{E}_{Y,\mathbf{X}} \left[\rho(y, f(\mathbf{x}^\top)) \right] , \tag{1}$$

where the loss function ρ is assumed to be differentiable with respect to f. In practice, we usually deal with realizations $(y_i, \mathbf{x}_i^\top)$, $i = 1, \ldots, n$, of $(y, \mathbf{x}^\top)$, and the expectation in (1) is therefore not known. For this reason, instead of minimizing the expected value given in (1), boosting algorithms minimize the observed mean $\mathcal{R} := \sum_{i=1}^n \rho(y_i, f(\mathbf{x}_i^\top))$ (also called the "empirical risk"). The following algorithm ("component-wise gradient boosting") is used to minimize $\mathcal{R}$ over f:

1. Initialize the function estimate $\hat{f}^{[0]}$ with offset values. Note that $\hat{f}^{[0]}$ is a vector of length n. In the following paragraphs, we will generally denote the vector of function estimates at iteration m by $\hat{f}^{[m]}$.

2. Specify a set of *base-learners*. Base-learners are simple regression estimators with a fixed set of input variables and a univariate response. The sets of input variables are allowed to differ among the base-learners. Usually, the input variables of the base-learners are small subsets of the set of predictor variables $x_1, \ldots, x_p$. For example, in the simplest case, there is exactly one base-learner for each predictor variable, and the base-learners are just simple linear models using the predictor variables as input variables. Generally, the base-learners considered in this paper are either penalized or unpenalized least squares estimators using small subsets of the predictor variables as input variables (see Section 3.2.1 for details and examples). Each base-learner represents a modeling alternative for the statistical model. Denote the number of base-learners by P and set $m = 0$.

3. Increase m by 1, where m is the number of iterations.

4. a) Compute the negative gradient $-\frac{\partial \rho}{\partial f}$ of the loss function and evaluate it at $\hat{f}^{[m-1]}(\mathbf{x}_i^\top)$, $i = 1, \ldots, n$ (i.e., at the estimate of the previous iteration). This yields the negative gradient vector

$$\mathbf{u}^{[m]} = \left(u_i^{[m]}\right)_{i=1,\ldots,n} := \left(-\frac{\partial}{\partial f}\rho\left(y_i, \hat{f}^{[m-1]}(\mathbf{x}_i^\top)\right)\right)_{i=1,\ldots,n} .$$

   b) Fit each of the P base-learners to the negative gradient vector, i.e., use each of the regression estimators specified in step 2 separately to fit the negative gradient. The resulting P regression fits yield P vectors of predicted values, where each vector is an estimate of the negative gradient vector $\mathbf{u}^{[m]}$.

   c) Select the base-learner that fits $\mathbf{u}^{[m]}$ best according to the residual sum of squares (RSS) criterion and set $\hat{\mathbf{u}}^{[m]}$ equal to the fitted values of the best-fitting base-learner.

   d) Update the current estimate by setting $\hat{f}^{[m]} = \hat{f}^{[m-1]} + \nu\,\hat{\mathbf{u}}^{[m]}$, where $0 < \nu \leq 1$ is a real-valued step length factor.

5. Iterate Steps 3 and 4 until the stopping iteration m_{stop} is reached (the choice of m_{stop} is discussed below).

From step 4 it is seen that an estimate of the true negative gradient of $\mathcal{R}$ is added to the current estimate of f^* in each iteration. Consequently, the component-wise boosting algorithm descends along the gradient of the empirical risk $\mathcal{R}$. The empirical risk $\mathcal{R}$ is thus minimized in a stage-wise fashion, and a structural (regression) relationship between y and $\mathbf{x}$ is established. This strategy corresponds to replacing classical Fisher scoring algorithms for maximum likelihood estimation of f^* (McCullagh and Nelder 1989) by a gradient descent algorithm in function space. As seen from steps 4(c) and 4(d), the algorithm additionally carries out variable selection and model choice, as only one base-learner is selected for updating $\hat{f}^{[m]}$ in each iteration. For example, if each base-learner corresponds to exactly one predictor variable (that is used as the input variable of the respective base-learner), only *one* predictor variable is selected in each iteration (hence the term "component-wise"). Note that the variable selection property of the above-described algorithm is not a general feature of all boosting methods (where, in principle, any type of regression estimator could be used to fit the negative gradient) but is caused by the specific definition of the base-learning mechanism in steps 2 and 4.

Due to the additive update, the final boosting estimate in iteration m_{stop} can be interpreted as an additive prediction function. It is easily seen that the structure of this function is equivalent to the structure of the additive predictor of a GAM (see Hastie and Tibshirani 1990). This means that

$$\hat{f} = \hat{f}_1 + \cdots + \hat{f}_P \ , \tag{2}$$

where $\hat{f}_1, \ldots, \hat{f}_P$ correspond to the functions specified by the base-learners. Consequently, $\hat{f}_1, \ldots, \hat{f}_P$ depend on the predictor variables that were used as input variables of the respective base-learners. Note that a base-learner can be selected multiple times in the course of the boosting algorithm. In this case, its function estimate $\hat{f}_j$, $j \in 1, \ldots, P$, is the sum of the individual estimates $\nu \cdot \hat{\mathbf{u}}^{[m-1]}$ obtained in the iterations in which the base-learner was selected. Note also that some of the $\hat{f}_j$, $j = 1, \ldots, P$, might be equal to zero, as the corresponding base-learners might not have been selected in step 4(c). This can then be considered as variable selection or model choice (depending on the specification of the base-learners).

From step 4 of the component-wise gradient boosting algorithm it is clear that the specification of the base-learners is crucial for interpreting the model fit. As a general rule, due to the additive update in step 4(d), the estimate of a function f_j at iteration m_{stop} has the same structure as the corresponding base-learner. For example, f_j is a linear function if the base-learner used to model f_j in step 4(b) is a simple linear model (see Bühlmann and Hothorn 2007, p. 484, also see Section 3 for details). Similarly, f_j is a smooth function of the jth covariate $\mathbf{x}_j$ if the corresponding base-learner is smooth as well. Note that it is generally possible to incorporate observation weights into component-wise gradient boosting. This is accomplished by using weighted regression techniques for each of the base-learners in step 4.

A crucial issue is the choice of the stopping iteration m_{stop}. Several authors have argued that boosting algorithms should generally not be run until convergence. Otherwise, overfits resulting in a suboptimal prediction accuracy would be likely to occur (see Bühlmann and Hothorn 2007). We therefore use a finite stopping iteration for component-wise gradient boosting that optimizes the prediction accuracy ("early stopping strategy"). As a consequence, the stopping iteration becomes a tuning parameter of the algorithm, and cross-validation techniques or AIC-based techniques can be used to estimate the optimal m_{stop}. In contrast

to the choice of the stopping iteration, the choice of the step length factor ν has been shown to be of minor importance for the predictive performance of a boosting algorithm. The only requirement is that the value of ν is small (e.g., $\nu = 0.1$, see Schmid and Hothorn 2008b). Small values of ν are necessary to guarantee that the algorithm does not overshoot the minimum of the empirical risk $\mathcal{R}$.

A major consequence of early stopping is that boosting optimizes prediction accuracy by regularizing the estimates of f^* via shrinkage techniques. This is due to the fact that using a small step length ν ensures that effect estimates increase "slowly" in the course of the boosting algorithm, and that the estimates stop increasing as soon as the optimal stopping iteration m_{stop} is reached. Stopping a component-wise gradient boosting algorithm at the optimal iteration therefore implies that effect estimates are shrunken such that the predictive power of the GAM is maximal. Shrinking estimates is a widely used strategy for building prognostic models: Estimates tend to have a slightly increased bias but a decreased variance, thereby improving prediction accuracy. In addition, shrinkage generally stabilizes effect estimates and avoids multicollinearity problems. Despite the bias induced by shrinking effect estimates, however, the structure of function (2) ensures that results are interpretable and that black-box estimates are avoided. The interpretation of boosting estimates is essentially the same as those of classical maximum likelihood estimates.

3 The Package mboost

As pointed out above, the R add-on package **mboost** implements model-based boosting methods as introduced above that result in interpretable models. This is in contrast to other boosting packages such as **gbm** (Ridgeway 2010), which implements tree-based boosting methods that lead to black-box predictions. The **mboost** package offers a modular nature that allows to specify a wide range of statistical models.

A generalized additive model is specified as the combination of a distributional assumption and a structural assumption. The distributional assumption specifies the conditional distribution of the outcome. The structural assumption specifies the types of effects that are to be used in the model, i.e., it represents the deterministic structure of the model. Usually, it specifies how the predictors are related to the conditional mean of the outcome. To handle a broad class of models within one framework, **mboost** also allows to specify effects for conditional quantiles, conditional expectiles or hazard rates. The distributional assumption, i.e., the loss function that we want to minimize, is specified as a `family`. The structural assumption is given as a `formula` using base-learners.

The loss function, as specified by the `family` is independent of the estimation of the base-learners. As one can see in the component-wise boosting algorithm, the loss function is only used to compute the negative gradient in each boosting step. The predictors are then related to these values by penalized ordinary least squares estimation, irrespective of the loss function. Hence, the user can freely combine structural and distributional assumptions to tackle new estimation problems.

In Section 3.1 we will derive a special case of component-wise boosting to fit generalized linear models. In Section 3.2 we introduce the methods to fit generalized additive models and give an introduction to the available base-learners. How different loss functions can be specified is shown in Section 3.4.

3.1 Fitting Generalized Linear Models: `glmboost`

The function `glmboost()` provides an interface to fit (generalized) linear models. A (generalized) linear model of the covariates $\mathbf{x} = (x_1, \ldots, x_p)^\top$ has the form

$$g(\vec{\mu}) = \beta_0 + \beta_1 x_1 + \ldots + \beta_p x_p$$

with the (conditional) expectation of the response $\vec{\mu} = \mathbb{E}(y|\vec{x})$, the link function g and parameters $\vec{\beta}$. The resulting models from `glmboost()` can be essentially interpreted the same way as models that are derived from `glm()`. The only difference is that the boosted generalized linear model additionally performs variable selection as described in Section 2 and the effects are shrunken toward zero if early stopping is applied. In each boosting iteration, `glmboost()` fits simple linear models without intercept *separately for each column of the design matrix* to the negative gradient vector. Only the best-fitting linear model (i.e., the best fitting base-learner) is used in the update step. If factors are used in the model, this results in separate updates for the effects of each factor level as these are (after dummy coding) represented by separate columns in the design matrix. If one wants to treat factors or groups of variables as one base-learner with a common update, one needs to use the `gamboost()` function with a `bols()` base-learner for each factor or group (see Section 3.2, especially Table 2, for details).

The interface of `glmboost()` is in essence the same as for `glm()`. Before we show how one can use the function to fit a linear model to predict the body fat content, we give a short overview on the function[1]:

[1] Note that here and in the following we sometimes restrict the focus to the most important or most interesting arguments of a

```
glmboost(formula, data = list(), weights = NULL,
         center = TRUE, control = boost_control(), ...)
```

The model is specified using a `formula` as in `glm()` of the form `response ~ predictor1 + predictor2` and the data set is provided as a `data.frame` via the `data` argument. Optionally, `weights` can be given for weighted regression estimation. The argument `center` is specific for `glmboost()`. It controls whether the data is internally centered. Centering is of great importance, as this allows much faster "convergence" of the algorithm or even ensures that the algorithm converges in the direction of the true value at all. We will discuss this in detail at the end of Section 3.1. The second boosting-specific argument, `control`, allows to define the hyper-parameters of the boosting algorithm. This is done using the function `boost_control()`. For example one could specify:

```
R> boost_control(mstop = 200,  ## initial number of boosting iterations. Default: 100
+               nu = 0.05,     ## step length. Default: 0.1
+               trace = TRUE) ## print status information? Default: FALSE
```

Finally, the user can specify the distributional assumption via a `family`, which is "hidden" in the '`...`' argument (see `?mboost_fit`[2] for details and further possible parameters). The default `family` is `Gaussian()`. Details on families are given in Section 3.4. Ways to specify new families are described in the Appendix.

Case Study: Prediction of Body Fat

The aim of this case study is to compute accurate predictions for the body fat of women based on available anthropometric measurements. Observations of 71 German women are available with the data set `bodyfat` (Garcia et al. 2005) included in **mboost**. We first load the package and the data set[3].

```
R> library("mboostDevel")                          ## load package
R> data("bodyfat", package = "TH.data")      ## load data
```

The response variable is the body fat measured by DXA (`DEXfat`), which can be seen as the gold standard to measure body fat. However, DXA measurements are too expensive and complicated for a broad use. Anthropometric measurements as waist or hip circumferences are in comparison very easy to measure in a standard screening. A prediction formula only based on these measures could therefore be a valuable alternative with high clinical relevance for daily usage. The available variables and anthropometric measurements in the data set are presented in Table 1.

Table 1: Available variables in the `bodyfat` data, for details see Garcia et al. (2005).

Name	Description
`DEXfat`	body fat measured by DXA (response variable)
`age`	age of the women in years
`waistcirc`	waist circumference
`hipcirc`	hip circumference
`elbowbreadth`	breadth of the elbow
`kneebreadth`	breadth of the knee
`anthro3a`	sum of logarithm of three anthropometric measurements
`anthro3b`	sum of logarithm of three anthropometric measurements
`anthro3c`	sum of logarithm of three anthropometric measurements
`anthro4`	sum of logarithm of four anthropometric measurements

In the original publication (Garcia et al. 2005), the presented prediction formula was based on a linear model with backward-elimination for variable selection. The resulting final model utilized hip circumference (`hipcirc`), knee breadth (`kneebreadth`) and a compound covariate (`anthro3a`), which is defined as the sum of the logarithmic measurements of chin skinfold, triceps skinfold and subscapular skinfold:

function. Further arguments might exist. Thus, for a complete list of arguments and their description refer to the respective manual.

[2] `glmboost()` merely handles the preprocessing of the data. The actual fitting takes place in a unified framework in the function `mboost_fit()`.

[3] The data set `bodyfat` has been moved to the package **TH.data**.

```
R> ## Reproduce formula of Garcia et al., 2005
R> lm1 <- lm(DEXfat ~ hipcirc  + kneebreadth + anthro3a, data = bodyfat)
R> coef(lm1)

(Intercept)     hipcirc kneebreadth    anthro3a
  -75.23478     0.51153     1.90199     8.90964
```

A very similar model can be easily fitted by boosting, applying `glmboost()` with default settings:

```
R> ## Estimate same model by glmboost
R> glm1 <- glmboost(DEXfat ~ hipcirc  + kneebreadth + anthro3a, data = bodyfat)
R> coef(glm1, off2int=TRUE)    ## off2int adds the offset to the intercept

(Intercept)     hipcirc kneebreadth    anthro3a
  -75.20734     0.51149     1.90054     8.90713
```

Note that in this case we used the default settings in `control` and the default family `Gaussian()` leading to boosting with the L_2 loss.

We now want to consider all available variables as potential predictors. One way is to simply specify "." on the right side of the formula:

```
R> glm2 <- glmboost(DEXfat ~ ., data = bodyfat)
```

As an alternative one can explicitly provide the whole formula by using the `paste()` function[4]. Therefore, one could essentially call:

```
R> preds <- names(bodyfat[, names(bodyfat) != "DEXfat"])        ## names of predictors
R> fm <- as.formula(paste("DEXfat ~", paste(preds, collapse = "+")))  ## build formula
R> fm

DEXfat ~ age + waistcirc + hipcirc + elbowbreadth + kneebreadth +
    anthro3a + anthro3b + anthro3c + anthro4
```

and provide `fm` to the `formula` argument in `glmboost()`. Note that a solution using the `paste()` function is somewhat unavoidable when we intend to combine different base-learners for plenty of predictors in `gamboost()`.

Note that at this iteration (`mstop` is still 100 as it is the default value) `anthro4` is not included in the resulting model as the corresponding base-learner was never selected in the update step. The function `coef()` by default only displays the selected variables but can be forced to show all effects by specifying `which = ""`:

```
R> coef(glm2,        ## usually the argument 'which' is used to specify single base-
+       which = "")  ## learners via partial matching; With which = "" we select all.

 (Intercept)          age    waistcirc      hipcirc elbowbreadth  kneebreadth
  -98.816608     0.013602     0.189716     0.351626    -0.384140     1.736589
    anthro3a     anthro3b     anthro3c      anthro4
    3.326860     3.656524     0.595363     0.000000
attr(,"offset")
[1] 30.783
```

A plot of the coefficient paths, similar to the ones commonly known from the LARS algorithm (Efron et al. 2004), can be easily produced by using `plot()` on the `glmboost` object (see Figure 1):

```
R> plot(glm2, off2int = TRUE)    ## default plot, offset added to intercept
R> ## now change ylim to the range of the coefficients without intercept (zoom-in)
R> plot(glm2, ylim = range(coef(glm2, which = preds)))
```

[4] Another alternative is given by the matrix interface for `glmboost()` where one can directly use the design matrix as an argument. For details see `?glmboost`.

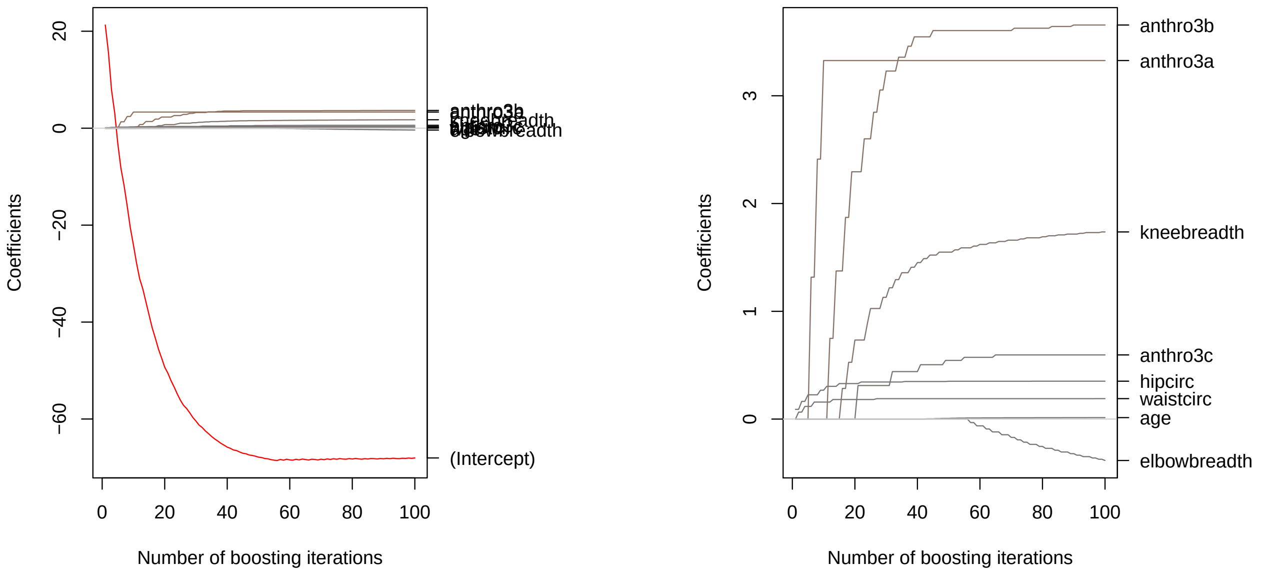

Figure 1: Coefficients paths for the body fat data: Here, the default plot on the left leads to hardly readable variable names due to the inclusion of the intercept. For the plot on the right we therefore adjusted the y-scale to avoid this problem.

Centering of Linear Base-learners Without Intercept

For linear base-learners that are specified without intercept,[5] it is of great importance to center the covariates before fitting the model. Without centering of the covariates, linear effects that result from base-learners without intercept are forced through the origin (with no data lying there). Hence, the convergence will be very slow or the algorithm will not converge to the "correct" solution even in very simple cases. As an example, consider one normally distributed predictor $\mathbf{x} = (x_1, \dots, x_n)^\top$, and a model

$$\mathbf{y} = \beta\mathbf{x} + \boldsymbol{\varepsilon},$$

with $\beta = 1$ and $\boldsymbol{\varepsilon} \sim \mathcal{N}(\mathbf{0}, 0.3^2)$. Usually, a model without intercept could be fitted to estimate β. However, if we apply boosting with the L_2 loss the negative gradient in the first boosting step is, by default, the centered response, i.e., $\mathbf{u}^{[1]} = \mathbf{y} - 1/n\sum_{i=1}^{n} y_i$. For other loss functions the negative gradient in the first boosting iteration is not exactly the mean-centered response. Yet, the negative gradient in the first step is always "centered" around zero. In this situation, the application of a base-learner without intercept (i.e., a simple linear model without intercept) is not sufficient anymore to recover the effect β (see Figure 2(a)). The true effect is completely missed. To solve this problem, it is sufficient to use a centered predictor $\mathbf{x}$. Then, the center of the data is shifted to the origin (see Figure 2(b)) and the model without intercept goes through the origin as well.

Centering the predictors does not change the estimated effects of the predictors. Yet, the intercept needs to be corrected as can be seen from the following example. Consider two predictors and estimate a model with centered predictors, i.e,

$$\begin{aligned} \hat{\mathbf{y}} &= \hat{\beta}_0 + \hat{\beta}_1(\mathbf{x}_1 - \bar{x}_1) + \hat{\beta}_2(\mathbf{x}_2 - \bar{x}_2) \qquad \Leftrightarrow \\ \hat{\mathbf{y}} &= \underbrace{(\hat{\beta}_0 - \hat{\beta}_1\bar{x}_1 - \hat{\beta}_2\bar{x}_2)}_{=\hat{\beta}_0^*} + \hat{\beta}_1\mathbf{x}_1 + \hat{\beta}_2\mathbf{x}_2. \end{aligned}$$

Hence, the intercept from a model without centering of the covariates equals $\hat{\beta}_0^* = \hat{\beta}_0 - \sum_j \hat{\beta}_j\bar{x}_j$, where $\hat{\beta}_0$ is the intercept estimated from a model with centered predictors.

3.2 Fitting Generalized Additive Models: `gamboost`

Besides an interface to fit linear models, **mboost** offers a very flexible and powerful function to fit additive models. An additive model of the covariates $\mathbf{x} = (x_1, \dots, x_p)^\top$ has, in general, the form

$$g(\vec{\mu}) = \beta_0 + f_1 + \ldots + f_p$$

[5] If the fitting function `glmboost()` is used the base-learners never contain an intercept. Furthermore, linear base-learners without intercept can be obtained by specifying a base-learner `bols(x, intercept = FALSE)` (see below).

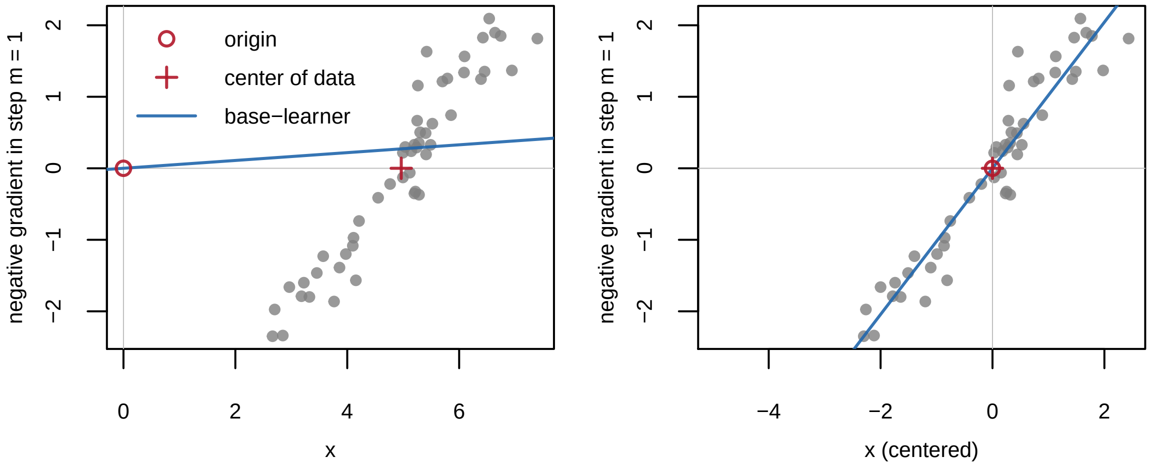

(a) Without Centering (b) With Centering

Figure 2: L_2Boosting in the first boosting step, i.e., with centered response variable as outcome. A base-learner without intercept misses the true effect completely if $\mathbf{x}$ is not centered (left) and is able to capture the true structure if $\mathbf{x}$ is centered (right).

with the (conditional) expectation of the response $\vec{\mu} = \mathbb{E}(y|\vec{x})$, the link function g and arbitrary functions $f_1, \ldots, f_p$ of the covariates. These functions include simple, linear functions as well as smooth, non-linear functions. The functions are specified by the base-learners that are introduced in the following paragraphs.

The function `gamboost()` can be used to fit linear models or (non-linear) additive models via component-wise boosting. The user additionally needs to state which variable should enter the model in which fashion, e.g. as a linear effect or as a smooth effect. In general, however, the interface of `gamboost()` is very similar to `glmboost()`.

```
gamboost(formula, data = list(), ...)
```

Again, the function requires a formula to specify the model. Furthermore, a data set needs to be specified as for linear models. Additional arguments that are passed to `mboost_fit()`[6] include `weights`, `control` and `family`. These arguments can be used in the same way as described for `glmboost()` above.

3.2.1 Base-learners

The structural assumption of the model, i.e., the types of effects that are to be used, can be specified in terms of base-learners. Each base-learner results in a related type of effect. An overview of available base-learners is given in the following paragraphs. An example of how these base-learners can then be combined to formulate the model is given afterwards.

The base-learners should be defined such that the degrees of freedom of the single base-learner are small enough to prevent overshooting. Typically one uses, for example, 4 degrees of freedom (the default for many base-learners) or less. Despite the small initial degrees of freedom, the final estimate that results from this base-learner can adopt higher order degrees of freedom due to the iterative nature of the algorithm. If the base-learner is chosen multiple times, it overall adapts to an appropriate degree of flexibility and smoothness.

All base-learners considered in this tutorial are simple penalized least squares models of the form

$$\hat{\mathbf{u}} = \mathbf{X}(\mathbf{X}^\top\mathbf{X} + \lambda\mathbf{K})^{-1}\mathbf{X}^\top\mathbf{u} = \mathcal{S}\mathbf{u},$$

where the hat-matrix is defined as $\mathcal{S} = \mathbf{X}(\mathbf{X}^\top\mathbf{X} + \lambda\mathbf{K})^{-1}\mathbf{X}^\top$, with design matrix $\mathbf{X}$, penalty parameter λ and penalty matrix $\mathbf{K}$. The design and penalty matrices depend on the type of base-learner. A penalty parameter $\lambda = 0$ results in unpenalized estimation.

Linear and Categorical Effects

The `bols()` function[7] allows the definition of (penalized) ordinary least squares base-learners. Examples of base-learners of this type include (a) linear effects, (b) categorical effects, (c) linear effects for groups of

[6] `gamboost()` also calls `mboost_fit()` for the actual boosting algorithm.
[7] the name refers to **o**rdinary **l**east **s**quares base-learner

variables $\mathbf{x} = (x_1, x_2, \ldots, x_p)^\top$, (d) ridge-penalized effects for (b) and (c), (e) varying coefficient terms (i.e., interactions). If a penalized base-learner is specified, a special penalty based on the differences of the effects of adjacent categories is automatically used for ordinal variables (`x <- as.ordered(x)`) instead of the ridge penalty (Hofner et al. 2011a). Figure 3 shows two effect estimates that result from `bols()` base-learners, a simple linear effect and an effect estimate for a factor variable. The call to an ordinary penalized least squares base-learner is as follows:

```
bols(..., by = NULL, intercept = TRUE, df = NULL, lambda = 0)
```

The variables that correspond to the base-learner are specified in the '`...`' argument, separated by commas. If multiple variables are specified, they are treated as *one group*. A linear model is fitted for all given variables together and they are either all updated or not at all. An additional variable that defines varying coefficients can optionally be given in the `by` argument. The logical variable `intercept` determines whether an intercept is added to the design matrix (`intercept = TRUE`, the default). If `intercept = FALSE`, continuous covariates should be (mean-) centered as discussed above. This must be done 'by hand' in advance of fitting the model. The impact of the penalty in case of penalized OLS (ordinary least squares) base-learners can be determined either via the degrees of freedom `df` or the penalty parameter `lambda`. If degrees of freedom are specified, the penalty parameter `lambda` is computed from `df`[8]. Note that per default unpenalized linear models are used. Two definitions of degrees of freedom are implemented in **mboost**: The first uses the trace of the hat-matrix (trace($\mathcal{S}$)) as degrees of freedom, while the second definition uses trace($2\mathcal{S} - \mathcal{S}^\top\mathcal{S}$). The latter definition is tailored to the comparison of models based on residual sums of squares and hence is better suitable in the boosting context (see Hofner et al. 2011a, for details).

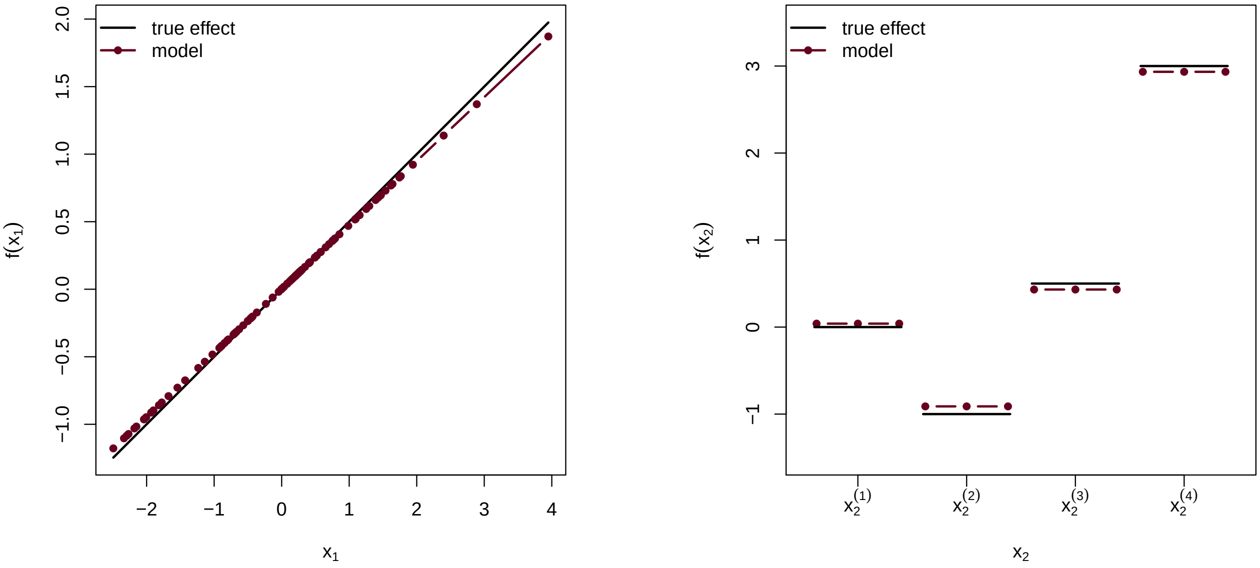

Figure 3: Examples for linear and categorical effects estimated using `bols()`: Linear effect (left; $f(x_1) = 0.5x_1$) and categorical effect (right; $f(x_2) = 0x_2^{(1)} - 1x_2^{(2)} + 0.5x_2^{(3)} + 3x_2^{(4)}$). The effects can be estimated with base-learners `bols(x1)` and `bols(x2)`, where `x1` is a continuous covariate and `x2` is a categorical covariate coded as `factor` in R.

Table 2 shows some calls to `bols()` and gives the resulting type of effect. To gain more information on a specific base-learner it is possible to inspect the base-learners in **mboost** using the function `extract()` on a base-learner or a boosting model. With the function one can extract, for example, the design matrices, the penalty matrices and many more characteristics of the base-learners. To extract, for example, the design matrix that results from a linear base-learner of a factor `z`, say `z <- factor(1:3)`, we can use

```
R> extract(bols(z))

  (Intercept) z2 z3
1           1  0  0
2           1  1  0
3           1  0  1
attr(,"assign")
```

[8]If `df` is specified in `bols()`, `lambda` is always ignored.

```
[1] 0 1 1
attr(,"contrasts")
attr(,"contrasts")$z
[1] "contr.treatment"
```

Thus, we see that base-learners of factors use treatment contrasts per default. For a detailed instruction on how to use the function see the manual for `extract()` and especially the examples therein.

Table 2: Some examples of effects that result from `bols()`

Call	Type of Effect
`bols(x)`	linear effect: $\mathbf{x}^\top \boldsymbol{\beta}$ with $\mathbf{x}^\top = (1, x)$
`bols(x, intercept = FALSE)`	linear effect without intercept: $\beta \cdot x$
`bols(z)`	OLS fit with factor z (i.e., linear effect after dummy coding)
`bols(z, df = 1)`	ridge-penalized OLS fit with one degree of freedom and factor z; If z is an ordered factor a difference penalty is used instead of the ridge penalty.
`bols(x1, x2, x3)`	one base-learner for three variables (group-wise selection): $\mathbf{x}^\top \boldsymbol{\beta}$ with $\mathbf{x}^\top = (1, x_1, x_2, x_3)$
`bols(x, by = z)`	interaction: $\mathbf{x}^\top \boldsymbol{\beta} \cdot z$ (with continuous variable z). If z is a factor, a separate effect is estimated for each factor level; Note that in this case, the main effect needs to be specified additionally via `bols(x)`.

Smooth Effects

The `bbs()` base-learner[9] allows the definition of smooth effects (i.e., functions of the covariate that are required to be sufficiently smooth, see Figure 4) based on B-splines (de Boor 1978) with difference penalty (i.e., P-splines, cf. Eilers and Marx 1996). P-splines can be seen as a versatile modeling tool for smooth effects. A wide class of effects can be specified using P-splines. Examples include (a) smooth effects, (b) bivariate smooth effects (e.g., spatial effects), (c) varying coefficient terms, (d) cyclic effects (= periodic effects) and many more. Two examples of smooth function estimates fitted using a `bbs()` base-learner are given in Figure 4. The call to a P-spline base-learner is as follows:

```
bbs(..., by = NULL, knots = 20, boundary.knots = NULL,
    degree = 3, differences = 2, df = 4, lambda = NULL,
    center = FALSE, cyclic = FALSE)
```

As for all base-learners, the variables that correspond to the base-learner can be specified in the '`...`' argument. Usually, only one variable is specified here for smooth effects, and at maximum two variables are allowed for `bbs()`. Varying coefficient terms $f(x) \cdot z$ can be specified using `bbs(x, by = z)`. In this case, x is called the effect modifier of the effect of z. The `knots` argument can be used to specify the number of equidistant knots, or the positions of the interior knots. In case of two variables, one can also use a named list to specify the number or the position of the interior knots for each of the variables separately. For an example of the usage of a named list see Section 3.2.2. The location of boundary knots (default: range of the data) can be specified using `boundary.knots`. Usually, no user-input is required here. The only exception is given for cyclic splines (see below). The degree of the B-spline bases (`degree`) and the order of the difference penalty (`differences`; $\in \{0, 1, 2, 3\}$) can be used to specify further characteristics of the spline estimate. The latter parameter specifies the type of boundary effect. For `differences = 2`, for example, deviations from a linear function are penalized. In the case of first order differences, deviations from a constant are subject to penalization. The smoothness of the base-learner can be specified using degrees of freedom (`df`) or the smoothing parameter (`lambda`)[10].

An issue in the context of P-splines is that one cannot make the degrees of freedom arbitrary small. A polynomial of order `differences - 1` always remains unpenalized (i.e., the so-called null space). As we usually apply second order differences, a linear effect (with intercept) remains unpenalized and thus df ≥ 2

[9] the name refers to B-splines with penalty, hence the second b

[10] If `lambda` is specified in `bbs()`, `df` is always ignored.

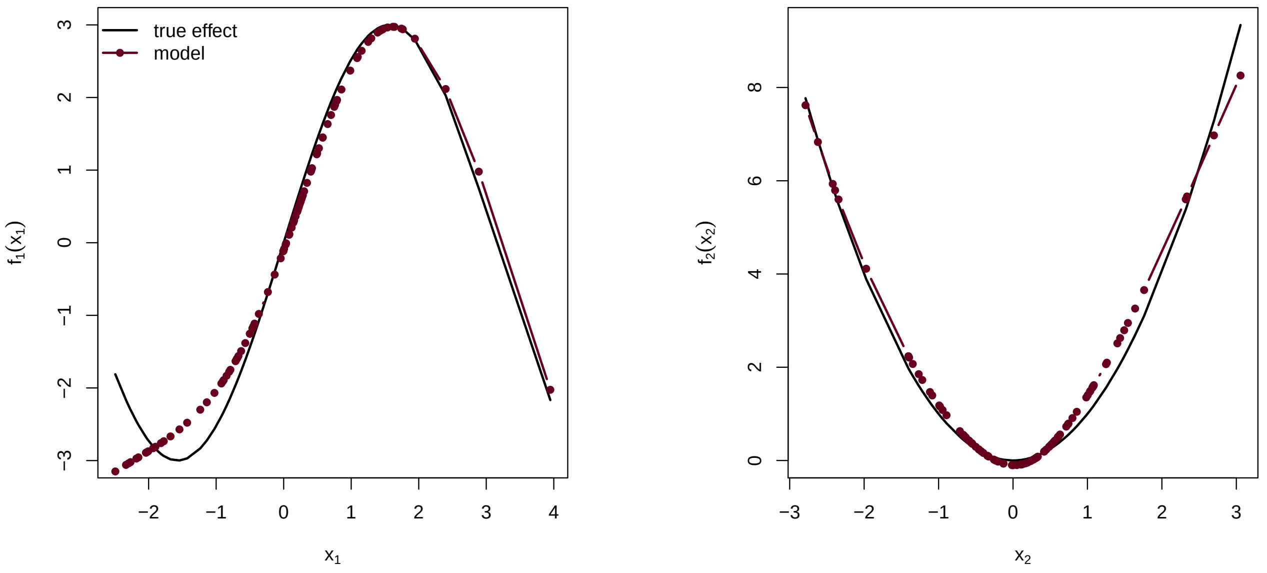

Figure 4: Examples for smooth effects estimated using `bbs(x1)` (left) and `bbs(x2, knots = quantile(x2, c(0.25, 0.5, 0.75)), df = 5)` (right). Hence, the left P-spline uses the default values of 20 equidistant inner knots and 4 degrees of freedom, while the right P-spline estimate is derived with 3 inner knots placed at the quartiles and 5 degrees of freedom. True effects: $f_1(x_1) = 3 \cdot \sin(x_1)$ (left) and $f_2(x_2) = x_2^2$ (right).

for all smoothing parameters. A solution is given by a P-spline decomposition (see Kneib et al. 2009; Hofner et al. 2011a). The smooth effect $f(x)$ is decomposed in the unpenalized polynomial and a smooth deviation from this polynomial. For differences of order 2 (= default), we thus get:

$$f(x) = \underbrace{\beta_0 + \beta_1 x}_{\text{unpenalized polynomial}} + \underbrace{f_{\text{centered}}(x)}_{\text{smooth deviation}} \tag{3}$$

The unpenalized polynomial can then be specified using `bols(x, intercept = FALSE)` and the smooth deviation is obtained by `bbs(x, center = TRUE)`. Additionally, it is usually advised to specify an explicit base-learner for the intercept (see Section 3.2.2).

A special P-spline base-learner is obtained if `cyclic = TRUE`. In this case, the fitted values are forced to coincide at the boundary knots and the function estimate is smoothly joined (see Figure 5). This is especially interesting for time-series or longitudinal data were smooth, periodic functions should be modeled. In this case it is of great importance that the boundary knots are properly specified to match the points were the function should be joined (due to subject matter knowledge).

An non-exhaustive overview of some usage scenarios for `bbs()` base-learners is given in Table 3.

Table 3: Some examples of effects that result from `bbs()`

Call	Type of Effect
`bbs(x, by = z)`	varying coefficient: $f(x) \cdot z = \beta(x)z$ (with continuous variable z). If z is a factor, a separate smooth effect is estimated for each factor level; Note that in this case, the main effect needs to be specified additionally via `bbs(x)`.
`bbs(x, knots = 10)`	smooth effect with 10 inner knots
`bbs(x, boundary.knots = c(0, 2 * pi), cyclic = TRUE)`	periodic function with period 2π
`bbs(x, center = TRUE, df = 1)`	P-spline decomposition (`center = TRUE`), which is needed to specify arbitrary small `df` for P-splines

Smooth Surface Estimation

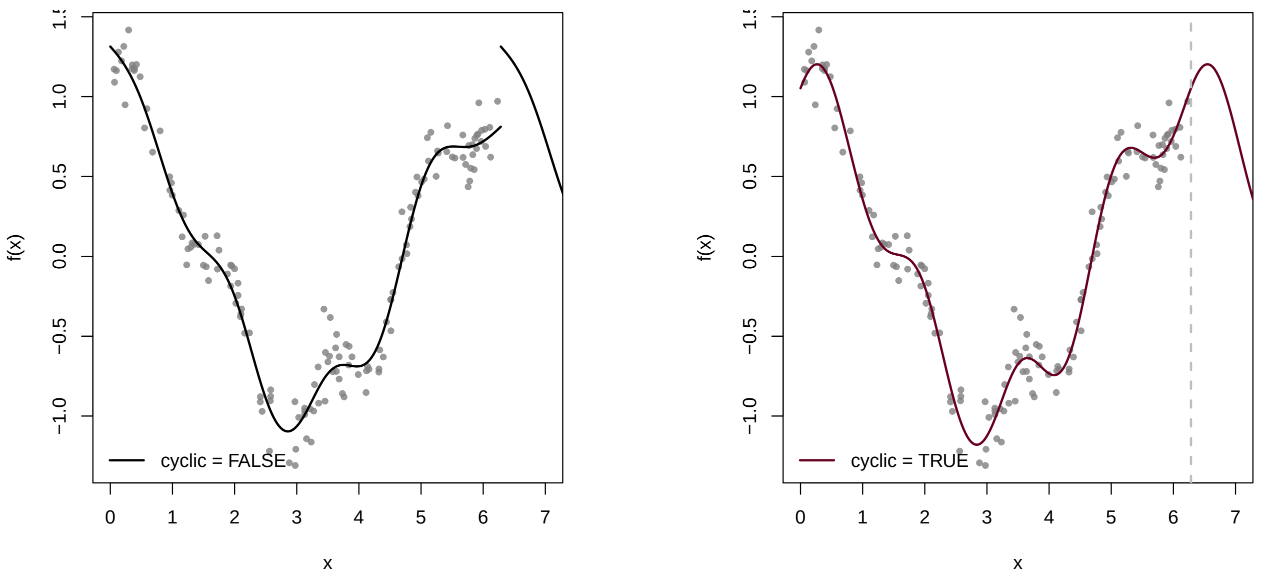

Figure 5: Example for cyclic splines. The unconstrained effect in the left figure is fitted using `bbs(x, knots = 12)`, the cyclic effect in the right is estimated using `bbs(x, cyclic = TRUE, knots = 12, boundary.knots = c(0, 2*pi))`. True effect: $f(x) = \sin(x)$.

An extension of P-splines to two dimensions is given by bivariate P-splines. They allow to fit spatial effects and smooth interaction surfaces. An example of a two dimensional function estimate is given in Figure 6. For further details on bivariate P-splines in the boosting context see Kneib et al. (2009). The effects can be obtained from a call to

```
bspatial(..., df = 6)
```

To specify two dimensional smooth effects, the '`...`' argument requires two variables, i.e., a call such as `bspatial(x, y)`. Note that `bspatial()` is just a wrapper to `bbs()` with redefined degrees of freedom[11]. Thus, all arguments from `bbs()` exist and can be used for `bspatial()`. An example is given in Section 3.2.2.

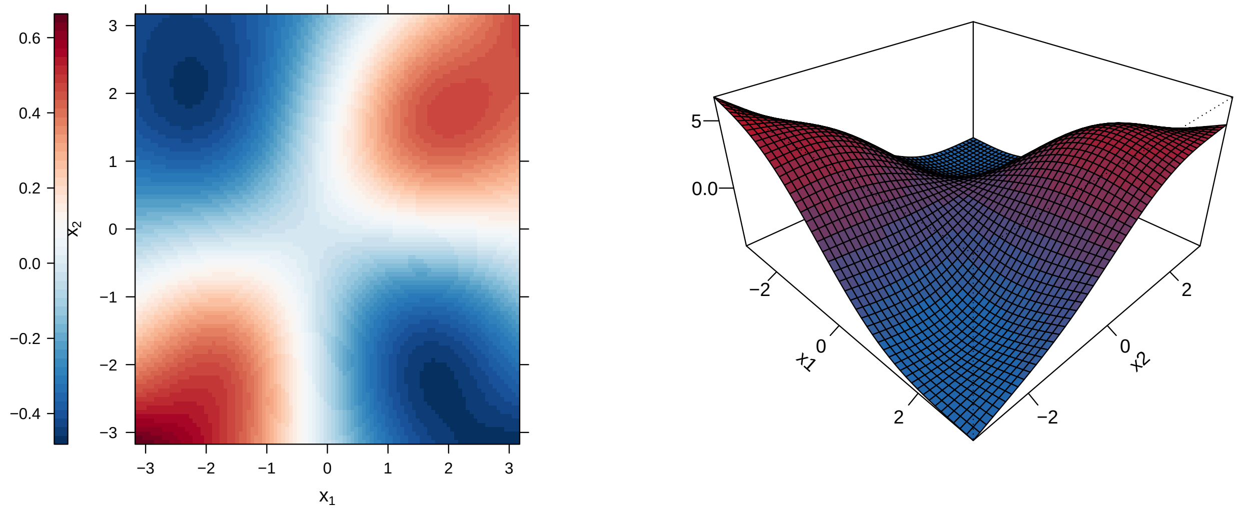

Figure 6: Example for interaction surface fitted using `bspatial()`. Two displays of the same function estimate are presented (levelplot: left; perspective plot: right). True function: $f(x_1, x_2) = \sin(x_1) \cdot \sin(x_2)$.

Random Effects

[11]Note that `df = 4` was changed to `df = 6` in **mboost** 2.1-0.

To specify random intercept or random slope terms in **mboost**, one can use a random effects base-learner. Random effects are used to model subject specific effects (in contrast to the usual – fixed – effects which can be seen as population specific effects). Usually, one is only interested in modeling the increased variability but not in the effect estimate of a specific individual as this effect is not transferable. For a comprehensive overview of random effects models we refer to Pinheiro and Bates (2000). Random effects are modeled as ridge-penalized group-specific effects. One can show that these coincide with standard random effects. The ridge penalty parameter is then simply the ratio of the error variance σ^2 and the random effects variance τ^2. Hence, the penalty parameter is inversely linked to τ^2 (and the degrees of freedom are directly linked to τ^2). As for all base-learners, we initially specify a small value for the degrees of freedom, i.e., we set a small random effects variance (relative to the residual variance). By repeatedly selecting the base-learner, the boosting algorithm can then adapt to the "true" random effects variance. For more details see Kneib et al. (2009, Web Appendix). We can specify random effects base-learners with a call to

```
brandom(..., df = 4)
```

As `brandom()` is just a wrapper of `bols()` with redefined degrees of freedom, one can use all arguments that are available for `bols()`. To specify a random intercept for a group variable, say `id`, we simply call `brandom(id)` (and could additionally specify the *initial* error variance via `df` or `lambda`). To specify a random slope for another variable, say `time`, within the groups of `id`, one can use `brandom(id, by = time)`, i.e., the first argument determines the grouping (as for the random intercept) and the `by` argument then determines the variable for which the effect is allowed to vary between the groups. In the notation of **nlme** (Pinheiro et al. 2012) and **lme4** (Bates et al. 2011) the random intercept would be specified as `(1 | id)` and the random slope would be specified as `(time - 1 | id)`, i.e., a random slope without random intercept term.

Additional Base-learners in a Nutshell

Tree-based base-learner (per default stumps) can be included in the model using the `btree()` base-learner (Hothorn et al. 2006, 2010). Note that this is the only base-learner which is not fitted using penalized least squares. Radial basis functions (e.g., for spatial effects) can be specified using the base-learner `brad()`. Details on this base-learner are given in (Hofner 2011). Monotonic effects of continuous or ordered categorical variables can be estimated using the `bmono()` base-learner which can also estimate convex or concave effects. Details on monotonic effect estimation and examples of the estimation of boosting models with monotonic effects are given in Hofner et al. (2011b) in the context of species distribution models. The base-learner `bmrf()` implements Markov random fields, which can be used for the estimation of spatial effects of regions (Sobotka and Kneib 2010; Mayr et al. 2012a). With the base-learner `buser()`, one can specify arbitrary base-learners with quadratic penalty. This base-learner is dedicated to advanced users only. Additionally, special concatenation operators for expert users exist in **mboost** that all combine two or more base-learners to a single base-learner: `%+%` additively joins two or more arbitrary base-learners to be treated as one group, `%X%` defines a tensor product of two base-learners and `%O%` implements the Kronecker product of two base-learners. In all cases the degrees of freedom increase compared to the degrees of freedom of the single base-learners (additively in the first, and multiplicatively in the second and third case). For more details on any of these base-learners and for some usage examples see the manual.

3.2.2 Building a Model – or: How to Combine Different Base-learners

In general, the choice of effect for a given covariate has to be based on the subject matter knowledge of the involved researchers. There is no general rule when to use a certain effect. In order to allow researchers to make a decision on which effect to use we exemplified the different modeling types in graphical displays (see Figures 3 to 6).

Once this decision is made, it is relatively easy to estimate the model using the described boosting methodology. Base-learners can be combined in a simple manner to form the desired model. They are combined in the formula as a sum of base-learners to specify the desired model. We will show this using a simple toy example:

```
R> m1 <- gamboost(y ~ bols(x1) + bbs(x2) + bspatial(s1, s2) + brandom(id),
+                 data = example)
```

In this case, a linear effect for `x1`, a smooth (P-spline) effect for `x2`, a spatial effect for `s1` and `s2` are specified together with a random intercept for `id`. This model could be further refined and expanded as shown in the following example:

```
R> m2 <- gamboost(y ~ bols(int, intercept = FALSE) +
+                     bols(x1, intercept = FALSE) +
+                     bols(x2, intercept = FALSE) +
+                     bbs(x2, center = TRUE, df = 1) +
+                     bspatial(s1, s2, knots = list(s1 = 10, s2 = 20)) +
+                     brandom(id) + brandom(id, by = x1),
+                 data = example)
```

Now, with `example$int = rep(1, length(y))`, we specify a separate intercept in the model. In the first formula (`m1`), the intercept was implicitly included in the base-learners. Now we allow the boosting algorithm to explicitly choose and update solely the intercept. Note that therefore we need to remove the implicit intercept from the base-learner for `int` (`intercept = FALSE`) as otherwise we would get a linear base-learner with *two* intercepts which has no unique solution. We can now also remove the intercept from the base-learner of `x1`. This leads to a base-learner of the form $x_1\beta_1$. For the smooth effect of `x2` we now use the decomposition (3). Hence, we specify the unpenalized part as a linear effect without intercept (the intercept is already specified in the formula) and the smooth deviation from the linear effect (with one degree of freedom). Now the boosting algorithm can choose how `x2` should enter the model: (i) not at all, (ii) as a linear effect, (iii) as a smooth effect centered around zero, or (iv) as the combination of the linear effect and the smooth deviation. In the latter case we essentially get the same result as from `bbs(x2)`. The spatial base-learner in the second formula (`m2`) explicitly specifies the numbers of knots in the direction of `s1` (10 inner knots) and `s2` (20 inner knots). This is achieved by a named list where the names correspond to the names of the variables. In total we get $10 \cdot 20 = 200$ inner knots. Usually, one specifies equal numbers of knots in both directions, which requires no named lists. Instead one can simply specify, e.g., `knots = 10`, which results in 10 inner knots per direction (i.e., 100 inner knots in total). Note that, as for the smooth, univariate base-learner of `x2` one could also specify a decomposition for the spatial base-learner:

```
bols(int, intercept = FALSE) +
bols(s1, intercept = FALSE) + bols(s2, intercept = FALSE) +
bols(s1, by = s2, intercept = FALSE) +
bspatial(s1, s2, center = TRUE, df = 1)
```

where `bols(s1, by = s2, intercept = FALSE)` specifies the interaction of `s1` and `s2` (without intercept). Finally, we added a random slope for `x1` (in the groups of `id`) to `m2`. Note that the groups are the argument of the base-learner and the slope variable is specified via the `by` argument.

Case Study (ctd.): Prediction of Body Fat

Until now, we only included linear effects in the prediction formula for body fat of women. It might be of interest to evaluate if there exists also a non-linear relationship between some of the predictors and the DXA measurement of body fat. To investigate this issue, we fit a model with the same predictors as in Garcia et al. (2005) but without assuming linearity of the effects. We apply `gamboost()` with the P-spline base-learner `bbs()` to incorporate smooth effects.

```
R> ## now an additive model with the same variables as lm1
R> gam1 <- gamboost(DEXfat ~ bbs(hipcirc) + bbs(kneebreadth) + bbs(anthro3a),
+                    data = bodyfat)
```

Using `plot()` on a `gamboost` object delivers automatically the partial effects of the different base-learners:

```
R> par(mfrow = c(1,3))     ## 3 plots in one device
R> plot(gam1)              ## get the partial effects
```

From the resulting Figure 7, it seems as if in the center of the predictor-grid (where most of the observations lie), the relationship between these three anthropometric measurements and the body fat is quite close to a linear function. However, at least for `hipcirc`, it seems that there are indications of the existence of a non-linear relationship for higher values of the predictor.

Alternatively, one could apply decomposition (3) for each of the 3 base-learners, as described in Section 3.2.2, to distinguish between modeling alternatives. In this case, we would have a more rigorous treatment of the decision between (purely) linear and non-linear effects if we stop the algorithm at an appropriate iteration m_{stop}.

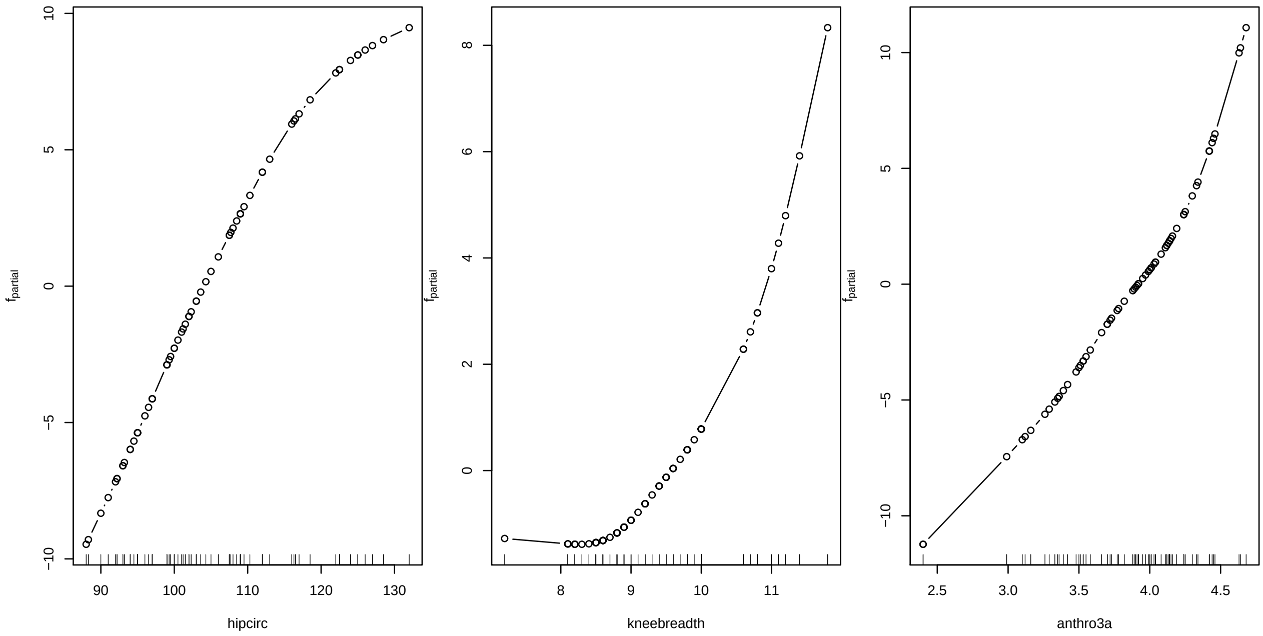

Figure 7: Partial effects of three anthropometric measurements on the body fat of women.

3.3 Early Stopping to Prevent Overfitting

As already discussed in Section 2, the major tuning parameter of boosting is the number of iterations m_{stop}. To prevent overfitting it is important that the optimal stopping iteration is carefully chosen. Various possibilities to determine the stopping iteration exist. One can use information criteria such as AIC[12] to find the optimal model. However, this is usually not recommended as AIC-based stopping tends to overshoot the optimal m_{stop} dramatically (see Hastie 2007; Mayr et al. 2012b). Instead, it is advised to use cross-validated estimates of the empirical risk to choose an appropriate number of boosting iterations. This approach aims at optimizing the prognosis on new data. In **mboost** infrastructure exists to compute bootstrap estimates, k-fold cross-validation estimates and sub-sampling estimates. The main function to determine the cross-validated risk is

```
cvrisk(object, folds = cv(model.weights(object)),
       papply = mclapply)
```

In the simplest case, the user only needs to specify the fitted boosting model as `object`. If one wants to change the default cross-validation method (25-fold bootstrap) the user can specify a weight matrix that determines the cross-validation samples via the `folds` argument. This can be done either by hand or using the convenience function `cv()` (see below). Finally, the user can specify a function of the `lapply` "type" to `papply`. Per default this is either `mclapply` for parallel computing if package **multicore** (Urbanek 2011) is available, or the code is run sequentially using `lapply`. Alternatively, the user can apply other parallelization methods such as `clusterApplyLB` (package **snow** Tierney et al. 2011) with some further effort for the setup (see `?cvrisk`).

The easiest way to set up a variety of weight matrices for cross-validation is the function

```
cv(weights, type = c("bootstrap", "kfold", "subsampling"),
   B = ifelse(type == "kfold", 10, 25))
```

One simply specifies the weights of the originally fitted model (e.g. using the function `model.weights()` on the model) and the `type` of cross-validation. This can be either `"bootstrap"` (default), `"kfold"` (k-fold cross-validation) or `"subsampling"`[13]. The number of cross-validation replicates, per default, is chosen to be 10 for k-fold cross-validation and 25 otherwise. However, one can simply specify another number of replicates using the argument `B`.

To extract the appropriate number of boosting iterations from an object returned by `cvrisk()` (or `AIC()`) one can use the extractor function `mstop()`. Once an appropriate number of iterations is found, we finally

[12] see `?AIC.boost` for further details

[13] the percentage of observations to be included in the learning samples for subsampling can be specified using a further argument in `cv()` called `prob`. Per default this is 0.5.

need to stop the model at this number of iterations. To increase or reduce the number of boosting steps for the model `mod`, one can use the indexing / subsetting operator directly on the model:

```
mod[i]
```

where `i` is the number of boosting iterations. Attention, the subset operator differs in this context from the standard R behavior as it *directly changes the model* `mod`. Hence, there is no need to save `mod` under a new name. This helps to reduce the memory footprint. Be aware that even if you call something like

```
newmod <- mod[10]
```

you will change the boosting iteration of `mod`! Even more, if you now change `mstop` for `newmod`, the model `mod` is also changed (and vice versa)! This said, the good news is that nothing gets lost. If we reduce the model to a lower value of m_{stop}, the additional boosting steps are kept internally in the model object. Consider as an example the following scenario:

- We fit an initial model `mod` with `mstop = 100`.
- We call `mod[10]`, which sets `mstop = 10`.
- We now increase `mstop` to 40 with a call to `mod[40]`.

This now requires *no re-computation* of the model as internally everything was kept in storage. Again the warning, if we now extract anything from the model, such as `coef(mod)`, we get the characteristics of the model with 40 iterations, i.e., here the coefficient estimates from the 40th boosting iteration.

Case Study (ctd.): Prediction of Body Fat

Until now, we used the default settings of `boost_control()` with `mstop = 100` for all boosted models. Now we want to optimize this tuning parameter with respect to predictive accuracy, in order get the best prediction for body fat. Note that tuning `mstop` also leads to models including only the most informative predictors as variable selection is carried out simultaneously. We therefore first fit a model with all available predictors and then tune `mstop` by 25-fold bootstrapping. Using the `baselearner` argument in `gamboost()`, we specify the default base-learner which is used for each variable in the formula for which no base-learner is explicitly specified.

```
R> ## every predictor enters the model via a bbs() base-learner (i.e., as smooth effect)
R> gam2 <- gamboost(DEXfat ~ ., baselearner = "bbs", data = bodyfat,
+                    control = boost_control(trace = TRUE))

[   1] .................................. -- risk: 544.43
[  41] .................................. -- risk: 478.62
[  81] .................
Final risk: 460.34

R> set.seed(123)          ## set seed to make results reproducible
R> cvm <- cvrisk(gam2)    ## default method is 25-fold bootstrap cross-validation
R> ## if package 'multicore' is not available this will trigger a warning
R> cvm

         Cross-validated Squared Error (Regression)
         gamboost(formula = DEXfat ~ ., data = bodyfat, baselearner = "bbs")

      1       2       3       4       5       6       7       8       9      10
109.440  93.905  80.591  69.602  60.134  52.595  46.112  40.802  36.326  32.669
     11      12      13      14      15      16      17      18      19      20
 29.663  27.078  24.993  23.113  21.560  20.403  19.165  18.316  17.598  16.968
     21      22      23      24      25      26      27      28      29      30
 16.488  16.076  15.757  15.471  15.219  15.068  14.970  14.867  14.805  14.747
     31      32      33      34      35      36      37      38      39      40
 14.682  14.686  14.643  14.679  14.682  14.684  14.755  14.803  14.818  14.876
     41      42      43      44      45      46      47      48      49      50
 14.905  14.924  15.004  15.036  15.076  15.107  15.154  15.208  15.238  15.302
     51      52      53      54      55      56      57      58      59      60
```

```
15.319  15.356  15.411  15.461  15.495  15.531  15.576  15.619  15.662  15.712
    61      62      63      64      65      66      67      68      69      70
15.721  15.754  15.808  15.841  15.891  15.905  15.930  15.957  15.991  16.037
    71      72      73      74      75      76      77      78      79      80
16.062  16.106  16.127  16.158  16.187  16.223  16.272  16.277  16.309  16.338
    81      82      83      84      85      86      87      88      89      90
16.368  16.394  16.416  16.436  16.449  16.483  16.520  16.530  16.547  16.585
    91      92      93      94      95      96      97      98      99     100
16.610  16.610  16.624  16.643  16.643  16.684  16.700  16.734  16.749  16.758

        Optimal number of boosting iterations: 33
```

```
R> plot(cvm)    ## get the paths
```

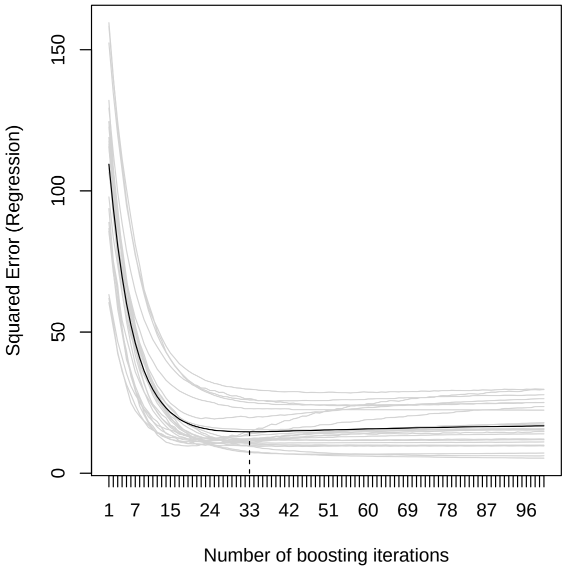

Figure 8: Cross-validated predictive risk with 25-fold bootstrapping.

The plot displays the predictive risk on the 25 bootstrap samples for $m_{\text{stop}} = 1, ..., 100$ (see Figure 8). The optimal stopping iteration is the one minimizing the average risk over all 25 samples. We can extract this iteration via

```
R> mstop(cvm)    ## extract the optimal mstop

[1] 33

R> gam2[ mstop(cvm) ]    ## set the model automatically to the optimal mstop
```

We have now reduced the model of the object `gam2` to the one with only 30 boosting iterations, without further assignment. However, as pointed out above, the other iterations are not lost. To check which variables are now included in the additive predictor we again use the function `coef()`:

```
R> names(coef(gam2)) ## displays the selected base-learners at iteration 30

[1] "bbs(waistcirc, df = dfbase)"   "bbs(hipcirc, df = dfbase)"
[3] "bbs(kneebreadth, df = dfbase)" "bbs(anthro3a, df = dfbase)"
[5] "bbs(anthro3b, df = dfbase)"    "bbs(anthro3c, df = dfbase)"
[7] "bbs(anthro4, df = dfbase)"

R> ## To see that nothing got lost we now increase mstop to 1000:
R> gam2[1000, return = FALSE]  # return = FALSE just supresses "print(gam2)"

[ 101] .................................... -- risk: 431.33
[ 141] .................................... -- risk: 408.8
[ 181] .................................... -- risk: 390.58
```

```
[ 221] ...................................... -- risk: 375.62
[ 261] ...................................... -- risk: 363.12
[ 301] ...................................... -- risk: 352.34
[ 341] ...................................... -- risk: 342.94
[ 381] ...................................... -- risk: 334.52
[ 421] ...................................... -- risk: 326.97
[ 461] ...................................... -- risk: 320.2
[ 501] ...................................... -- risk: 313.96
[ 541] ...................................... -- risk: 308.27
[ 581] ...................................... -- risk: 302.98
[ 621] ...................................... -- risk: 298.09
[ 661] ...................................... -- risk: 293.56
[ 701] ...................................... -- risk: 289.3
[ 741] ...................................... -- risk: 285.3
[ 781] ...................................... -- risk: 281.54
[ 821] ...................................... -- risk: 278
[ 861] ...................................... -- risk: 274.65
[ 901] ...................................... -- risk: 271.45
[ 941] ...................................... -- risk: 268.43
[ 981] ..................
Final risk: 266.98
NULL
```

Although we earlier had reduced to iteration 30, the fitting algorithm started at iteration 101. The iterations 31-100 are not re-computed.

```
R> names(coef(gam2))  ## displays the selected base-learners, now at iteration 1000
```

```
[1] "bbs(age, df = dfbase)"          "bbs(waistcirc, df = dfbase)"
[3] "bbs(hipcirc, df = dfbase)"      "bbs(elbowbreadth, df = dfbase)"
[5] "bbs(kneebreadth, df = dfbase)"  "bbs(anthro3a, df = dfbase)"
[7] "bbs(anthro3b, df = dfbase)"     "bbs(anthro3c, df = dfbase)"
[9] "bbs(anthro4, df = dfbase)"
```

The stopping iteration `mstop` is the main tuning parameter for boosting and controls the complexity of the model. Larger values for `mstop` lead to larger and more complex models, while for smaller values the complexity of the model is generally reduced. In our example the final model at iteration 1000 includes all available variables as predictors for body fat, while the model at the optimal iteration 30 included only five predictors. Optimizing the stopping iteration usually leads to selecting the most influential predictors.

3.4 Specifying the Fitting Problem: The `family`

The list of implemented families in **mboost** is diverse and wide-ranging. At the time of writing this paper, the user has access to sixteen different families. An overview is given in Table 4. A family (most importantly) implements the loss function ρ and the corresponding negative gradient. A careful specification of the loss function leads to the estimation of any desired characteristic of the conditional distribution of the response. This coupled with the large number of base learners guarantees a rich set of models that can be addressed by boosting. We can specify the connection between the response and the covariates in a fairly modular nature such as

$$\xi(y|\mathbf{x}) = \hat{f}_1 + \cdots + \hat{f}_P$$

having on the right hand side any desired combination of base learners. On the left hand side, $\xi(.)$ describes *some* characteristic of the conditional distribution specified by the `family` argument. In the following subsections we discuss major aspects related to the choice of the family.

3.4.1 Families for Continuous Response

Until now the focus was on the conditional mean of a continuous response which is the default setting: `family = Gaussian()`. In this case, our assumption is that $Y|\mathbf{x}$ is normally distributed and the loss function is the negative Gaussian log-likelihood which is equivalent to the L_2 loss

$$\rho(y, f) = \frac{1}{2}(y - f)^2$$

Table 4: An overview on the currently implemented families in **mboost**.

	continuous response	binary response	count data	ordered response	censored data
Standard regression	`Gaussian`				
Median regression	`Laplace`				
Quantile regression	`QuantReg`				
Expectile regression	`ExprectReg`				
Robust regression	`Huber`				
Gamma regression[a]	`GammaReg`				
Logistic regression		`Binomial`			
AdaBoost classification		`AdaExp`			
AUC regression		`AUC`			
Poisson regression			`Poisson`		
Negative binomial model			`NBinomial`		
Proportional odds model				`ProppOdds`	
Proportional hazards model					`CoxPH`
Weibull AFT[b] model					`Weibull`
Log-logistic AFT[b] model					`Loglog`
Log-normal AFT[b] model					`Lognormal`

[a] for non-negative continuous response
[b] accelerated failure time

(see Figure 9(a)). A plain `Gaussian()` call in the console returns its definition

```
R> Gaussian()

         Squared Error (Regression)


Loss function: (y - f)^2
```

The corresponding negative gradient is simply $(y - f)$ whose definition can be displayed on the screen via

```
R> slot(Gaussian(),"ngradient")

function (y, f, w = 1)
y - f
```

If we are interested in the median of the conditional distribution, the `Laplace()` family is the right choice. It implements a distribution free, median regression approach especially useful for long-tailed error distributions. In this case, we use the L_1 loss defined as

$$\rho(y, f) = |y - f|$$

and shown in Figure 9(b). Note that the L_1 loss is not differentiable at $y = f$ and the value of the negative gradient at such points is fixed at zero.

A compromise between the L_1 and the L_2 loss is the Huber loss function shown in Figure 10(a). It is defined as

$$\rho(y, f; \delta) = \begin{cases} (y-f)^2/2 & \text{if } |y-f| \leq \delta, \\ \delta(|y-f| - \delta/2) & \text{if } |y-f| > \delta \end{cases}$$

where the parameter δ limits the outliers which are subject to absolute error loss. The Huber loss can be seen as a robust alternative to the L_2 loss. The user can either specify δ subjectively, e.g. `Huber(d = 2)`, or leave it adaptively chosen by the boosting algorithm (the default behaviour). An adaptive specification of δ, proposed by Friedman (2001), means that each boosting step produces a new $\delta^{[m]}$ matching the actual median of the absolute values of the residuals, i.e.

$$\delta^{[m]} = \text{median}\left(\left|y_i - \hat{f}^{[m-1]}(x_i)\right|, i = 1, \ldots, n\right).$$

Another alternative for settings with continuous response is modeling conditional quantiles through quantile regression (Koenker 2005) – implemented in **mboost** with the `QuantReg()` family (Fenske et al. 2011). The main advantage of quantile regression is (beyond its robustness towards outliers) that it does not rely on any distributional assumptions on the response or the error terms. The appropriate loss function here is the check-function shown in Figure 10(b). For the special case of the 0.5 quantile both `QuantReg(0.5)` and `Laplace()` will lead to median regression. A detailed description of the loss function of quantile regression is given in the Appendix.

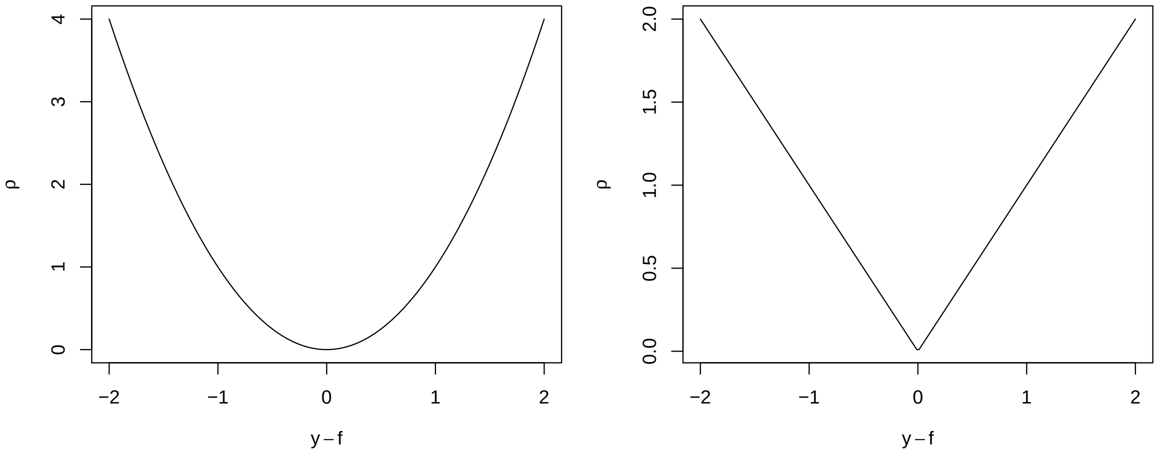

(a) `family = Gaussian()` (b) `family = Laplace()`

Figure 9: The loss function allows flexible specification of the link between the response and the covariates. The figure on the left hand side illustrates the L_2 loss (the default in **mboost**), the figure on the right hand side shows the L_1 loss function.

Case Study (ctd.): Prediction of Body Fat

We reproduce the model of the original publication (Garcia et al. 2005), but instead of modeling the mean we focus on the median:

```
R> ## Same model as glm1 but now with QuantReg() family
R> glm3 <- glmboost(DEXfat ~ hipcirc + kneebreadth + anthro3a, data = bodyfat,
+                    family = QuantReg(tau = 0.5), control = boost_control(mstop = 500))
R> coef(glm3, off2int = TRUE)

(Intercept)     hipcirc kneebreadth    anthro3a
  -63.51643     0.53314     0.77000     7.83509
```

Comparing the results to those of model `glm1` shows that `hipcirc` and `anthro3a` have almost the same influence on the mean as on the median, yet, the magnitude of the effect of `kneebreadth` is considerably smaller in `glm3`. One should note that `mstop` generally needs to be larger for quantile regression, as the single updates are smaller than in the mean regression case. For a discussion see the Appendix.

3.4.2 Families for Binary Response

Analogously to Gaussian regression, the probability parameter of a binary response can be estimated by minimizing the negative binomial log-likelihood

$$\begin{aligned} \rho(y, f) &= -\left[y \,\log\big(\pi(f)\big) + (1-y)\,\log\big(1-\pi(f)\big)\right] \\ &= \log(1+\exp(-2\,\tilde{y}f)) \end{aligned} \tag{4}$$

where $\tilde{y} = 2y - 1$ and $\pi(f) = \mathbb{P}(Y = 1|\mathbf{x})$. For reasons of computational efficiency the binary response $y \in \{0, 1\}$ is converted into $\tilde{y} = 2y - 1$ where $\tilde{y} \in \{-1, 1\}$. In equation (4), $\tilde{y}f$ are the so-called margin values (depicted in Figure 11) which are, roughly speaking, the equivalent of the continuous residuals $y - f$ for the binomial case. This internal re-coding[14] means that the negative binomial log-likelihood loss (`family = Binomial()`) and the exponential loss (`family = AdaExp()`) coincide in their population minimizer (see Bühlmann and Hothorn 2007, Section 3).

Note that the transformation $\tilde{y} = 2y - 1$ changes the interpretation of the effect estimates because now we get the half of the log-odds ratio. One implication is that the `coef()` output is half the estimates that result from `glm()`. This means that the user has to double the coefficients *manually* in order to get the final standard estimates of a logistic regression model.

[14] Note that in **mboost** the response must be specified as a binary `factor`.

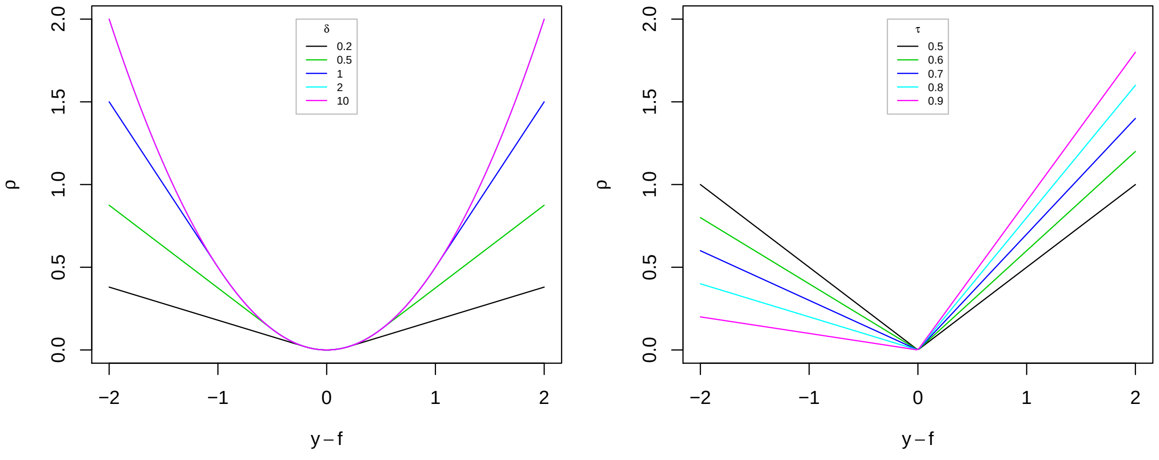

(a) `family = Huber()` (b) `family = QuantReg()`

Figure 10: The Huber loss function on the left hand side is useful when robustness is a concern. In **mboost** it adaptively changes the limit for L_1 penalization of outliers when `d = NULL` (the default). The figure on right hand side illustrates several examples of the check function loss with different quantiles (`tau = 0.5` is the default).

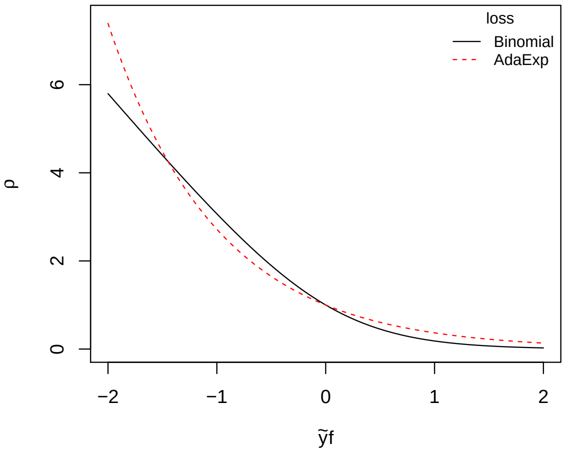

Figure 11: The `Binomial` and the `AdaExp` families as functions of the marginal values $\tilde{y}f$. Since $\tilde{y} \in \{-1, 1\}$, a positive product between $\tilde{y}$ and half the estimated log-odds ratio f means correct classification.

However, **mboost** automatically doubles the logits prior to the reverse probability transformation. This means that calling `predict(fit, newdata, type = "response")` produces the *final* probability estimations. In addition to the logit model, the user can also estimate probit models using `family = Binomial(link = "probit")`.

Alternatively one can also use the exponential loss function $\rho(y, f) = \exp(-\tilde{y}f)$ (`family = AdaExp()`). This essentially leads to the famous AdaBoost algorithm by Freund and Schapire (1996). As can be seen in Figure 11, this loss function is similar to `Binomial()`.

3.4.3 Families for Count Data and Censored Response

In **mboost**, currently two families handle data with count response. The `Poisson()` family uses the negative Poisson log-likelihood with the natural link function $\log(\mu) = \eta$. Alternatively, the negative binomial distribution can be used to model overdispersed data. The negative log-likelihood density of this distribution is implemented in `NBinomial(nuirange = c(0, 100))` where the parameter `nuirange` (accounting for overdispersion) is optimized additionally within each boosting iteration m. One simply minimizes the empirical risk $\mathcal{R}$ over the overdispersion parameter given the current boosting estimate $\hat{f}^{[m]}$ after step 4 of the component-wise gradient boosting algorithm. A thorough introduction and the detailed algorithm is given by Schmid et al. (2010).

Survival models can also be considered in **mboost**: `CoxPH()`, `Weibull()`, `Loglog()` and `Lognormal()` all

implement families for censored data. `CoxPH()` implements the Cox proportional hazards model while the other three families specify accelerated failure time (AFT) models (see Schmid and Hothorn 2008a, for further details).

3.4.4 Further Families

Additionally to the families discussed above, **mboost** implements some further families: `AUC()` can be used to optimize the area under the ROC curve, `GammaReg()` implements the negative Gamma log-likelihood with logarithmic link function, `ExpectReg()` implements expectile regression (Sobotka and Kneib 2010) and `PropOdds()` leads to proportional odds models for ordinal outcome variables (Schmid et al. 2011).

Despite the wide range of available families, users might wish to implement new loss functions. One only needs a differentiable loss function and the corresponding negative gradient $\delta\rho(y, f)\,/\,\delta f$, which both are independent of the base-learners. Using the constructor function `Family()`, one can then easily define new families and thus new estimation problems as we show in the Appendix.

4 Summary

The R package **mboost** offers an easy entry into the world of boosting. It implements a model-based boosting approach that results in interpretable structured additive models of the same form most researchers will feel familiar with. The interfaces of fitting functions are quite similar to standard implementations like `lm()` or `glm()` and are hence relatively easy to use. However, the fitting algorithms of **mboost** additionally offer a high flexibility when it comes to the effect type of potential predictors and the type of risk function to be optimized. There exist a large number of pre-defined families for various risk functions as well as a large number of pre-defined base-learners to specify various types of effects. As **mboost** has a modular nature, both can be combined in any form as desired by the user: For many model classes, **mboost** therefore offers much more modeling alternatives than the classical fitting algorithms. Additionally, the user can also easily extend **mboost** by implementing new families or base-learners.

As seen in the case study, many functions for the manipulation and extraction of the results are available. These allow the user to fit, tune and finally interpret the model. We note that the present tutorial has been designed as an introduction to the basic functionalities of the **mboost** package, highlighting its usage from a practical perspective. Readers who are interested in further illustrations, as well as in a more technical description of the **mboost** package, are referred to the package manual and the vignettes that are found at `http://cran.r-project.org/package=mboost` or in R via `vignette(package = "mboostDevel")`.

References

D. Bates, M. Maechler, and B. Bolker. *lme4: Linear mixed-effects models using S4 classes*, 2011. URL `http://CRAN.R-project.org/package=lme4`. R package version 0.999375-42.

L. Breiman. Arcing classifiers (with discussion). *The Annals of Statistics*, 26:801–849, 1998.

L. Breiman. Prediction games and arcing algorithms. *Neural Computation*, 11:1493–1517, 1999.

L. Breiman. Random forests. *Machine Learning*, 45:5–32, 2001.

P. Bühlmann. Boosting for high-dimensional linear models. *The Annals of Statistics*, 34:559–583, 2006.

P. Bühlmann and T. Hothorn. Boosting algorithms: Regularization, prediction and model fitting (with discussion). *Statistical Science*, 22:477–522, 2007.

P. Bühlmann and B. Yu. Boosting with the L_2 loss: Regression and classification. *Journal of the American Statistical Association*, 98:324–338, 2003.

C. de Boor. *A Practical Guide to Splines*. Springer, New York, 1978.

B. Efron, T. Hastie, L. Johnstone, and R. Tibshirani. Least angle regression. *Annals of Statistics*, 32:407–499, 2004.

P. H. C. Eilers and B. D. Marx. Flexible smoothing with B-splines and penalties (with discussion). *Statistical Science*, 11:89–121, 1996.

J. Fan and J. Lv. A selective overview of variable selection in high dimensional feature space. *Statistica Sinica*, 20:101–148, 2010.

N. Fenske, T. Kneib, and T. Hothorn. Identifying risk factors for severe childhood malnutrition by boosting additive quantile regression. *Journal of the American Statistical Association*, 106(494):494–510, 2011.

N. Fenske, T. Kneib, and T. Hothorn. Identifying risk factors for severe childhood malnutrition by boosting additive quantile regression. *Journal of the American Statistical Association*, 106:494–510, 2011.

Y. Freund and R. Schapire. Experiments with a new boosting algorithm. In *Proceedings of the Thirteenth International Conference on Machine Learning Theory*, pages 148–156, San Francisco, CA, 1996. San Francisco: Morgan Kaufmann Publishers Inc.

J. H. Friedman. Greedy function approximation: A gradient boosting machine. *The Annals of Statistics*, 29: 1189–1232, 2001.

J. H. Friedman, T. Hastie, and R. Tibshirani. Additive logistic regression: A statistical view of boosting (with discussion). *The Annals of Statistics*, 28:337–407, 2000.

A. L. Garcia, K. Wagner, T. Hothorn, C. Koebnick, H.-J. F. Zunft, and U. Tippo. Improved prediction of body fat by measuring skinfold thickness, circumferences, and bone breadths. *Obesity Research*, 13(3): 626–634, 2005.

T. Hastie. Comment: Boosting algorithms: Regularization, prediction and model fitting. *Statistical Science*, 22:513–515, 2007.

T. Hastie and R. Tibshirani. *Generalized Additive Models*. Chapman & Hall, London, 1990.

T. Hastie, R. Tibshirani, and J. Friedman. *The Elements of Statistical Learning: Data Mining, Inference, and Prediction*. Springer, New York, 2 edition, 2009.

B. Hofner. *Boosting in Structured Additive Models*. PhD thesis, Department of Statistics, Ludwig-Maximilians-Universität München, Munich, Germany, 2011.

B. Hofner, T. Hothorn, T. Kneib, and M. Schmid. A framework for unbiased model selection based on boosting. *Journal of Computational and Graphical Statistics*, 20:956–971, 2011a.

B. Hofner, J. Müller, and T. Hothorn. Monotonicity-constrained species distribution models. *Ecology*, 92: 1895–1901, 2011b.

T. Hothorn, K. Hornik, and A. Zeileis. Unbiased recursive partitioning: A conditional inference framework. *Journal of Computational and Graphical Statistics*, 15:651–674, 2006.

T. Hothorn, P. Bühlmann, T. Kneib, M. Schmid, and B. Hofner. Model-based boosting 2.0. *Journal of Machine Learning Research*, 11:2109–2113, 2010.

T. Hothorn, P. Bühlmann, T. Kneib, M. Schmid, and B. Hofner. *mboost: Model-Based Boosting*, 2012. URL http://CRAN.R-project.org/package=mboost. R package version 2.1-3.

T. Kneib, T. Hothorn, and G. Tutz. Variable selection and model choice in geoadditive regression models. *Biometrics*, 65:626–634, 2009.

R. Koenker. *Quantile Regression*. Cambridge University Press, New York, 2005.

A. Mayr, N. Fenske, B. Hofner, T. Kneib, and M. Schmid. Generalized additive models for location, scale and shape for high-dimensional data – a flexible approach based on boosting. *Journal of the Royal Statistical Society: Series C (Applied Statistics)*, 61:403–427, 2012a.

A. Mayr, B. Hofner, and M. Schmid. The importance of knowing when to stop – a sequential stopping rule for component-wise gradient boosting. *Methods of Information in Medicine*, 51:178–186, 2012b. doi: 10.3414/ME11-02-0030.

A. Mayr, T. Hothorn, and N. Fenske. Prediction intervals for future BMI values of individual children - a non-parametric approach by quantile boosting. *BMC Medical Research Methodology*, 12(6):1–13, 2012c.

P. McCullagh and J. A. Nelder. *Generalized Linear Models*. Chapman & Hall, London, 2 edition, 1989.

N. Meinshausen. Quantile regression forests. *Journal Machine Learning Research*, 7:983–999, 2006. ISSN 1533-7928.

J. Pinheiro and D. Bates. *Mixed-Effects Models in S and S-PLUS*. Springer-Verlag, New York, 2000.

J. Pinheiro, D. Bates, S. DebRoy, D. Sarkar, and R Development Core Team. *nlme: Linear and Nonlinear Mixed Effects Models*, 2012. URL http://CRAN.R-project.org/package=nlme. R package version 3.1-103.

R Development Core Team. *R: A Language and Environment for Statistical Computing*. R Foundation for Statistical Computing, Vienna, Austria, 2012. URL http://www.R-project.org. ISBN 3-900051-07-0.

G. Ridgeway. *gbm: Generalized Boosted Regression Models*, 2010. URL http://CRAN.R-project.org/package=gbm. R package version 1.6-3.1.

M. Schmid and T. Hothorn. Flexible boosting of accelerated failure time models. *BMC Bioinformatics*, 9: 269, 2008a.

M. Schmid and T. Hothorn. Boosting additive models using component-wise P-splines. *Computational Statistics & Data Analysis*, 53:298–311, 2008b.

M. Schmid, S. Potapov, A. Pfahlberg, and T. Hothorn. Estimation and regularization techniques for regression models with multidimensional prediction functions. *Statistics and Computing*, 20:139–150, 2010.

M. Schmid, T. Hothorn, K. O. Maloney, D. E. Weller, and S. Potapov. Geoadditive regression modeling of stream biological condition. *Environmental and Ecological Statistics*, 18(4):709–733, 2011.

F. Sobotka and T. Kneib. Geoadditive expectile regression. *Computational Statistics and Data Analysis*, 56: 755–767, 2010. doi: 10.1016/j.csda.2010.11.015.

L. Tierney, A. J. Rossini, N. Li, and H. Sevcikova. *snow: Simple Network of Workstations*, 2011. URL http://CRAN.R-project.org/package=snow. R package version 0.3-7.

S. Urbanek. *multicore: Parallel processing of R code on machines with multiple cores or CPUs*, 2011. URL http://CRAN.R-project.org/package=multicore. R package version 0.1-7.

Appendix: Building Your Own Family

Via the constructor function `Family()`, in **mboost** there exists an easy way for the user to set up new families. The main required arguments are the `loss` to be minimized and the negative gradient (`ngradient`) of the loss. The risk is then commonly defined as the sum of the loss over all observations.

```
Family(ngradient, loss = NULL, risk = NULL, offset = function(y, w)
       optimize(risk, interval = range(y), y = y, w = w)$minimum, ...)
```

We will demonstrate the usage of this function by (re-) implementing the family to fit quantile regression (the pre-defined family is `QuantReg()`). In contrast to standard regression analysis, quantile regression (Koenker 2005) does not estimate the conditional expectation of the conditional distribution but the conditional quantiles. Estimation is carried out by minimizing the check function $\rho_\tau(\cdot)$:

$$\rho_\tau(y_i - f_{\tau i}) = \begin{cases} (y_i - f_{\tau i}) \cdot \tau & (y_i - f_{\tau i}) \geq 0 \\ (y_i - f_{\tau i}) \cdot (\tau - 1) & (y_i - f_{\tau i}) < 0, \end{cases}$$

which is depicted in Figure 10(b). The `loss` for our new family is therefore given as:

```
R> loss =  function(y, f) tau * (y - f) * ((y - f) >= 0) +
+                         (tau - 1) * (y - f) * ((y - f) < 0)
```

The check-function is not differentiable at the point 0. However in practice, as the response is continuous, we can ignore this by defining:

$$-\frac{\partial \rho_\tau(y_i, f_{\tau i})}{\partial f} = \begin{cases} \tau & (y_i - f_{\tau i}) \geq 0 \\ \tau - 1 & (y_i - f_{\tau i}) < 0. \end{cases}$$

The negative gradient of our loss is therefore[15]:

```
R> ngradient = function(y, f, w = NULL)  tau * ((y - f) >= 0) +
+                       (tau- 1) * ((y - f) < 0)
```

Of further interest is also the starting value for the algorithm, which is specified via the `offset` argument. For quantile regression it was demonstrated that the offset may be set to the median of the response (Fenske et al. 2011). With this information, we can already specify our new family for quantile regression:

```
R> OurQuantReg <- function(tau = 0.5){    ## function to include dependency on tau
+    Family(                              ## applying the Family function
+          loss = function(y, f)                              ## loss as above
+                     tau       * (y - f) * ((y - f) >= 0) +
+                     (tau - 1) * (y - f) * ((y - f) < 0) ,
+          ngradient = function(y, f, w = NULL)               ## ngradient as above
+                     tau * ((y - f) >= 0) + (tau - 1) * ((y - f) < 0),
+          offset = function(y, w = NULL)                     ## median as offset
+                     quantile(y, p = 0.5),
+          name = "Our new family for quantile regression" )}
R> OurQuantReg()

         Our new family for quantile regression

Loss function: tau * (y - f) * ((y - f) >= 0) + (tau - 1) * (y - f) * ((y -
     f) < 0)
```

Case Study (ctd.): Prediction of Body Fat

To try our new family we go back to the case study regarding the prediction of body fat. First, we reproduce the model for the median, computed with the pre-defined `QuantReg()` family (see Section 3.4.1), to show that our new family delivers the same results:

[15] The unused weights argument `w` is required to exist by **mboost** when the function is (internally) called. It is hence 'specified' as `NULL`.

```
R> ## Same model as glm3 but now with our new family
R> glm3b <- glmboost(DEXfat ~ hipcirc + kneebreadth + anthro3a, data = bodyfat,
+                     family = OurQuantReg(tau = 0.5),
+                     control = boost_control(mstop = 500))
R> identical(coef(glm3b), coef(glm3))

[1] TRUE
```

To get a better idea of the shape of the conditional distribution we model the median, and the 0.05 and 0.95 quantiles in a small, illustrative example containing only the predictor `hipcirc`:

```
R> glm4a <- glmboost(DEXfat ~ hipcirc, family = OurQuantReg(tau = 0.05), data = bodyfat,
+                     control = boost_control(mstop = 2000))
R> glm4b <- glmboost(DEXfat ~ hipcirc, family = OurQuantReg(tau = 0.5),  data = bodyfat,
+                     control = boost_control(mstop = 2000))
R> glm4c <- glmboost(DEXfat ~ hipcirc, family = OurQuantReg(tau = 0.95), data = bodyfat,
+                     control = boost_control(mstop = 2000))
```

Note that for different quantiles, fitting has to be carried out separately, as τ enters directly in the loss. It is also important that fitting quantile regression generally requires higher stopping iterations than standard regression with the L_2 loss, as the negative gradients which are fitted to the base-learners are vectors containing only small values, i.e., τ and $1-\tau$.

```
R> ord <- order(bodyfat$hipcirc)    ## order the data to avoid problems when plotting
R> plot(bodyfat$hipcirc[ord], bodyfat$DEXfat[ord])                     ## observed data
R> lines(bodyfat$hipcirc[ord], fitted(glm4a)[ord], lty = 2, lwd = 2) ## 0.05 quantile
R> lines(bodyfat$hipcirc[ord], fitted(glm4b)[ord], lty = 1, lwd = 2) ## median
R> lines(bodyfat$hipcirc[ord], fitted(glm4c)[ord], lty = 2, lwd = 2) ## 0.95 quantile
```

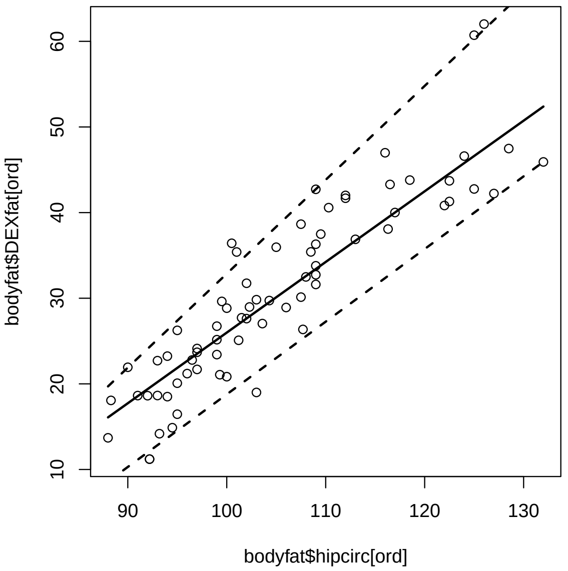

Figure 12: Resulting quantile regression lines, for the median (solid line) and the 0.95 and 0.05 quantiles (upper and lower dashed lines).

The resulting plot (see Figure 12) shows how quantile regression can be used to get a better impression of the whole conditional distribution function in a regression setting. In this case, the upper and lower quantiles are not just parallel lines to the median regression line but adapt nicely to the slight heteroscedasticity found in this data example: For smaller values of `hipcirc` the range between the quantiles is smaller than for higher values. Note that the outer quantile-lines can be interpreted as prediction intervals for new observations (Meinshausen 2006; Mayr et al. 2012c). For more on quantile regression in the context of boosting we refer to Fenske et al. (2011).
